# Current trends and future prospects of drug repositioning in gastrointestinal oncology

**DOI:** 10.3389/fphar.2023.1329244

**Published:** 2024-01-04

**Authors:** Nayeralsadat Fatemi, Mina Karimpour, Hoda Bahrami, Mohammad Reza Zali, Vahid Chaleshi, Andrea Riccio, Ehsan Nazemalhosseini-Mojarad, Mehdi Totonchi

**Affiliations:** ^1^ Basic and Molecular Epidemiology of Gastrointestinal Disorders Research Center, Research Institute for Gastroenterology and Liver Diseases, Shahid Beheshti University of Medical Sciences, Tehran, Iran; ^2^ Department of Molecular Genetics, Faculty of Biological Sciences, Tarbiat Modares University, Tehran, Iran; ^3^ Gastroenterology and Liver Diseases Research Center, Research Institute for Gastroenterology and Liver Diseases, Shahid Beheshti University of Medical Sciences, Tehran, Iran; ^4^ Department of Environmental, Biological and Pharmaceutical Sciences and Technologies (DiSTABiF), Università degli Studi della Campania “Luigi Vanvitelli”, Caserta, Italy; ^5^ Institute of Genetics and Biophysics (IGB) “Adriano Buzzati-Traverso”, Consiglio Nazionale delle Ricerche (CNR), Naples, Italy; ^6^ Department of Genetics, Reproductive Biomedicine Research Center, Royan Institute for Reproductive Biomedicine, ACECR, Tehran, Iran

**Keywords:** gastrointestinal cancers, therapeutic strategies, drug repurposing, colorectal cacner, pancreatic cancer, liver cancer

## Abstract

Gastrointestinal (GI) cancers comprise a significant number of cancer cases worldwide and contribute to a high percentage of cancer-related deaths. To improve survival rates of GI cancer patients, it is important to find and implement more effective therapeutic strategies with better prognoses and fewer side effects. The development of new drugs can be a lengthy and expensive process, often involving clinical trials that may fail in the early stages. One strategy to address these challenges is drug repurposing (DR). Drug repurposing is a developmental strategy that involves using existing drugs approved for other diseases and leveraging their safety and pharmacological data to explore their potential use in treating different diseases. In this paper, we outline the existing therapeutic strategies and challenges associated with GI cancers and explore DR as a promising alternative approach. We have presented an extensive review of different DR methodologies, research efforts and examples of repurposed drugs within various GI cancer types, such as colorectal, pancreatic and liver cancers. Our aim is to provide a comprehensive overview of employing the DR approach in GI cancers to inform future research endeavors and clinical trials in this field.

## 1 Introduction

Gastrointestinal (GI) cancers, a group of malignancies occurring within the digestive system including colorectal cancer (CRC), esophageal cancer (EC), gastric cancer (GC), liver cancer (LC), and pancreatic cancer (PC) comprised a substantial proportion of global cancer cases. They were responsible for approximately 27.7% of cancer cases (5.5 million cases out of 18.1 million worldwide) and 35.8% of global cancer-related deaths in 2020 (Sung et al., 2021). GI cancers have different global prevalence and mortality rates. CRC is the most frequently occurring type, ranking third in prevalence and the second in cancer-related deaths. GC, LC and EC rank as the fifth, sixth and eighth most commonly diagnosed cancers, with mortality rates ranking fourth, third and sixth, respectively. PC which has a poor prognosis with low curability rates compared to the other GI cancers, ranks 12th in frequency and the seventh in cancer death (Ferlay et al., 2021).

GI cancers exhibit diverse incidence patterns across regions, with PC and CRC being more prevalent in Europe and Northern America, and GC, LC, and EC in Asia. Interestingly, the countries with high human development indicators (HDIs) report a higher incidence of PC cases (Li et al., 2021a; Huang et al., 2021). In addition, the incidence of GI cancers is influenced by several factors such as lifestyle habits and dietary choices as well as age, with elderly males having a higher risk of incidence and mortality compared to females. This emphasizes the complex interplay of environmental and biological factors in the development of GI cancers (Sung et al., 2021).

The existing standard treatment options for GI cancers comprise surgery, chemotherapy, radiation therapy, targeted therapy, and immunotherapy (Nakayama et al., 2013; Anwanwan et al., 2020; Biller and Schrag, 2021; Joshi and Badgwell, 2021). Although combination regimens offer higher response rates and improved survival rates than single-agent therapy, it is crucial to consider the toxicity profile of these regimens closely (Chakraborty and Rahman, 2012; Miller et al., 2016; Jin and Mills, 2020). Reducing GI cancer mortality involves identifying and implementing more efficient therapy strategies that lead to superior prognosis and/or fewer side effects. Although the discovery and development of novel drugs are crucial for halting and reversing disease effects, they demand substantial funding, broad experimentation, and subsequent investigations into efficacy, pharmacokinetics, and toxicity. Moreover, only a small percentage of these drugs, approximately 5%, undergo clinical trials which may be approved for clinical use after successful results from the three phases of clinical trials (Gupta et al., 2013). Thus, developing new drugs is a costly and time-consuming procedure that requires extensive resources and expertise.

**FIGURE 1:**
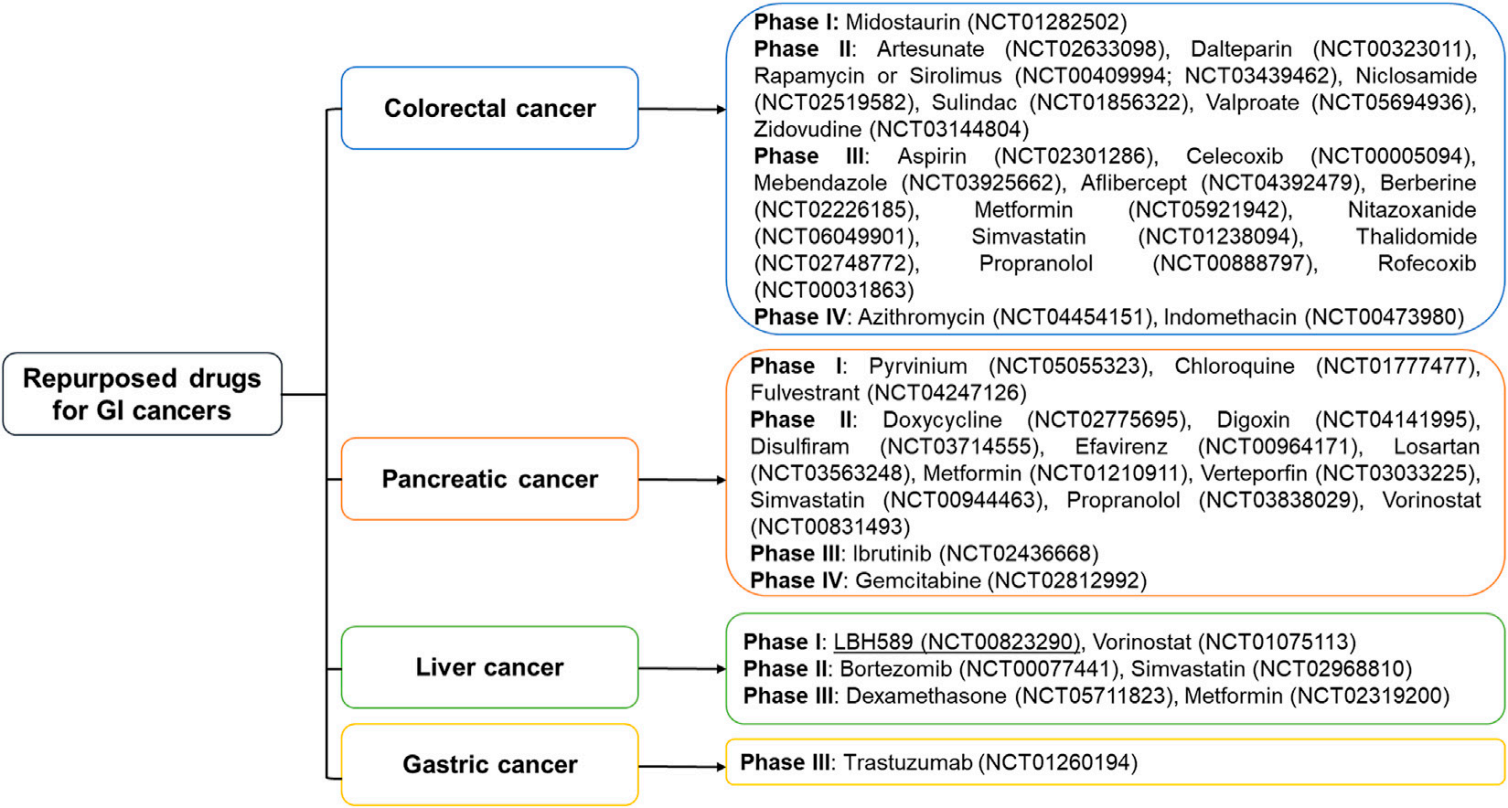
Repurposed drugs currently being evaluated against gastrointestinal cancers in various clinical phases. Metformin, simvastatin, and propranolol are entering phases 2 and 3 to treat more than 2 gastrointestinal cancers. Two drugs indomethacin and azithromycin for colorectal cancer, and gemcitabine for pancreatic cancer have reached phase IV.

Drug repurposing (DR), also known as drug repositioning, is an alternative and promising strategy for cancer treatment. It involves exploring the potential therapeutic applications of already approved drugs or withdrawn/outdated agents in the clinic (Tables 1–4; Supplementary Table S2; Figure 1). DR offers a substantial asset in drug development, uses the prior knowledge of the pharmacodynamics, pharmacokinetics and toxicity of already approved drugs which have undergone extensive animal and human studies (Gupta et al., 2013; Bertolini et al., 2015). Accordingly, repurposed compounds can be authorized for use in cancer treatment and other therapeutic applications rapidly, relying on the established safety profiles and efficacy data. Moreover, the regulatory approval process for repurposed drugs is often faster and less expensive, making DR an attractive alternative strategy for drug development (Chong and Sullivan, 2007). In this review, we aim to provide a critical overview of the current therapies for GI cancers, as well as examples of repositioned components, and the most promising candidate for drugs repurposing in GI cancers.

**TABLE 1:** List of repurposed drugs proposed for targeting colorectal carcinoma.

Drug name	Mechanism of action	Original indication	Proposed indication	Reported targets/pathways	Status	References
Adapalene	Inhibition of proliferation, and induction of cell cycle arrest	Acne	Colorectal cancer	PTGS2	*In vitro* (LoVo and DLD1 cell lines), and *in vivo* (mouse xenograft model)	Shi et al. (2015)
Aflibercept	Inhibition of angiogenesis	Neovascular (wet) age-related macular degeneration, macular edema following retinal vein occlusion, diabetic macular edema, and diabetic retinopathy	Metastatic colorectal cancer	VEGFA	Clinical trial: Phase 3 (NCT04392479)	Li et al. (2018a)
Amantadine	Inhibition of cell proliferation, and induction of apoptosis	Parkinson	Colorectal cancer	Endoretroviruses (HERV-WE1, HERV-FRD1, HERV-31, and HERV-V1)	*In vitro* (HCT8 cell line)	Díaz-Carballo et al. (2015)
Artesunate	Induction of apoptosis and cytotoxicity	Antimalarial	Colorectal cancer	Downregulation of *β*-catenin	Clinical Trial: Phase 2 (NCT02633098)	Kumar et al. (2019)
Aspirin	Inhibition of tumor proliferation	Analgesia	Colorectal cancer, Gastrointestinal, esophageal cancer, etc.	COX-1/2, ANXA1-NF–κB axis, CDX2, COMMD1–RelA axis	Clinical trial: Phase 3 (NCT02301286)	Frouws et al. (2017)
Azithromycin	Inhibition of autophagy	Antibiotic	Colorectal cancer	Inhibition of autophagy by upregulating p62 and LC-3B	Clinical trial: Phase 4 (NCT04454151)	Qiao et al. (2018)
Berberine	Inhibition of invasion and metastasis	A chemical found in some plants and is typically used to treat bacterial diarrhea	Gastric, colorectal, lung cancer, etc.	Ephrin-B2, MMP-2/MMP-9, EMT, miR-101, VEGF	Clinical trial: Phase 3 (NCT02226185)	Yu et al. (2014)
Captopril	Inhibition of cell proliferation and metastasis	Hypertension	Colorectal cancer	Angiotensin converting enzyme (ACE)	*In vivo* (mouse model)	Neo et al. (2007)
Celecoxib	Inhibition of proliferation through apoptosis	Pain and inflammation	Familial adenomatous polyps	COX-2	Clinical trial: Phase 3 (NCT00005094)	Grösch et al. (2001)
Chloroquine and related-derivatives	Inhibition of tumor growth and induction of apoptosis	Malaria, rheumatoid arthritis	Colorectal cancer	Autophagy, PPT1, TLR9/NFκB	*In vitro* (HCT116, HT29, and CT26) and *in vivo* (mouse xenograft model)	Anselmino et al. (2023)
Clarithromycin	Inhibition of cell growth, autophagy and angiogenesis	Antibiotic	Colorectal cancer	Inhibition of autophagy by targeting hERG1, PI3K	*In vitro* (HCT116 and LS174T, HEK293, and HT29 cell lines), and *in vivo* (mouse xenograft model)	Petroni et al. (2020)
Dalteparin	Inhibition of angiogenesis	Anticoagulant	Colorectal cancer	VEGFA	Clinical trial: Phase 2 (NCT00323011)	Agnelli et al. (2022)
Dapagliflozin	Reduction of cell adhesion and proliferation	Antihyperglycemic	Colorectal cancer	ADAM10, DDR1, cellular interaction with Collagen types I and IV Increased Erk phosphorylation	*In vitro* (HCT116 cell line)	Okada et al. (2020)
Diclofenac	Inhibition of cell proliferation via MYC-dependent and -independent mechanisms	Pain of osteoarthritis	Colorectal cancer	Bcl-2, COX-1, COX-2, MCP-1, MIP-1α and VEGF	*In vitro* (sw480, and Caco-2 cell lines) and *in vivo* (rat model)	Kaur and Sanyal, (2011)
Disulfiram	Reprogramming energy metabolism	Alcohol dependence	Colorectal cancer	NF-κB, NPL4	*In vitro* (H630, DLD-1 and RKO cell lines)	Wang et al. (2003)
Doxycycline	Induction of apoptosis and inhibition of proliferation and invasive potential	Antibiotic	Colorectal cancer	Inhibition of matrix metalloproteinases Activation of caspase-3, -8, and -9 Release of cytochrome c and Bax translocation	*In vitro* (LS174T, and HT29 cell lines)	Onoda et al. (2004)
Ebselen	Inhibition of tumor growth	Multifunctional compound	Colorectal cancer	ATG4B, autophagy and tumor suppression	*In vitro* (HCT116, and RKO cell lines), and *in vivo* (mouse xenograft model)	Xie et al. (2022)
Efavirenz	Induction of apoptosis	Anti-retroviral (anti-HIV drug)	Colorectal cancer	Activation of the phosphorylation of p53	*In vitro* (HCT-15 cell line)	Hecht et al. (2013)
Fluoxetine	Inhibition of colitis-associated tumorigenesis, dysplasia and angiogenesis	Antidepressant	Colorectal cancer	Inhibition of NF-κB activation and IKK phosphorylation Cell-cycle arrest at G0/G1 Enhanced p27 expression Reduced VEGF expression	*In vitro* (HT29 cell line), *in vivo* (mouse model)	Kannen et al. (2012)
Gemifloxacin	Inhibition of cell migration and invasion	Antibiotic	Colorectal cancer	Inhibition of NF-κB activation Inhibition of TNF-α, IL-6, IL-8, and VEGF	*In vitro* (SW620, and LoVo cell lines)	Kan et al. (2013)
Indinavir	Inhibition of tumor growth	Anti-retroviral (anti-HIV drug)	Colorectal cancer	Proteasome-independent block of angiogenesis and matrix metalloproteinases	*In vitro* (SW480 cell line), *in vivo* (mouse xenograft model)	Toschi et al. (2011)
Indomethacin	Induction of G1 arrest and apoptosis	Rheumatic disease	Colorectalcancer	Shc-ERK axis, PKCζ-p38-DRP1 axis, Wnt/β-catenin	Clinical trial: Phase 4 (NCT00473980)	Lin et al. (2019), Mazumder et al. (2019), Bahmad et al. (2022)
Irbesartan	Inhibition of metastasis	Hypertension	Colorectal cancer	Angiotensin receptor	*In vivo* (mouse xenograft model)	Neo et al. (2007)
Ivermectin	Inhibition of proliferation and induction of apoptosis	Antihelmintic drug	Colorectal cancer	WNT-TCF signaling	*In vitro* (SW480 and SW1116 cell lines)	Zhou et al. (2021)
Linagliptin	Inhibition of metastasis	Type-2 diabetes	Colorectal cancer	Rb/Bcl-2/p53	*In vitro* (HCT 116 cell line), and *in vivo* (mouse xenograft model)	Li et al. (2020b)
Lovastatin	Inhibition of cancer progression and metastasis	Antilipidemic	Colorectal cancer	Inhibition of MACC1	*In vitro* (SW480 cell line) and *in vivo* (mouse model)	Xiao et al. (2022)
Mebendazole	Inhibition of metastasis	Antihelmintic drug	Colorectal cancer	MYC and COX2 pathways	Clinical trial: Phase 3 (NCT03925662)	Nygren and Larsson (2014), Hegazy et al. (2022)
Mefloquine	Induction of apoptosis and growth arrest	Antimalarial	Colorectal cancer	Inhibition of NF-κB activation	*In vitro* (HT-29, HCT116, RKO, SW620 and Lovo cell lines), and *in vivo* (mouse xenograft model)	Xu et al. (2018)
Metformin	Reprogramming energy metabolism	Obese type 2 diabetes	Colorectal cancer	AMPK, PI3K-mTOR pathways, BACH1	Clinical trial: Phase 3 (NCT05921942)	Higurashi and Nakajima (2018)
Midostaurin	Inhibition of cell growth and cell cycle arresting	A protein kinase inhibitor that has been developed for the treatment of acute myeloid leukemia, myelodysplastic syndrome and advanced systemic mastocytosis	Rectal cancer	cGAS, STING, IRF3, IFNAR1, Trex-1, c-Kit, and Flt3	Clinical trial: Phase 1 (NCT01282502)	Lai et al. (2022)
Nebivolol	Inhibition of tumor growth	Hypertension and other indications	Colorectal cancer	Inhibition of mitochondrial respiration by decreasing the activity of Complex I of the respiratory chain	*In vitro* (HCT116 cell line), and *in vivo* (mouse xenograft model)	Nuevo-Tapioles et al. (2020)
Niclosamide	Inhibition of invasion and metastasis	Antihelminthic drug	Colorectal cancer	Wnt/β-catenin, STAT3, NF-κB	Clinical trial: Phase 2 (NCT02519582)	Wang et al. (2019)
Nitazoxanide	Induction of G1 arrest, Modulation of angiogenesis and metabolism	Anti-Parasite	Colorectal cancer	mTOR	Clinical trial: Phase 3 (NCT06049901)	Senkowski et al. (2015), Ripani et al. (2020)
Oxiconazole	Inhibition of tumor growth	Antifungal agent	Colorectal cancer	Inhibiting autophagy through downregulation of peroxiredoxin-2 (PRDX2)	*In vitro* (HCT116, SW480, RKO, DLD-1, SW620, LoVo cell lines), and *in vivo* (mouse xenograft model)	Shi et al. (2022a)
Parecoxib	Prevention of inflammation and tumor-promotion	Pain	Colorectal cancer	COX2, PTGS2	*In vitro* (HCT116 and HT29 cell lines), and *in vivo* (mouse xenograft model)	Xiong et al. (2015)
Perhexiline	Induction of apoptosis, and reduction of cell viability	Anti-anginal	Colorectal cancer	-	*In vitro* (SW480, SW620, HCT116, HT29 and COLO205 cell lines), and *in vivo* (Patient-derived organoids)	Dhakal et al. (2022)
Propranolol	Induction of apoptosis	Hypertension	Colorectal cancer	Activating autologous CD8^+^ T cells and decreasing the expression of p-AKT/p-ERK/p-MEK, inhibiting the expression of p-ERK.	Clinical trial: Phase 3 (NCT00888797)	Liao et al. (2020)
Raltegravir	Inhibition of invasion	Anti-retroviral (anti-HIV drug)	Colorectal cancer	Blockage of fascin-1	*In vitro* (HCT-116 and DLD-1), and *in vivo* (mouse xenograft model)	Alburquerque-González et al. (2021)
Rapamycin or Sirolimus	Inhibition of cell proliferation, invasion, and angiogenesis, and induction of apoptosis	Immunosuppressant, anti-restenosis agent, Prevention of kidney transplant rejection	Rectum, and colorectalcancers	mTOR and associated signaling networks, CHOP-dependent DR5 induction on 4E-BP1 dephosphorylation Suppressed FBXW7 loss-driven EMT	Clinical trial: Phase 2 (NCT00409994) and (NCT03439462)	Gulhati et al. (2009), Mussin et al. (2017)
Ritonavir	Induction of apoptosis and inhibition of angiogenesis	Anti-retroviral (anti-HIV drug)	Colorectal cancer	Inhibition proteolytic degradation and accumulation of p21 Decreased production of TNF-α, IL-6, IL-8, and VEGF Increased expression of heme oxygenase-1	*In vitro* (DLD-1 cell line)	Mühl et al. (2004)
Rofecoxib (Withdrawn) (Phase 3)	Inhibition of mMetastasis	Osteoarthritis, rheumatoid arthritis, juvenile rheumatoid arthritis, acute pain conditions, migraine, and dysmenorrhea	Colorectal cancer	COX-2	Clinical trial: Phase 3 (NCT00031863)	Midgley et al. (2010)
Simvastatin	Inhibition of metastasis	Dyslipidemia	Colorectal cancer	KRAS	Clinical trial: Phase 3 (NCT01238094)	Lee et al. (2011)
Spiperone	Induction of apoptosis	Schizophrenia	Colorectal cancer	Activating phospholipase C, disrupting intracellular calcium balance, inducing irreversible endoplasmic reticulum stress, causing lipid metabolism changes, damaging the Golgi apparatus	*In vitro* (HCT116, SW620, HCT8, and MDA-MB-231 cell lines)	Antona et al. (2022)
Sulindac	Prevention of inflammation and tumor-promotion, and induction of apoptosis	Pain, swelling, and joint stiffness from arthritis	Colorectal cancer	PTGS2, Cyclin G2	Clinical trial: Phase 2 (NCT01856322)	Wang et al. (2022)
Thalidomide	Inhibition of angiogenesis	Sedative, antiemetic	Colorectal cancer	Various proangiogenic factors, VEGF receptor, NF-κB	Clinical trial: Phase 3 (NCT02748772)	Sakamoto and Maeda (2010), Zhang and Luo (2018)
Tolfenamic acid	Inhibition of cell proliferation, metastasis, and induction of apoptosis	Migraine	Colorectal cancer	Cyclin D, cyclin E, Cdk2, E2F-1, c-Myc, Mmp7, S100a9, Nppb and Aldh1a3, PTGS, VEGF, survivin, XIPA	*In vitro* (HCT116 and LoVo cell lines)	Jeong et al. (2013)
Valproate	Reduction of cell proliferation and cytotoxicity enhancement	Antipsychotic	Colorectal cancer	Histone hyperacetylation Relief of HDAC-mediated transcriptional repression	Clinical trial: Phase 2 (NCT05694936)	Jeong et al. (2013), Patel and Patel (2018)
Zidovudine	Induction of apoptosis, and cell cycle arrest	Anti-retroviral (anti-HIV drug)	Colorectal cancer	Increased expression of the p53-Puma/Bax/Noxa pathways Activation of the p53-p21 pathway	Clinical trial: Phase 2 (NCT03144804)	Falcone et al. (1997)

**TABLE 2:** List of repurposed drugs proposed for targeting pancreatic cancer.

Drug name	Mechanism of action	Original indication	Proposed indication	Reported targets/pathways	Status	References
AM580	Increasing tumor sensitivity to chemotherapy	Acute promyelocytic leukemia	Pancreatic ductal adenocarcinoma	Upregulation of Meflin expression	*In vitro* (Pancreatic Stellate Cells)	Iida et al. (2022)
Bazedoxifene	Inhibition of cell viability and migration	Selective estrogen receptor modulator	Pancreatic ductal adenocarcinoma	Inhibition of STAT3 activation mediated by interleukin 6 (IL-6) and 11 (IL-11)	*In vitro* (AsPC-1, PANC-1, HPAF-II, BxPC-3, HPAC, Capan-1 cell lines), and *in vivo* (mouse xenograft model)	Wu et al. (2016)
Carglumic acid	Induction of apoptosis	Hyperammonemia	Pancreatic ductal adenocarcinoma	COX-1/2, ANXA1-NF–κB axis, CDX2, COMMD1–RelA axis	*In vitro* (Capan1, AsPc1/luc, and PanO2/luc cell lines), and *in vivo* (mouse model)	Chen et al. (2015a)
(Hydroxy)-Chloroquine	Inhibition of proliferation	Malaria	Pancreatic Cancer	Inhibition of autophagy in PSCs through reduced IL-6 expression and ECM protein production, Reduction of metastatic PC cells, ERK/MAPK inhibitors, Inhibition of CXCL12/CXCR4 signaling, reduced phosphorylation of ERK and STAT3, downregulation of Hedgehog signaling	Clinical trial: Phase 1 (NCT01777477)	Samaras et al. (2017)
		Systemic Lupus				
		Erythematosus				
		Rheumatoid arthritis				
Doxycycline	Induction of apoptosis, and cell cycle arrest	Antibiotic	Pancreatic ductal adenocarcinoma	Impairment of mitochondrial biogenesis and oxidative phosphorylation, downregulation of PAR1/FAK/PI3K/AKT signaling	Clinical trial: Phase 2 (NCT02775695)	Liu et al. (2020c)
Digoxin	Induction of apoptosis	Atrial fibrillation, atrial flutter, and heart failure	Pancreatic Cancer	Nrf2 inhibitor	Clinical trial: Phase 2 (NCT04141995)	Zhou et al. (2019b)
Disulfiram	Induction of autophagy-dependent apoptosis, and ER stress	Drugs used in addictive disorders, Chronic alcoholism	Pancreatic ductal adenocarcinoma	Activation of the IRE1a-XBP1 pathway upregulation of p27 Inhibition of the NF-kB signaling pathway and downregulate stemness-related genes (HER2, c-myc and SOX9), Promotion of aponecrosis death pathways in K-Ras mutant PC cells, activation of the ER stress/IRE1α-XBP1 pathway	Clinical trial: Phase 2 (NCT03714555)	Zhang et al. (2019)
Efavirenz	Inhibition of cell proliferation, and induction of apoptosis	HIV infection	Pancreatic Cancer	ROS production and mitochondrial membrane depolarization, phosphorylation of both ERK1/2 and p38 MAPK stress pathways	Clinical trial: Phase 2 (NCT00964171)	Hecht et al. (2018)
Emetine, Ouabain	Induction of cancer cell death	Emetine; Anti-protozoal, Ouabain; The cardiac glycoside	Pancreatic ductal adenocarcinoma	Interfering in hypoxia response	*In vitro* (ASPC-1, and PANC-1 cell lines), *in vivo* (patient-derived organoid)	Hirt et al. (2022)
Fulvestrant	This study focused on bioinformatic approaches	Fulvestrant; Metastatic breast cancer, Midostaurin; AML	Pancreatic ductal adenocarcinoma	Fulvestrant; target *ESR1,* Midostaurin; target *PRKA*	Clinical trial: Phase 1 (NCT04247126)	Mugiyanto et al. (2022)
Gemcitabine	Inhibition of DNA synthesis and induction of apoptosis	Antiviral	Bladder cancer; Pancreatic ductal adenocarcinoma; Non-small cell lung cancer; Ovarian cancer; Breast cancer	_	Clinical trial: Phase 4 (NCT02812992), FDA approved	Rebelo et al. (2021)
Haloperidol	Inhibition of proliferation by promoting ER stress, and induction of apoptosis	Psychosis	Pancreatic Cancer	*DUSP6*	*In vitro* (MIA PaCa-2, and PANC-1 cell lines)	Kim et al. (2012), Heer et al. (2022)
Ibrutinib	Induction of apoptosis	Antineoplastic agents (protein kinase inhibitors)	Pancreatic ductal adenocarcinoma	Mast cell-dependent antifibrotic effect	Clinical trial: Phase 3 (NCT02436668)	Massó-Vallés et al. (2015), Gunderson et al. (2016), Overman et al. (2020)
Itraconazole	Induction of apoptosis, and Inhibition of cell proliferation	Antifungal	Pancreatic ductal adenocarcinoma	Inhibition of TGF-β/SMAD2/3 signaling ROS production and mitochondrial membrane depolarization, Bak-1 activation, TGF-β/SMAD2/3 signaling suppression	*In vitro* (CFPAC-1, MiaPaCa-2, Panc-1, and BxPC-3 cell lines), and *in vivo* (mouse xenograft model)	Chen et al. (2018), Jiang et al. (2019)
Losartan	Inhibition of cell proliferation	Angiotensin II receptor antagonist	Pancreatic ductal adenocarcinoma	Inhibition of collagen I synthesis, Blockade of AT1R leading to inhibition of VEGF synthesis, increasing CD8^+^ T cells, decreasing IL-1β, TANs and Tregs, inhibiting aberrant TGF-β activity	Clinical trial: Phase 2 (NCT03563248)	Diop-Frimpong et al. (2011)
Metformin	Inhibition of proliferation	Antidiabetic	Pancreatic ductal adenocarcinoma	Inhibition of mTOR, STAT3 and TGF-β1/Smad2/3 signaling suppression of insulin/IGF-I receptor activation and downstream signaling mediators IRS-1 and Akt Activation of AMPK	Clinical trial: Phase 2 (NCT01210911)	Karnevi et al. (2013), Nair et al. (2014), Kordes et al. (2015)
Nitroxoline	Induction of cell cycle arrest and apoptosis	Antiviral (Nelfinavir), Antibiotic (Nitroxoline)	Pancreatic ductal adenocarcinoma	ROS production, DNA damage response, mitochondrial depolarization and deregulation of cytosolic iron homeostasis	*In vitro* (AsPC-1, BxPC-3, and Capan-2 cell lines)	Veschi et al. (2018), Veschi et al. (2020)
Olanzapine	Inhibition of tumor proliferation	Antipsychotic	Pancreatic ductal adenocarcinoma	Inhibition of surviving in CSCs	*In vitro* (PANC-1, and PSN-1 cell lines)	Sanomachi et al. (2017)
Parbendazole	Induction of apoptosis, and cell cycle arrest	Anthelmintic	Pancreatic ductal adenocarcinoma	Apoptosis induction, DNA damage, cell cycle arrest and alterations of tubulin distribution	*In vitro* (AsPC-1, and Capan-2 cell lines)	Florio et al. (2019)
Pentoxifylline	Inhibition of metastasis	Vasodilator	Pancreatic ductal adenocarcinoma	Reduction in collagen I and downregulation of alpha-smooth muscle actin and connective tissue growth factor Inhibition of chitinase 3-like-1	*In vitro* (BxPC3, and PANC-1 cell lines)	Xavier et al. (2021)
Pimavanserin	Induction of apoptosis	Parkinson disease psychosis	Pancreatic Cancer	Abrogation of Akt/Gli1 signaling cascade leading to the downregulation of Oct-4, SOX2 and NANOG cancer stem cell markers	*In vitro* (AsPC1, BxPC3, MIAPaCa2, and PANC1 cell lines), and *in vivo* (mouse xenograft model)	Ramachandran and Srivastava (2020)
Pimozide	Induction of ER stress, cell cycle arrest, apoptosis and activation of the UPR	Antipsychotic	Pancreatic ductal adenocarcinoma	Inhibition of DRD2	*In vitro* (BxPC-3, Panc-1, MiaPaCa-2, Capan-1, and CFPAC-1 cell lines), and *in vivo* (mouse xenograft model)	Jandaghi et al. (2016)
Pirfenidone	Inhibition of proliferation and promotion of cell cycle arrest	Antifibrotic	Pancreatic ductal adenocarcinoma	Suppression of desmoplasia through regulation of PSCs, Suppression of PDGF-A, HGF, periostin, collagen type I and fibronectin, Cell cycle arrest and upregulation of p21 of PDAC cells, Inhibition of CHI3L1 and FN1, Downregulation of collagen I and TGF-β, Inhibition of fibronectin	*In vitro* (SW1990 cell line), and *in vivo* (mouse xenograft model)	Ji et al. (2017), Gao et al. (2019)
Propranolol	Induction of apoptosis	Hypertension	Pancreatic Cancer	Inhibiting the expression of NF-kB, AP-1 and CREB, as well as the expression of MMP-9, MMP-2 and VEGF target genes, decreasing Fz1, Wnt-1 and vimentin expression, downregulation of α7nAChR, ERK1/2 and p-CREB	Clinical trial: Phase 2 (NCT03838029)	Al-Wadei et al. (2009), Zhang et al. (2009), Zhang et al. (2010), Li and Xu (2019)
Pyrvinium	Inhibition of tumor cells in nutrient-depleted condition by targeting mitochondria	Anthelmintic	Pancreatic ductal adenocarcinoma	Inhibition of mitochondrial function, the WNT pathway, and cancer stem cell renewal	Clinical trial: Phase 1 (NCT05055323)	Schultz et al. (2021); Ponzini et al. (2023)
Ritonavir	Induction of apoptosis and cell cycle arrest	Antiviral	Pancreatic ductal adenocarcinoma	Induction of apoptosis and cell cycle arrest, through Inhibition of E2F-1 and AKT pathway, suppression of Akt and Rb phosphorylation	*In vitro* (BxPC-3, MIA PaCa-2, and PANC-1 cell lines)	Batchu et al. (2014)
Somatostatin	Inhibition of angiogenesis and cell migration	Neuroendocrine inhibitor	Pancreatic Cancer	Cytotoxic, somatostatin receptors (SSTR) targeted therapy	*In vitro* (Capan-1, Capan-2, CAV, MIA PaCa-2, and Panc-1 cell lines), and *in vivo* (pancreatic tumor xenografts)	Li et al. (2005)
Simvastatin	Inhibition of metastasis	Dyslipidemia	Pancreatic ductal adenocarcinoma	KRAS	Clinical trial: Phase 2 (NCT00944463)	Liu et al. (2020b)
Trifluoperazine	Induction of apoptosis and necroptosis	Antipsychotic	Pancreatic ductal adenocarcinoma	Impairment of mitochondrial and ER homeostasis, induction of apoptosis and necroptosis and activation of the UPR	*In vitro* (MiaPaCa-2), and *in vivo* (patient-derived xenograft)	Huang et al. (2019)
Verteporfin	Induction of apoptosis	Antineovascularization agent (Verteporfin), Sensitizers in photodynamic therapy (protoporphyrin IX)	Pancreatic ductal adenocarcinoma	Activation of apoptosis via TAp73 activation, Inhibition of thioredoxin reductase, Inhibition of Hippo/YAP signaling pathway	Clinical trial: Phase 2 (NCT03033225)	Huggett et al. (2014); Hanada et al. (2021)
Vorinostat	Inhibition of proliferation	HDAC inhibitors	Pancreatic Cancer	*FBP1*	Clinical trial: Phase 2 (NCT00831493)	Emamzaden et al. (2022)

**TABLE 3:** List of repurposed drugs proposed for targeting hepatocellular carcinoma.

Drug name	Mechanism of action	Original indication	Proposed indication	Reported targets/pathways	Status	References
Amiodarone	Inhibition of proliferation and induction of apoptosis, and autophagy	Antiarrhythmic	Hepatocellular Carcinoma	*mTOR*	*In vitro* (Hep 3B, HepG2 and Hu-H7 cell lines), and *in vivo* (HBx-transgenic mice)	Favoulet et al. (2001); Lan et al. (2014)
Atovaquone	Induction of apoptosis	Pneumonia	Hepatocellular Carcinoma	DNA double-stranded breaks	*In vitro* (HepG2, Hep3B, and Huh7 cell lines), and *in vivo* (mouse xenograft model)	Gao et al. (2018)
Bortezomib	Inhibition of proliferation	26S proteasome inhibitor	Hepatocellular Carcinoma	*FBP1*	Clinical trial: Phase 2 (NCT00077441)	Jin et al. (2017)
Canagliflozin	Inhibition of Cell proliferation, differentiation, stress response, and induction of apoptosis	Oral hypoglycemic	Hepatocellular Carcinoma	*ERK*, *p38*, *AKT*	*In vitro* (Huh7, HepG2, and HLE cell lines), and *in vivo* (patient-derived xenograft)	Kaji et al. (2018)
Dexamethasone	Inhibition of proliferation	Synthesized glucocorticoid	Hepatocellular Carcinoma	*FBP1*	Clinical trial: Phase 3 (NCT05711823)	Zhao et al. (2021)
Fenofibrate	Metabolic reprogramming	Antihypercholesterolemia	Hepatocellular Carcinoma	*PPARa*, *AKT*, *CTMP*	*In vitro* (Hep3B, Li7, Huh7, and HepG2 cell lines), and *in vivo* (mouse xenograft model)	Yamasaki et al. (2011), Chen et al. (2023)
Genistein	Inhibition of glycolysis and induction of mitochondrial apoptosis	Inhibits *HK2*	Hepatocellular Carcinoma	Downregulates *HIF-1α*, therefore inactivating *GLUT1* and *HK2,* enhances the antitumor effect of sorafenib in sorafenib-resistant HCC cells	*In vitro* (CC-LM3, SMMC-7721, Hep3B, Bel-7402, and Huh-7 cell lines), and *in vivo* (mouse xenograft model)	Li et al. (2017b)
Guanabenz acetate	Induction of apoptosis	Antihypertensive	Hepatocellular Carcinoma	DNA damage-inducible, *p34*, eukaryotic initiation factor 2α	*In vitro* (NU398, SNU423, SNU 449, SNU475, Huh7 cell lines), and *in vivo* (patient derived xenograft)	Kang et al. (2019)
Ketoconazole	Inhibition of tumor growth, invasion, and metastasis	Antifungal	Hepatocellular Carcinoma	*PTGS2*	*In vitro* (cell line-derived xenograft), and *in vivo* (patient-derived xenograft)	Chen et al. (2019a); Chen et al. (2019b)
LBH589	Inhibition of proliferation	HDAC inhibitors	Hepatocellular Carcinoma	*FBP1*	Clinical trial: Phase 1 (NCT00823290)	Yang et al. (2017)
Linagliptin	Immune destruction	Oral hypoglycemic	Hepatocellular Carcinoma	*ADORA3*	*In vitro* (HepG2, and Huh7 cell lines)	Ayoub et al. (2018)
Metformin	Inhibition of proliferation	Oral hypoglycemic	Hepatocellular Carcinoma	*KLF6*/*p21*, *AMPK*	Clinical trial: Phase 3 (NCT02319200)	Vacante et al. (2019)
Niclosamide ethanolamine	Inhibition of proliferation and angiogenesis, induction of apoptosis	Anthelmintic	Hepatocellular Carcinoma	*STAT3*	*In vitro* (HepG2, Huh7, Hep3B, Hep40, PLC/PRF/5, SNU-398, SNU-449, SNU-182, SNU-475 and SNU-423 cell lines), and *in vivo* (patient-derived xenograft)	Chen et al. (2017a)
Obeticholic acid	Inhibition of proliferation and angiogenesis and metastasis, induction of apoptosis	Primary biliary cholangitis	Hepatocellular Carcinoma	IL-6/STAT3 pathway	*In vitro* (HepG2, Huh7, and SNU-449 cell lines), and *in vivo* (orthotopic liver tumor model)	Attia et al. (2017), Gou et al. (2022)
Simvastatin	Controlling of tumor growth via metabolism reprogramming	Antihypercholesterolemia	Hepatocellular Carcinoma	*AMPK*, *STAT3*	Clinical trial: Phase 2 (NCT02968810)	Wang et al. (2017), Dehnavi et al. (2021)
Tranylcypromine	Inhibition of proliferation	LSD1 inhibitor	Hepatocellular Carcinoma	*FBP1*	Asian cohort study: population-based nested case-control study	Chen et al. (2017c)
Valproate	Prevention of proliferation via Reactive Oxygen Species (ROS)-mediated cytotoxicity	Antiepileptic	Hepatocellular Carcinoma	*HDAC*	*In vitro* (HepG2 cell line)	Rithanya and Ezhilarasan (2021)
Vorinostat	Blocking growth promoting signal transduction pathways and inhibition of proliferation	Histone deacetylase inhibitor	Hepatocellular Carcinoma	*ERK/NF-κB signaling*	Clinical trial: Phase 1 (NCT01075113)	Gordon et al. (2019)

**TABLE 4:** List of repurposed drugs proposed for targeting Gastric cancer.

Drug name	Mechanism of action	Original indication	Proposed indication	Reported targets/pathways	Status	Ref
Sulfasalazine	Inhibition of proliferation and metastasis and induction of ferroptosis	Rheumatoid arthritis	Gastric cancer	_	Clinical trial (EPOC1205)	Shitara et al. (2017)
Trastuzumab	Inhibition of cell proliferation	Oncology drug, HER2 positive breast cancer	HER2 positive Gastric cancer	HER2	Approved (NCT01260194)	Rose and Bekaii-Saab (2011)
6-Thioguanine	Induction of cell death	Antimetabolite, guanine analog, acute and chronic myelogenous leukemias	Gastric cancer	Ferroptosis inducer	*In vitro* and *in vivo* (xenograft mouse model)	Zhang et al. (2022a)

## 2 Current gastrointestinal cancers therapies, challenges, and limitations

The treatment approaches for GI cancer patients are diverse and contingent upon factors such as the patient’s performance status, medical comorbidities, cancer type, stage, potential side effects and overall health (Joshi and Badgwell, 2021; Catalano et al., 2022; Ducreux et al., 2023). Treatment strategies encompass adjuvant chemotherapy, adjuvant chemoradiotherapy, preoperative chemoradiotherapy, endoscopic/colonoscopy resection, surgical resection, and perioperative chemotherapy. A comprehensive therapy regimen involves a combination of suitable treatment options to effectively address GI cancers (Sugarbaker, 2005).

Targeted therapy serves as a viable treatment option within various therapeutic regimens for GI cancers. Notably, for GC, HER2-targeted therapy (trastuzumab) and anti-angiogenesis therapy (ramucirumab) are major targeted therapies employed (Joshi and Badgwell, 2021). Ramucirumab is also considered as a targeted therapy alternative for patients with EC who have not responded well to initial treatment approaches (Fuchs et al., 2014). Erlotinib, an epidermal growth factor receptor (EGFR) blocking agent, has obtained Food and Drug Administration (FDA) approval for advanced PC patients (Moore et al., 2007). In the treatment of CRC, the commonly employed anti-angiogenesis agent, bevacizumab, in combination with chemotherapy can prolong the survival rate of advanced CRC patients (Xiong et al., 2021). Unresectable or metastatic hepatocellular carcinoma (HCC) patients commonly undergo a combination of anti-angiogenesis targeted therapy and immunotherapy as the predominant treatment strategies for HCC, while systemic chemotherapy proves ineffective in such cases (Greten et al., 2008).

Immunotherapy, aimed at restoring immune system function, represents another line of therapy for patients with advanced GI cancer. An alternative treatment for metastatic CRC, HER2-positive EC, and advanced GC patients who exhibit Programmed Cell Death Ligand 1 (PD-L1) or microsatellite instability-high (MSI-H) biomarkers and are resistant to chemotherapy is pembrolizumab, an anti-PD-1 antibody, which functions by targeting the PD-1 receptor on tumor cells and preventing their evasion from the immune system. In addition, dostarlimab is another alternative option for the treatment of PCs with MSI-H or deficient mismatch repair (dMMR). Nivolumab either alone or in combination with ipilimumab is employed to treat adults with MSI-H or dMMR who have metastatic and drug-resistant CRC or EC (Zhang X. et al., 2022; Teixeira Farinha et al., 2022). It is worth noting that dostarlimab and nivolumab act upon the PD-1 receptor, whereas ipilimumab specifically targets CTLA-4. For additional information on this subject, refer to the Supplementary Material, Supplementary Table S1.

Despite the range of therapy options available for GI cancers, the high mortality rate highlights the limited efficiency of current treatments for these malignancies (Sung et al., 2021). Several challenges impede the improvement of existing therapeutic strategies, including chemotherapy resistance, tumor heterogeneity, late diagnosis, and limited efficiency of certain treatments, all contributing to treatment failure in GI cancer patients (Au et al., 2017; Parikh et al., 2019; Raziq et al., 2020). The development of drug resistance involves a complex multi-step process influenced by a variety of contributing factors. Numerous studies have shown that intensified DNA repair, apoptosis or autophagy disorders, epithelial-mesenchymal transition, inactivation of drug-metabolizing enzymes, and changes in expression or activity of membrane transporters are potential factors that promote chemotherapy resistance (Zheng, 2017). Furthermore, In the context of GI cancers, the unique characteristics of certain gastrointestinal tumors pose significant challenges to their effective treatment. For instance, the pancreas is anatomically situated in a hard-to-reach location, leading to difficulties in early diagnosis and the absence of effective screening techniques presents a significant challenge in detecting PC during its initial stages (McGuigan et al., 2018). Despite endeavors to implement personalized treatments, PC has not exhibited convincing outcomes comparable to those seen in other cancer types (Chantrill et al., 2015). The efficiency of radiotherapy in EC is limited due to the prevention of TAZ (Transcriptional Activator with PDZ-Binding Motif) ubiquitination and degradation (He et al., 2021). Furthermore, epigenetics and non-coding RNAs play a critical role in EC-related multidrug resistance and affect the effectiveness of therapies. In fact, EC’s susceptibility to recurrence, metastasis, and drug resistance development after first-line treatment, highlights the urgent requirement for optimizing the medicine regimen (Liu et al., 2021; Wei et al., 2021).

## 3 Drug repurposing

DR, is a strategic approach aimed at investigating alternative therapeutic applications of existing approved medications, beyond their originally intended uses. This method offers significant advantages in improving treatment outcomes, primarily by circumventing several essential stages of drug development. This results in reduced expenditure, shorter clinical trial durations, and mitigated risks associated with clinical trial failures due to adverse reactions (Ashburn and Thor, 2004; Chong and Sullivan, 2007; Zamami et al., 2017).

Repurposing FDA-approved medications is an efficient and inexpensive method to address oncology needs, including cancer treatment and reducing problems from existing anticancer treatments or radiation therapy (Ciociola et al., 2014). The drug-repurposing strategy has various advantages, including a shorter development period (usually 3–5 years), reduced costs (under $10 million), and higher success rates than original drug research (Morgan et al., 2011). Furthermore, DR can offer numerous benefits in overcoming therapeutic challenges by targeting various components both inside and outside of cancer cells (Würth et al., 2016). The concomitant use of multiple drugs that can target different tumor subtypes simultaneously can be a remarkable plan to overcome tumor heterogeneity (Li and Jones, 2012). Moreover, DR reveals the anticancer potential of non-oncology drugs with fewer side effects than traditional chemotherapy, making it a valuable option for cancer treatment (Crawford, 2014; Ferioli et al., 2018). Repositioned drugs can be used in combination with regular chemotherapy, and some demonstrate selectivity in causing cytotoxicity to cancer cells while sparing non-cancerous cells (Foglietta et al., 2021).

**FIGURE 2:**
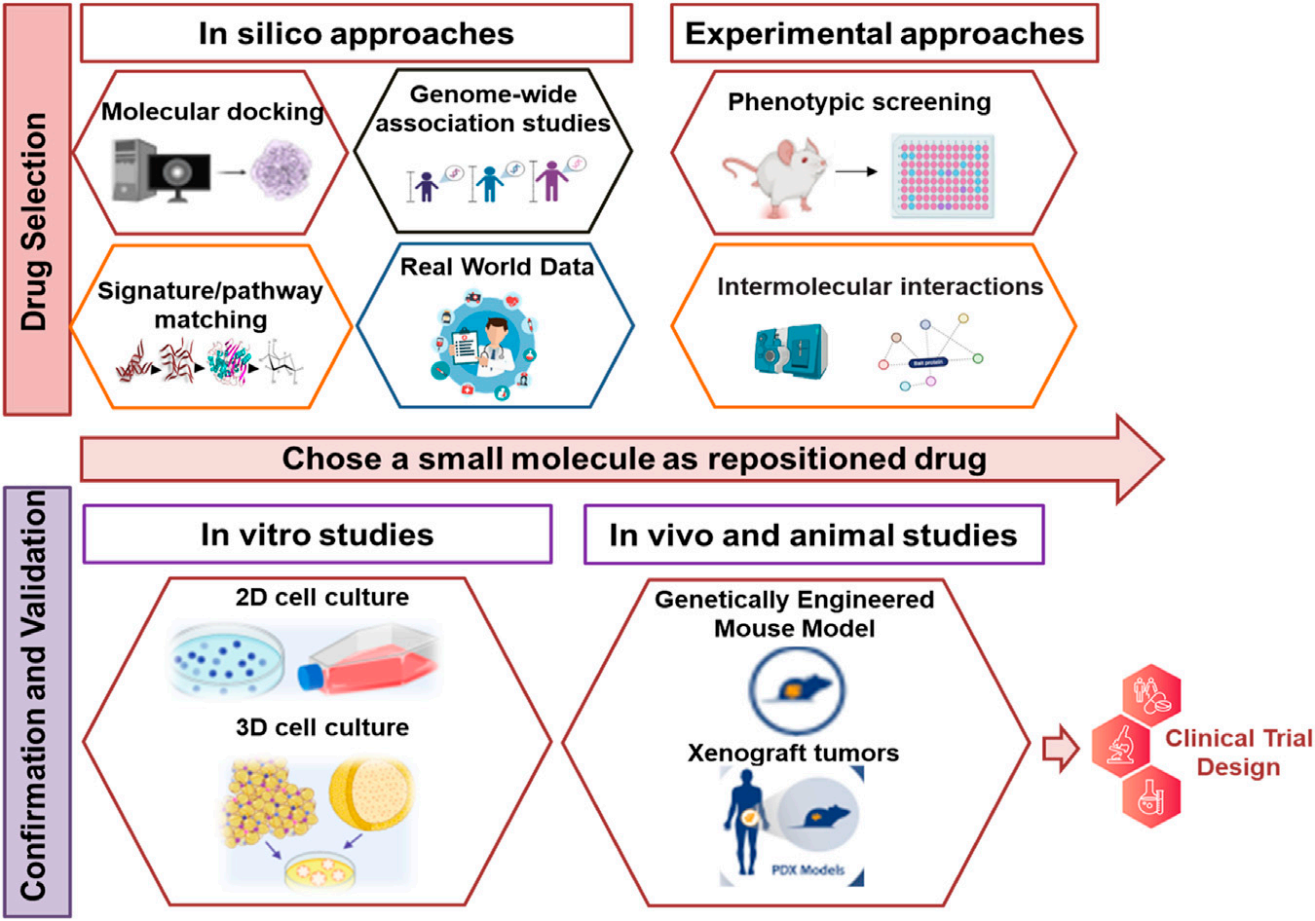
The key stages of drug repurposing. The process of DR begins with the use of computational or experimental techniques to select candidate drugs. Subsequently, these selected drugs are subjected to additional validation through potential evidence and additional experimental methods. Compounds that successfully pass through this rigorous evaluation then advance to clinical trials to obtain the FDA approval. Upon approval, these drugs are introduced to the market and their labeling is updated to reflect their new applications.

Drug repositioning involves a multi-step process. Initially, drug selection can start with *in silico* methods like molecular docking, pathway matching, and genome-wide association studies to create a ranked list of compounds. The next step is secondary analysis, which includes experimental techniques to refine and prioritize these compounds. Tertiary analysis aims to validate these compounds using cell cultures and animal models. Finally, the chosen drugs are advanced to clinical trials, and successful ones are repurposed for new uses (Shameer et al., 2015; Kulkarni et al., 2023). The key stages of DR are elucidated in (Figure 2).

## 4 Approaches used for drug repurposing in cancer therapy

To evaluate the repurposing of an existing drug as a potentially effective anti-cancer agent, a mechanistic assessment of the drug’s action in preclinical models is crucial and requires systematic approaches (El-Hachem et al., 2017; Nagaraj et al., 2018). These approaches can be classified into two categories, computational and experimental, which use available data and biochemical experiments, respectively, to explore the potential of repurposing existing drugs as novel cancer treatments (Luo et al., 2017; Fernández-Torras et al., 2019). Indeed, successful DR is contingent upon the integrated and synergistic use of both approaches (Mottini et al., 2021; Palve et al., 2021).

### 4.1 Computational approaches

Data-driven computational approaches involve the systematic analysis of data from different sources such as gene expression, chemical structure, genotype or proteomic data, or electronic health records (EHRs), which lead to the establishment of hypotheses on DR (Hurle et al., 2013). Carla Mottini et al. (2021) thoroughly reviewed computer-aided DR strategies for cancer therapy, offering examples of this approach in cancer studies within their article (Mottini et al., 2021). Future research should improve advanced computational tools, like machine learning and artificial intelligence, to improve therapeutic efficacy and safety prediction (Prasad and Kumar, 2021). Furthermore, computational methods can be used to address current anticancer medications or radiation therapy issues in addition to cancer treatment (Dalwadi et al., 2023). The most commonly used computational approaches are discussed below.

#### 4.1.1 Molecular docking

Molecular docking, a structure-based computational strategy, predicts complementarity of binding sites between the ligand (drug) and the receptor (target) (Hurle et al., 2013; Knapp, 2018; Mottini et al., 2021; Palve et al., 2021). If prior knowledge is available about a receptor target involved in cancer, multiple drugs could be investigated against that specific target, or drug libraries can be screened for a collection of target receptors to identify new interactions suitable for repurposing (Honarparvar et al., 2014; Nero et al., 2014). Dakshanamurthy and others performed molecular fit computations on a list of 3,671 FDA-approved drugs against 2,335 crystal structures of human proteins. They experimentally validated that mebendazole, an anti-parasitic agent, shows significant potential to bind to the vascular endothelial growth factor receptor 2 (VEGFR2) and effectively block angiogenesis (Dakshanamurthy et al., 2012). Furthermore, it has been predicted that levosimendan, a heart failure drug, could serve as a potential inhibitor of several kinases including RIO Kinase 1 (RIOK1), through ligand-binding site comparison and protein-ligand docking. Levosimendan shows anti-cancer activity against various cancers by directly inhibiting RIOK1 and RNA processing enzymes (Lim et al., 2019). Similarly, Arulanandam CD et al. (2021) suggested itraconazole as a better inhibitor of platelet-derived growth factor receptor alpha (PDGFRA) compared to other antifungal drugs for treating gastrointestinal stromal tumors (GISTs) (Arulanandam et al., 2021).

#### 4.1.2 Genome-wide association studies

Genome-Wide Association Studies (GWAS) aim to identify variants associated with common genetic disorders, thereby providing new insights into the biology of the disease. The novel associations between genes and cancer through GWAS and Phenome-Wide Association Studies (PheWAS) enable the identification of new targets for existing drugs leading to the repositioning of drugs (Zhang et al., 2015; Sud et al., 2017; Kim et al., 2018). Grover et al. (2015) employed a bioinformatics strategy to match gene targets for coronary artery disease (CAD) with drug information collected from three different drug–target databases (DrugBank, Therapeutic Target Database, and PharmGKB) to identify potential therapeutics for repositioning toward treatment of CAD (Grover et al., 2015). Another valuable resource for medication indications, called Medication Indication Resource (MEDI), can validate the plausibility of inferring novel drug indications with clinical potential (Wei et al., 2013; Bejan et al., 2015). Furthermore, a genome-wide positioning systems network algorithm uncovered the antitumor activity of ouabain, an approved drug for cardiac arrhythmia and heart failure, in lung adenocarcinoma cells (Cheng et al., 2019).

GWAS results may pose challenges when applied to DR due to several reasons. Firstly, GWAS signals in gene-rich loci, where linkage disequilibrium commonly occur, can complicate the identification of the associated genes and their specific variants. Secondly, the direction of the gene variant’s effect may not be immediately apparent, necessitating functional studies to determine whether they act as activators or suppressors for disease control (Sanseau et al., 2012). Moreover, GWAS results may not provide detailed pathogenic information on genetic diseases (Wang and Zhang, 2013). It is important to acknowledge that understanding of the human genome is continually evolving, and new genes may continue to be discovered (Willyard, 2018). Therefore, careful consideration and further research are necessary when leveraging GWAS data for DR endeavors.

#### 4.1.3 Signature or pathway matching

The signature matching method represents an innovative and promising approach within the realm of DR, as it involves comparing the distinct characteristics of a drug with those of other drugs, disease or clinical phenotype (Hieronymus et al., 2006; Keiser et al., 2009). The drug signatures can be derived from three different sources of data including omics data, chemical structures and adverse event profiles (Pushpakom et al., 2019). As a specific example, a study employed an optimal approach including two novel benchmarking standards, namely, area under the curve (AUC)-based standard and Kolmogorov-Smirnov (KS) statistic-based standard for signature-based DR and reported homoharringtonine (HHT) as a potential agent in the treatment and prevention of LC (Yang et al., 2022). Furthermore, various omics studies (transcriptomic, proteomic, or metabolomics) in the field of cancer not only provide large-scale data for supporting DR through applying advanced bioinformatics, but also expand our knowledge of hallmarks of cancer at the molecular level (Fernandez-Banet et al., 2016; Chen B. et al., 2020). Chemical structures serve as another type of signature matching used in DR; where the comparison of the chemical features of each drug with others helps identify possible chemical similarities that could signify shared biological activity (Pushpakom et al., 2019). A recent research employed this method along with other conventional DR methods to create a multilayer network algorithm. This algorithm was used to rank drugs that possibly can be repurposed for various types of cancer, including GI cancers and to explore new therapeutic possibilities for existing drugs (Cheng et al., 2021).

The unique adverse event profile of a drug can serve as a proxy for its related phenotypic effects, and drugs with similar side effects may act on the same target protein or pathway (Dudley et al., 2011; Cheng et al., 2021). In addition, if a drug’s phenotypic response resembles that of a disease, it may indicate that the drug and disease share pathways and physiological mechanisms (Pushpakom et al., 2019). Pathway and network-based approaches have been broadly used to identify drugs or drug targets with the potential for repurposing (Smith et al., 2012). Moreover, despite some of the targets identified by GWAS or other analytical networks appearing potent for drug targets, many of these genes may not be ideal in practical drug targeting applications. In such circumstances, a pathway-based approach can provide insights into genes upstream or downstream of the GWAS-associated target and could be considered as potential repurposing opportunities (Greene and Voight, 2016). In a recent study, the network-wide association study (NetWAS) technique has been employed, combining GWAS-identified genetic variant information with tissue-specific functional networks to identify disease-relevant genes more accurately than GWAS alone. Using NetWAS on the concept of hypertension and incorporating drug–target data from the DrugBank, Greene et al. (2015) observed an expansion in the number of the top target genes for the anti-hypertensive drugs compared to the GWAS (Greene et al., 2015). Additionally, pathway analysis of gene expression data obtained from a wide range of studies of human viral respiratory diseases identified 67 signaling pathways which may play important roles in respiratory viral infections (Smith et al., 2012). In summary, DR candidates can be identified through construction of drug or disease networks using gene expression patterns, protein interactions, disease pathology or GWAS data along with the signature matching studies to complement the network analysis approach (Iorio et al., 2010, 2013).

#### 4.1.4 Real World Data

Real World Data (RWD) includes information from electronic health records (EHRs) of patients, characterized by large and complex datasets (Booth et al., 2019; Eichler et al., 2019). EHRs store patient and population health information in digital format, providing a wealth of data on patient outcomes (Miriovsky et al., 2012; Luhn et al., 2019). While diagnostic and pathophysiological data including experimental and drug prescribing data are structured, a significant portion of EHR data, such as clinical descriptions of patient symptoms and signs as well as imaging data remains unstructured (Khozin et al., 2017; Booth et al., 2019). Both structured and unstructured data from patients can serve as valuable sources to identify consistent signals for drug repurposing (Hurle et al., 2013; Low et al., 2017).

The retrospective clinical trial analysis is a commonly used computational approach based on RWD, with data commonly extracted from EHRs (Gray et al., 2019; Zheng et al., 2020). Sildenafil is an interesting example of retrospective clinical analysis which led to the repurposing of a candidate molecule (Ashburn and Thor, 2004). A classical example of repurposing a noncancer drug for cancer therapy through retrospective clinical analysis is metformin, which has shown to reduce cancer mortality in a dose-dependent manner (Sadeghi et al., 2012; Chaiteerakij et al., 2016). Furthermore, Xu et al. (2015) conducted a clinical cohort study using EHRs from Vanderbilt University Medical Center and Mayo Clinic, confirming the favorable effects of metformin in cancer survival including breast, colorectal, lung and prostate cancers in independent populations (Xu et al., 2015). Other successful examples of repurposing opportunities through retrospective clinical and/or pharmacological analyses include raloxifene in breast cancer, aspirin in CRC, propranolol in osteoporosis, and valproate in acute myeloid leukemia and glioblastoma (Cavalla and Singal, 2012; Happold et al., 2016; Lübbert et al., 2020).

### 4.2 Experimental approaches

#### 4.2.1 Phenotypic screening

Phenotypic screening, a direct method of DR, involves identifying compounds based on their effects in model systems without prior knowledge of candidate drug targets (Moffat et al., 2014, 2017; Kim et al., 2019). This approach typically uses a wide range of cell-based *in vitro* assays in a 96- or 384-well format (Sala et al., 2010). Iljin and others (2009) have performed high-throughput cell-based screening of a library including 4,910 drug-like small molecules against prostate cancer and non-malignant prostate epithelial cell lines using proliferation as the primary phenotypic criteria. They found an anticancer effect of disulfiram, a medication used to treat alcohol abuse, which was subsequently validated through genome-wide-gene expression studies (Iljin et al., 2009). Moreover, whole animal screening assays can be employed for DR which offer insights into efficient anticancer drugs, as well as pharmacokinetic and organ-toxicity potential results (Yadav et al., 2016; Baxendale et al., 2017). Cousin et al. (2014) evaluated 39 FDA-approved medications using a zebrafish model for tobacco dependence treatment and identified compounds like apomorphine and topiramate that modulate the behavioral effects of nicotine and ethanol in this model (Cousin et al., 2014). Similarly, over 26,000 small molecules were evaluated for their efficacy against leukemia using a genetically engineered T-cell reporting zebrafish model and discovered the remarkable activity of lenaldekar, against various hematologic neoplasms (Clements and Traver, 2012; Ridges et al., 2012).

#### 4.2.2 Intermolecular interactions

Proteomic techniques including affinity chromatography and mass spectrometry reveal protein-protein interactions based on the intermolecular force. These techniques have been used to identify binding partners for various drugs, thus facilitating DR due to an experimentally based pharmacological analysis (Brehmer et al., 2005; Shantikumar et al., 2015). In this approach, drug treatments are administered to cells or animals, followed by a deep proteome analysis to identify protein changes (Savitski et al., 2018). An early successful example of this technique involves the validation of more than 20 cellular targets for the epidermal growth factor receptor kinase inhibitor gefitinib using mass spectrometry (Brehmer et al., 2005).

The Cellular Thermo Stability Assay (CETSA) has been introduced as a method based on altered protein thermal stabilization/destabilization in response to ligand binding which can predict drug targets when combined with thermal proteome profiling (Martinez Molina et al., 2013; Li J. et al., 2020; Mateus et al., 2020; Perrin et al., 2020). Additionally, chemical genetic approaches such as kinase drug discovery also rely on intermolecular forces and can provide insights into the relationship between binding and drug efficacy (Sloane et al., 2010; Cong et al., 2012; Wong et al., 2016). These findings can be interpreted quickly into new clinical applications or to improve drug resistance outcomes which are near-inevitable phenotypic responses to protein kinase inhibitors for the cancer treatment (Carter et al., 2005; Bago et al., 2016). Karaman et al. (2008) used a competition binding assay *in vitro* to evaluate 38 protein kinase inhibitors against a panel of 317 pathologically significant human protein kinases. Their analysis identified 3,175 binding interactions, revealing that some drugs such as sorafenib and dasatinib showed higher affinity to other kinase targets than their known target (Karaman et al., 2008). The development of chemical-genetic approaches, particularly non-kinase targets of small molecules planned to inhibit kinases is becoming increasingly recognized (Munoz, 2017) resulting in new repurposing opportunities for cancer treatment, such as the use of anthelmintic drug niclosamide to treat Zika virus infection (Xu et al., 2016) and the potential to treat drug-resistant pathogens (Sun et al., 2016).

### 4.3 Experimental approaches to validate repurposed drugs

In order to understand the mechanism of action of repurposed drugs for cancer treatment, it is often essential to evaluate and validate their effects within a comprehensive system that considers safety, dosage and toxicity before advancing to clinical trials. This evaluation can be accomplished using various models, which are divided into two main categories: *in vivo* and *in vitro* models. These models should be reproducible, cost-effective, and quickly constructed (Würth et al., 2016).

#### 4.3.1 *In vitro* studies

In *in vitro* tumor models, cancer cell lines are the predominant choice, followed by primary cells, although these models may also incorporate immune cells, stem cells, and stromal cells alongside cancer cells (Martinez-Pacheco and O’Driscoll, 2021). When using these cell types for detecting repositioning activity, *in vitro* assays offer several advantages including the ability to examine multiple substances with distinct mechanisms of action across a wide concentration-effect range, depending on the throughput of the experiment. Furthermore, these assays provide direct knowledge about potential new disease settings and allow testing of different drugs with novel mechanisms of action (Wilkinson and Pritchard, 2015).

*In vitro* screening approaches have been used for medication and DR in the various GI cancer types such as CRC, PC, GC and LC. A deep understanding of cancer progression and treatment has prompted the development of accurate *in vitro* tumor models that better represent the physiological features of the tumor microenvironment. Consequently, recent *in vitro* tumor models have become increasingly complex, extending beyond monitoring primary cell behaviors such as proliferation, invasion and cytotoxicity. These advanced *in vitro* models recapitulate important metastasis steps including angiogenesis, extracellular matrix remodeling and tumor cell metabolism reprogramming and dormancy (Wu and Swartz, 2014).

##### 4.3.1.1 Two-dimensional and three-dimensional cell cultures

Cell culture plates and Transwell-based models are widely employed to assess viability, apoptosis, intra/extravasation and matrix remodeling of cancer cells. Two-dimensional (2D) models have been extensively used for drug screening and drug repurposing (Hulkower and Herber, 2011; Yu et al., 2020; Tomi-Andrino et al., 2022). In recent years, three-dimensional (3D) cell culture methods have become popular as they replicate *in vivo* microenvironmental providing data with greater predictive value for clinical outcomes. These authentic 3D cell culture models using human cells can overcome the limitations of mice models, which in addition to their high cost and ethical implications, are not always capable of accurately mimicking human illnesses or capturing medication side effects such as liver damage (Sivaraman et al., 2005). Moreover, by simulating the cell culture environment, 3D cell cultures can encourage specific cell activity, allowing drug discovery to target cell behavior with greater precision, such as enhancing cellular motility, promoting epithelial cell proliferation and differentiation, inducing cell dormancy, supporting of stem cell-like characteristics or mimicking desired microenvironment, like metastatic niches (Valastyan and Weinberg, 2011). According to their importance and applications in cancer research or DR studies, 3D cell culture models can be categorized into two major groups: Spheroids and Organoids.

##### 4.3.1.2 Spheroids

Spheroids are cell aggressions that grow in suspension or are embedded in a three-dimensional matrix using three-dimensional culture methods. Cancer cell spheroids represent avascular tumor nodules, also known as micro-metastases and are widely used for drug screening despite being more expensive and time-consuming than 2D cell cultures. Spheroids also recapitulate interaction between cells and matrix in the tumor microenvironment and their size-dependent structure includes a necrotic central nucleus, resembling tumors with poor angiogenesis. Moreover, tumor spheroids offer valuable insights into how tumors respond to candidate drugs and combination therapies which reduces the need for animal testing and providing a more realistic representation of the tumor microenvironment (El Harane et al., 2023).

##### 4.3.1.3 Organoids

Organoids are novel *ex vivo* tumor models composed of self-organized three-dimensional multicellular tissue cultures derived from stem cells, primary tissue specimens or cancer cell lines. These models are capable of mimicking the *in vivo* organ (Simian and Bissell, 2017). Patient-derived cancer organoids provide a promising opportunity to predict drug efficiency and treatment response in GI cancers. Furthermore, incorporating 3D cell culture technology with primary patient-derived cancer cells, molecular characterization data, or the establishment of GI organoid banks representing molecular tumor subtypes could lead to preclinical assessment of personalized drug targets to improve treatment outcomes by reducing side effects in cancer therapy (Perrone and Zilbauer, 2021).

The first CRC organoid biobank was established in 2015 for drug screening, enabling the study of gene-drug interactions to recognize potential treatment response biomarkers and understand the molecular basis of drug response (van de Wetering et al., 2015). Notably, numerous studies have reported a significant overlap between esophageal adenocarcinoma organoids and tumor response to standard chemotherapy (Donohoe and Reynolds, 2017; Li et al., 2018) and a similar approach yielded comparable outcomes using GC organoids (Verduin et al., 2021). These results highlight the benefits and advantages of 3D *ex vivo* models in DR and repositioned drug efficiency assessments.

#### 4.3.2 *In vivo* studies of drug repurposing

Animal models offer a powerful tool to simulate physiological conditions and complicated interactions, as well as the responses of reactions of different cell types and tissues to chemicals (Ruggeri et al., 2014). During the drug development process, *in vivo* testing plays a crucial role as it allows the study of interactions between the drug and both target and non-target cells.

Based on the premise of evolutionarily conserved pathogenetic mechanisms, animal models such as zebrafish, mice, fruit flies, worms and yeast have been used to give a comprehensive understanding of the biological mechanisms underlying the effect of drug administration. Xenograft models and genetically engineered mouse cancer models were among the *in vivo* models used in drug repurposing (Freires et al., 2017).

##### 4.3.2.1 Xenograft tumors

The human tumor xenograft is a widely used *in vivo* model where human cancer cells are transferred into immunocompromised (nude) mice through either ectopic or orthotopic implantation. Although the cell line-derived xenograft (CDX) model is considered as the gold-standard model for cancer research and investigation of anti-tumor therapies, patient-derived xenograft (PDX) tumors can also be used for this purpose (Tentler et al., 2012).

Xenograft tumors are highly used as animal models in GI cancer and DR research (Onaciu et al., 2020). *In situ* GI cancer can be induced by locally injecting of cell lines or implantation of tumor cells, while the metastatic models are established by injection of the tumor cells through the tail vein or into the specific organ (Morton and Houghton, 2007; Tentler et al., 2012). For decades, athymic nude mice and mouse xenograft models using human tumor cell lines have been used to study tumor progression factors. In the DR approaches, the use of xenograft tumors can be very helpful in assessing the accuracy of *in vitro* study results and the effectiveness of drugs on cancer cells in safe doses under *in vivo* situations. Doxycycline, a tetracycline-class antibiotic commonly used to treat bacterial and parasitic infections, has demonstrated to reduce tumor growth by ∼80% in pancreatic tumor xenografts (Son et al., 2009; Liu H. et al., 2020).

##### 4.3.2.2 Genetically engineered mouse models

The Genetically Engineered Mouse Model (GEMM) is another animal model for studying human cancer and conducting preclinical study of repurposed drugs to target special gene-derived GI tumors. Genetic technologies have been recently applied by an increasing number of studies to introduce oncogenes into mouse embryonic or somatic cells through tissue-specific promoters targeting the GI tract and inducing GI cancers (Kersten et al., 2017). Transgenic, gene knock-in, and gene knock-out techniques are used to modify the genetic sequence of these mice in order to transfer, mutate, delete or overexpress one or more genes associated with transformation or malignancy. For instance, transgenic mice overexpressing *KRAS* mutant genes can mimic pancreatic tumorigenesis. Indeed, while general single-gene modified models may not fully represent the entire process of GI tumorigenesis, it has been discovered that physiological levels of *KRAS* G12D induce ductal lesions that serve as putative precursors to invasive PC (Westphalen and Olive, 2012). Additional genetic modifications, such as *P53* mutation, can promote tumorigenesis and metastasis (Walrath et al., 2010). Another gene that can be engineered to produce GI cancer GEMM is carbonic anhydrase, present in the basolateral membranes of gastrointestinal epithelial cells and Its overexpression has been reported in many carcinomas including GC. Mice with null mutations of the *Car9* gene develop gastric hyperplasia in glandular epithelium after 1 month (Gut et al., 2002). Additionally, ApcMin/Pten−/− mice are developed to study CRC (Stastna et al., 2019).

GEMM animals can be used to study the impact of a specific gene on tumorigenesis, or to investigate whether a *de novo*/repositioned drug acts through predicted pathways or genes to control the disease (Richmond and Su, 2008). Kobayashi et al. (2017) used mogp-TAg transgenic mice for DR to target the mevalonate pathway as a key tumorigenesis pathway in ovarian cancer (Kobayashi et al., 2017). Furthermore, the effect of metformin cancer initiation and progression suppression was studied using transgenic KPC mice (Chen K. et al., 2017). This animal model was also employed to evaluate the efficiency of repurposed histone deacetylase (HDAC) and mammalian target of rapamycin (mTOR) inhibitors on PC treatment (Biermann et al., 2022). Transgenic animals can be used to evaluate safe, first-in-human (FIH) doses during preclinical studies in drug development. For example, human-CYP3A4-expressing transgenic (Cyp3aXAV) mouse serve as practical model to evaluate the safe dosage and efficiency of CYP3A4-metabolized small-molecule drugs (Damoiseaux et al., 2022). The application of GEMM animal models can prove advantageous in specialized DR studies and allows researchers to focus on specific molecular pathways.

##### 4.3.2.3 Chemically induced gastrointestinal tumors

Chemical agents can be used to induce GI cancers in mice, potentially leading to mutations in relevant human cancer genes. The most well-established chemically induced GC model is produced by the administration of N-Methyl-N-nitrosourea (MNU) (Tomita et al., 2011; Ji et al., 2020; Rabben et al., 2021b). MNU is an N-nitroso compound mostly generated by anaerobic gut bacteria in the presence of nitrates and nitrites (Sobko et al., 2005; Zhuang et al., 2017). Furthermore, 1,2-dimethylhydrazine (DMH) and its metabolite, azoxymethane (AOM), are the most commonly used chemical compounds and carcinogens to induce CRC in mice. It has also shown that intraperitoneal injection of azaserine in rats induces metastatic pancreatic acinar cell carcinoma, although 10% of animals develop tumors in other organs (Rosenberg et al., 2009). Another method to produce a chemically induced PC model is through topical application of benzopyrene which induces adenocarcinoma (Kong et al., 2020). This group of animal models is widely employed in DR studies for GI cancers. Kannen et al. (2012) used C57BL/6 mice exposed to the carcinogen N-methyl-N′-nitro-N-nitrosoguanidine (MNNG) as a colonic carcinogen mouse model to evaluate the antiproliferative effect of fluoxetine, an antidepressant medicine, on colon cancer (Kannen et al., 2012). In a similar study, the inhibitory effects of Liuwei Dihuang Pill (LDP) were investigated on MNU-induced gastric tumorigenesis in diabetic mice (Zhuang et al., 2017).

## 5 Repurposed drugs for gastrointestinal cancers treatment

### 5.1 Colorectal cancer

Researchers in the field of Gl cancer treatment are exploring innovative therapeutic strategies through drug repurposing, with an extensive focus on leveraging existing medications for the management of CRC. Among the studies of significance, Antona et al. (2022) have recently showed that spiperone, an approved drug for schizophrenia treatment, triggers apoptosis in CRC cells by activating phospholipase C, disrupting intracellular calcium balance, inducing irreversible endoplasmic reticulum stress, causing lipid metabolism changes, and damaging the Golgi apparatus (Antona et al., 2022). Furthermore, ten small molecules/drugs were identified via bioinformatic techniques for treating CRC, specifically targeting the upregulated Tissue Inhibitor of Matrix Metalloproteinases-1 (*TIMP1*) gene; these included established agents like formaldehyde and paclitaxel, as well as promising new drug candidates (Leng et al., 2022). Another study discovered that ebselen effectively inhibits Autophagy related protease 4B (*ATG4B*) through oxidative modification. This was based on the FDA-approved drug library, using Fluorescence Resonance Energy Transfer (FRET)-based high-throughput screening and gel-based analysis. The study showcased the potential of ebselen as an anti-CRC agent by influencing autophagy and tumor suppression (Xie et al., 2022). Mao et al. (2023) developed an efficient method for identifying repurposed drugs for CRC using organoid-based screening and computational drug prediction. Out of 335 tested drugs, 34 showed anti-CRC effects, with distinct transcriptome patterns including differentiation induction, growth inhibition, metabolism inhibition, immune response promotion, and cell cycle inhibition. Validation in patient-derived organoid-based xenograft (PDOX) systems demonstrated the anticancer effectiveness of drugs like fedratinib, trametinib, and bortezomib. (Mao et al., 2023). It has been revealed that the antifungal agent oxiconazole induces anti-tumor effects in CRC cells by inhibiting autophagy through downregulation of peroxiredoxin-2 (*PRDX2*), leading to growth suppression, and suggests its potential therapeutic use in combination with oxaliplatin for CRC treatment (Shi J. et al., 2022). Moreover, Dhakal et al. (2022) investigated the cytotoxic effects of the anti-anginal drug perhexiline and its enantiomers on CRC cells and demonstrated their ability to induce apoptosis and reduce cell viability in both monolayers and spheroids, as well as patient-derived organoids (Dhakal et al., 2022). Liñares-Blanco et al. (2020) proposed abemaciclib, an inhibitor of the CDK4/6 protein, as a promising option for the treatment of colon cancer (Liñares-Blanco et al., 2020). Furthermore, mebendazole, an antihelminthic medication used to treat gut worm infections, exhibited a cytotoxic effect on the RKO and HCT-116 colon cancer cell lines. Mebendazole was evaluated against a panel of kinases to determine the mechanism of its cytotoxic effect, which indicated significant inhibitory action against Abl and BRAF proteins. Additionally, in a case study of a patient with resistant metastatic colon cancer, twice-daily therapy with the normal antihelminthic dose of mebendazole led to a substantial reduction in metastasis (Nygren et al., 2013). A comprehensive investigation introduced several promising repurposing drugs (crizotinib, arsenic trioxide, vorinostat, dasatinib, estramustine, and tamibarotene) for CRC by prioritizing candidate genes obtained from the GWAS data (Zhang et al., 2015). Zhao P et al. (2022) highlighted the integration of metabolomics and transcriptomics as a powerful approach to gain insights into the antitumor mechanism of tadalafil, a phosphodiesterase type 5 (PDE5) inhibitor, in patients with CRC (Zhao et al., 2022). The anti-cancer effect of niclosamide was confirmed in nonobese diabetic/severe combined immunodeficiency (NOD/SCID) mice implanted with human CRC xenografts from patients with metastatic CRC and remarkable results were obtained with a non-lethal dose of 200 mg/kg (Osada et al., 2011). Tioconazole, originally used to treat vaginal yeast infections, was reported to enhance chemotherapy efficacy in colorectal tumor xenografts (Liu et al., 2018). Table 1 provides a comprehensive summary of various repurposed drugs in CRC.

### 5.2 Pancreatic cancer

In the field of PC treatment research, recent developments in DR have yielded promising strategies and novel candidates for therapeutic intervention. Pham et al. (2022) have recently introduced a deep learning framework that employs various genome-wide chemical-induced gene expression datasets to predict gene rankings in expression profiles induced by *de novo* chemicals, based on their chemical structures. They used this model for DR to identify potential treatments for PC from all existing drugs in DrugBank, and proposed candidates including dipyridamole, AZD-8055, linagliptin, and preladenant, which were subsequently validated *in vitro* (Pham et al., 2022). A recent study established a biobank of over 30 genetically distinct human pancreatic ductal adenocarcinoma (PDAC) organoid lines, demonstrating their correlation with the molecular and phenotypic heterogeneity observed in primary PDAC tissue and *in vivo* drug responses. Using a fully automated screening platform, this study conducted a DR analysis covering 1,172 FDA-approved drugs. Among the *in vivo* validated hits were several drugs currently approved for non-cancer indications, including emetine and ouabain. These drugs were found to specifically target PDAC organoids by disrupting their response to hypoxia. Notably, a dose of 0.56 mg/kg/d of ouabain significantly reduced PDAC xenograft growth in mice (Hirt et al., 2022). Mugiyanto et al. (2022) used genomic data from the cBio Cancer Genomics Portal to identify PC-associated and drug target genes. Through functional annotations, they prioritized 318 PC risk genes, of which 216 were druggable according to DrugBank. The Connectivity Map (CMap) Touchstone analysis revealed 13 potential PC drugs, including midostaurin and fulvestrant, which target *PRKA* and *ESR1* respectively, as promising candidates for PC treatment (Mugiyanto et al., 2022). Another study investigated whether aspirin (ASA) and oseltamivir phosphate (OP) treatment could enhance PC cell sensitivity to gemcitabine-induced cytotoxicity and hinder chemoresistance development. The combination of ASA and OP with gemcitabine significantly disrupted PC cell viability, clonogenicity, ECM protein expression, migration, and induced apoptosis in MiaPaCa-2 and PANC-1 cells (Qorri et al., 2022). Several studies have recently identified Meflin, a glycosylphosphatidylinositol-anchored membrane molecule, as a functional maker of cancer-restraining CAFs (rCAFs) in PDAC (Kobayashi et al., 2019, 2021; Mizutani et al., 2019; Takahashi et al., 2021). Lida et al. conducted a screening of nuclear receptor ligands and identified Am580, a synthetic retinoid and RARα-selective agonist, as a compound that upregulates Meflin expression in both human pancreatic stellate cells (PSCs) and mouse mesenchymal stem cells (MSCs). Furthermore, Increasing Meflin enhances tumor sensitivity to chemotherapy in a PDAC xenograft model (Iida et al., 2022). The phase I and II clinical trials are conducting to investigate the efficacy of a combination of AM80 and gemcitabine and nab-paclitaxel in patients with advanced PC (Mizutani et al., 2022). Molecular docking technique confirmed that ZINC000001612996, ZINC000052955754, ZINC000003978005, and ZINC000006716957 could potentially act as small molecule drugs and co-ligands for *TRPC3* and *TRPC7*, both of which are part of the Transient Receptor Potential Channels (TRPs)-related gene signature in PDAC (Shi W. et al., 2022). Chen et al. (2020) reported dose-dependent negative effects of albendazole, an anthelmintic drug, on proliferation, migration and viability of the human PC cell lines SW1990 and PANC-1. The cytotoxicity of albendazole was further confirmed *in vivo* using a nude mouse xenograft model, showing a significant reduction in tumor growth (Chen H. et al., 2020). Table 2 presents details on various repurposed drugs for PC.

### 5.3 Liver cancer

Recently, several innovative transcriptomics-based DR methods were employed to uncover novel therapeutic candidates and combinations for the treatment of hepatocellular carcinoma (HCC or LC). Regan-Fendt K et al. (2020) showed that fostamatinib and dasatinib could be effective for sorafenib-resistant HCC (Regan-Fendt et al., 2020). Additionally, an mRNA expression profile-based DR method revealed that *TOP2A* has consistently unfavorable association with HCC patient survival and successfully repositioned withaferin-a (WFA) and mitoxantrone (MTX) through molecular docking studies as potential inhibitors of HepG2 cell proliferation for HCC treatment (Yuan et al., 2022). Tan et al. (2023) have screened a compound library containing 419 FDA-approved drugs and discovered that desloratadine, an antiallergic drug, inhibits proliferation in HCC cell lines as well as CDX, patient-derived organoid (PDO) and PDX. The study also identified N-myristoyl transferase 1 (NMT1) as its target, linking high *NMT1* and *VILIP3* expression to advanced HCC stages and poor survival (Tan et al., 2023). Furthermore, the combined effects of sorafenib, raloxifene, and loratadine on LC cells were assessed, finding that these two- or three-drug combinations significantly reduced metabolic activity, increased apoptosis, and decreased colony formation compared to single-drug treatments, suggesting the potential of the triple combination as a promising approach for LC treatment (Villarruel-Melquiades et al., 2023). We have summarized several repurposed drugs for HCC in Table 3.

### 5.4 Gastric cancer

Zhang et al. (2022) identified 6-Thioguanine (6-TG) as a potential therapeutic agent for GC by inducing ferroptosis through inactivation of system xc^−^, inhibition of glutathione (GSH) production, downregulation of GPX4, and elevation of lipid reactive oxygen species (ROS) levels in MGC-803 and AGS cell lines, with *in vivo* data supporting its anti-tumor activity (Zhang et al., 2022a). Furthermore, it has been shown that HC-056456, a CatSper channel blocker, as a novel ferroptosis-inducing compound inhibits GC cell growth by reducing GSH through the p53/SLC7A11 pathway, leading to increased Fe2+ and lipid peroxides *in vitro* and *in vivo* (Zhang et al., 2022b). In a comprehensive bioinformatic analysis of highly differentially expressed genes in GC, Hossain et al. (2023) found that *CDH2*, *COL4A1*, and *COL5A* are associated with the survival of GC patients. They also identified docetaxel, lanreotide, venetoclax, temsirolimus, and nilotinib as the top six candidate drugs, targeting aforementioned proteins, for the treatment of GC patients (Hossain et al., 2023). Nitazoxanide also yielded favorable outcomes, as it exhibited activity across GC cell lines tested (Ribeiro et al., 2023). In addition, Rabben et al. (2021a) computationally predicted the repositioning of ivermectin for the treatment of GC based on gene expression profiles of both human and mouse models of GC. They further validated their *in silico* prediction used human GC cell lines MKN74 and KATO-III *in vitro.* Transgenic insulin–gastrin (INS-GAS) mice were employed for experimental validation of ivermectin in GC treatment (Rabben et al., 2021a). Furthermore, *in vivo* and *in vitro* anti-tumor and growth suppression effects of ivermectin were demonstrated on GC, showing that ivermectin suppressed MKN1 cells growth through yes-associated protein 1 (YAP1) downregulation (Nambara et al., 2017). To identify drugs capable of inhibiting the DNA-binding activity of the *helicobacter pylori* transcription factor HP104, a combination of computational and *in vitro* methods led to the discovery of three promising drugs, including temoporfin, trientine, and tetraethylenepentamine, for potential antibacterial applications (Antoniciello et al., 2022). Table 4 provides a summary of available repurposed drugs in GC.

### 5.5 Other examples of drug repurposing in gastrointestinal cancers

Surveillance for individuals at risk of GI cancers is essential for early diagnosis and prognosis improvement, and in choosing long-term chemoprotective drugs, approved molecules with well-known long-term effects are preferred. Aspirin (acetylsalicylic acid), introduced at the end of the 19th century, has been proposed for various diseases, such as cardiovascular diseases, strokes (Brighton et al., 2012; Dimitriadis et al., 2022; Gdovinova et al., 2022) and the chronic treatment of Fabry Disease (Monticelli et al., 2022). Specifically, aspirin has been shown to prevent CRC (Baron et al., 2003; Benamouzig et al., 2012; Guirguis-Blake et al., 2022) and PC (Streicher et al., 2014). Other non-steroid anti-inflammatory molecules like celecoxib (Arber et al., 2006; Bertagnolli et al., 2006) and sulindac (Long et al., 2020) have also been suggested for CRC.

Recent studies have highlighted the promising potential of repurposing non-oncology drugs for future cancer therapy. Examples include anticoagulant agents (warfarin (Rebelo et al., 2021) and dalteparin (Agnelli et al., 2022)), anti-fungi (itraconazole (Shen et al., 2021)), antidiabetic drugs (metformin (Cunha Júnior et al., 2021) and linagliptin (Li Y. et al., 2020)), antiparasitic (ivermectin (Nambara et al., 2017)), anthelminthic (parbendazole (Son et al., 2020)), antibiotics (nitroxoline (Mitrović and Kos, 2019), doxycycline (Ghasemi and Ghasemi, 2022), azithromycin (Qiao et al., 2018) and tigecycline (ElHefnawi et al., 2022)).

Brefeldin A, originally used as a macrolide antibiotic, has shown significant induction of autophagy in CRC cells both *in vitro* and *in vivo* (Bei et al., 2022). It functions by provoking endoplasmic reticulum stress (ER-stress) and upregulating Bip which decreases Akt phosphorylation through increased Bip/Akt interaction leading to autophagy induction in CRC cells (Zhou L. et al., 2019). In addition, antifungal drug ketoconazole has been reported to induce PINK1/Parkin-mediated mitophagy and accelerate apoptosis in HCC cells via COX-2 downregulation (Chen Y. et al., 2019).

Genistein, originally prescribed for reducing symptoms of menopause, osteoporosis, and obesity, has shown promising effects in cancer therapy. Genistein has been reported to inhibit proliferation, induce apoptosis and cell cycle arrest in G2 by inhibiting the Wnt/β-catenin signaling pathway in CRC cells (Oliveira et al., 2022). It also promotes apoptosis in HT29 CRC cells by modulating the caspase-3 and p38 MAPK signaling pathways (Shafiee et al., 2016). Furthermore, genistein inhibits glycolysis and induces mitochondrial apoptosis through downregulating of HIF-1α which leads to GLUT1 and HK2 inactivation and apoptosis induction in drug-resistant HCC cells (Li J. et al., 2017). Genistein modulates telomerase activity and reduces tumorigenesis by *hTERT* downregulation as well as modulation of *Gli1* gene expression to weaken cancer stem-like properties in GC cells (Jian-Hui et al., 2016). Genistein, which shows promise as an anticancer drug candidate, is currently in phase II of clinical trials (Cao et al., 2022; Chu et al., 2023).

Metformin is another successful repositioned drug for GI cancers which is currently in phase II of the clinical trial. Metformin reduces cell survival and tumorigenesis by lowering serum insulin levels and downregulation of IGF-1 (Sarfstein et al., 2013). Additionally, it induces G1-arrest via AMPK activation and cyclin D1 downregulation (Wang Y. et al., 2018), inhibits proliferation through mTOR signaling pathway regulation regardless of AMPK dependency (Demaré et al., 2021). It has been shown that metformin inhibits the progression of GC through the inhabitation of HIF1α/PKM2 signaling (Chen G. et al., 2015). Furthermore, it inactivates RAS/ERK and AKT/mTOR signaling pathways and reduces proliferation in KRAS-derived tumors. Metformin has been reported to selectively inhibit *KRAS*-driven metastatic CRC by silencing MATE1 (Xie et al., 2020).

Several Studies have demonstrated that the combination of repurposed drugs with cytotoxic drugs, radiotherapy or even the combination of multiple repositioned drugs can exhibit synergistic antitumor effects. A recent meta-analysis by Heer et al. (2022) revealed that aspirin when co-administered with sulindac and difluoromethylornithine (DFMO), an inhibitor of ornithine decarboxylase used to treat facial hirsutism, showed significantly more effective results in protecting against CRC adenomas (Heer et al., 2022). The combination of bortezomib and chloroquine has been shown to suppress proliferation and induce apoptosis in human liver tumors, whether orthotopically or subcutaneously xenografted in mice (Hui et al., 2012). In clinical research, combining nelfinavir with a short course of hypofractionated radiotherapy (SCHRT) showed increased sensitivity of CRC tumors to radiotherapy (Meyn et al., 2016). In addition, FOLFIRINOX, a chemotherapy regimen comprising leucovorin calcium (folinic acid), fluorouracil, irinotecan hydrochloride and oxaliplatin, is used for the treatment of advanced PC. In a phase II clinical trial, FOLFIRINOX is combined with losartan as neoadjuvant therapy, followed by chemoradiotherapy, for locally advanced PC (Murphy et al., 2019). Another successful example of DR is a combination of metformin, digoxin and somatostatin, which has shown significant suppression of PC cell proliferation in clinically relevant animal models. Currently, this combination is being evaluated in a clinical trial (Liu S.-H. et al., 2020). Tables 1–4 provide comprehensive information on various drugs that have been successfully repurposed to treat GI cancers and approved for clinical use by the FDA. Furthermore, we have prepared a Supplementary Table S2, which presents a list of repurposed unapproved or withdrawn drugs/natural components targeting GI-related cancers.

## 6 Challenges and future perspectives

Drug repurposing for cancer therapy is widely used to discover new indications for existing compounds; However, only a few repurposed drugs have been formally subjected to the clinical treatment guidelines. Despite advantages such as anticancer pharmacokinetic parameters, acceptable safety and tolerability in humans, there is still a risk of late-phase clinical trial failure due to competition with new drug development. The legal and regulatory barriers such as patents issues and prescription charges must also be addressed (Vickers, 2017; Breckenridge and Jacob, 2019; Pushpakom et al., 2019).

The financial factors significantly influence the clinical development and approval of repurposing drugs, as private sector organizations prioritize higher returns due to intellectual property rights (Vickers, 2017). Most registered clinical trials listed in the Repurposing Drugs in Oncology (ReDo) project are sponsored by Universities or Hospitals (67%), research institutes or non-profit organizations (28%), and only a small percentage by pharmaceutical companies (Pantziarka et al., 2018). Patent considerations for off-patent drugs can pose significant barriers to DR, requiring credible and statistically strong evidence for a new indication and legislative efforts to address this issue (Pushpakom et al., 2019).

Physicians prescribe drugs based on scientific evidence from clinical trials, and both generic and repurposed drugs should be used when suitable. Nevertheless, the pharmaceutical industry can exert substantial investments on drug promotion, physician marketing and consumer advertising. A clear example is thalidomide, originally used as a sedative or antiemetic, which has been repurposed for multiple myeloma. Despite phase III clinical trials showing no survival advantage for the combination of melphalan-prednisone-lenalidomide over melphalan-prednisone-thalidomide (Stewart et al., 2015; Zweegman et al., 2016) lenalidomide became the standard treatment approach, even with a higher estimated cost than thalidomide. Thus, underfunding for clinical trials in oncology and prescribing rejection bias remain significant challenges for cancer drug repurposing.

Another challenge lies in the genetic diversity of individuals and the complex nature of disease (Pritchard et al., 2017). While there are many common pathways involved in cancer development, differences in pathways and genes exist among subgroups and individuals. This genetic diversity results in varying side effects and treatment responses to routine drugs and therapies.

Moreover, current GI cancer therapies may be ineffective for specific patients or cancer types due to inadequate drug targeting or inefficient drug interactions (Apicella et al., 2017). In rectal cancer, personalized DR based on gene expression signatures and reverse drug-induced gene expression profiles has shown promising results. For instance, Carvalho et al. (2021) identified potential topoisomerase II inhibitors like doxorubicin, teniposide, idarubicin, mitoxantrone and epirubicin for CRC therapy, leading to a significant reversal of rectal cancer gene expression signatures (Carvalho et al., 2021). Given the significant differences in drug efficacy among individuals due to gene profiles and tumor heterogeneity, it becomes crucial to focus on DR based on tumor/subject molecular profiles to reduce inefficiencies in cancer treatment (Li and Jones, 2012).

While drug repurposing offers various benefits compared to the conventional *de novo* approach, it may not always lead to success due to lack of efficacy or toxicity issues. Bevacizumab, initially developed to treat CRC, was determined to be a strong candidate to treat other kinds of cancer such as colon, rectal, brain, lung, and kidney through drug repositioning. However, it failed in phase III trials despite positive results (Kim and Oh, 2018). These failed drug candidates in clinical trials still represent an affluent resource for repositioning, as they are well studied pharmacokinetically and clinically. Personalized genomics studies focusing on patient and disease heterogeneities may reveal that many of these failures were tested in inappropriate subject groups, making them practical options for future personalized medicine approaches, particularly for subjects with limited treatment options.

## 7 Conclusion

Drug repurposing is increasingly considered by both academia and the pharmaceutical industry as a cost and time-saving alternative to *de novo* drug development. Repurposing non-oncology drugs in cancer therapy provides a promising therapeutic opportunity, especially for patients with rare cancers, advanced diseases, or chemo-resistant tumors. In the present review, we have explored the potential of DR approaches, with a particular focus on their application in GI cancers. Repurposed drugs can target known pathways and key molecular targets in cancer biology due to their established functional mechanisms. DR has received attention owing to its potential to enhance treatment effectiveness and ability to overwhelm resistance to standard chemotherapy as well as improve therapy outcomes in tumors with limited response to conventional treatments. Additionally, when repurposed drugs are used in combination with routine oncology drugs, they offer a unique opportunity to target multiple pathways and molecular targets in cancer cells, going beyond the scope of traditional chemotherapy drugs and modulating diverse cancer-relevant pathways. However, it should be noted that the interaction of repurposed drugs with standard cancer drugs may pose challenges during the clinical trials.

The repositioning of drugs to treat GI cancers presents an attractive option given the increasing number of new cases, annual deaths, and the challenges in treating certain tumors. This review outlines various DR approaches that can be used to improve the efficiency of existing GI therapies. However, further clinical studies are needed to determine their potential for clinical adoption.

## References

[B2] Acedo P. Fernandes A. Zawacka-Pankau J. (2019). Activation of TAp73 and inhibition of TrxR by Verteporfin for improved cancer therapy in TP53 mutant pancreatic tumors. Future Sci. OA 5, FSO366. 10.4155/fsoa-2018-0082 30820346 PMC6391631

[B3] Agnelli G. Muñoz A. Franco L. Mahé I. Brenner B. Connors J. M. (2022). Apixaban and dalteparin for the treatment of venous thromboembolism in patients with different sites of cancer. Thromb. Haemost. 122, 796–807. 10.1055/s-0041-1735194 34530482

[B4] Ahmed M. Jinks N. Babaei-Jadidi R. Kashfi H. Castellanos-Uribe M. May S. T. (2019). Repurposing antibacterial AM404 as a potential anticancer drug for targeting colorectal cancer stem-like cells. Cancers 12, 106. 10.3390/cancers12010106 31906201 PMC7017077

[B5] Alburquerque-González B. Bernabé-García Á. Bernabé-García M. Ruiz-Sanz J. López-Calderón F. F. Gonnelli L. (2021). The FDA-approved antiviral raltegravir inhibits fascin1-dependent invasion of colorectal tumor cells *in vitro* and *in vivo* . Cancers 13, 861. 10.3390/cancers13040861 33670655 PMC7921938

[B6] Al-Wadei H. A. Al-Wadei M. H. Schuller H. M. (2009). Prevention of pancreatic cancer by the beta-blocker propranolol. Anticancer Drugs 20, 477–482. 10.1097/CAD.0b013e32832bd1e3 19387337 PMC3366433

[B7] Anselmino L. E. Baglioni M. V. Reynoso G. Rozados V. R. Scharovsky O. G. Rico M. J. (2023). Potential effect of chloroquine and propranolol combination to treat colorectal and triple-negative breast cancers. Sci. Rep. 13, 7923–7927. 10.1038/s41598-023-34793-6 37193722 PMC10188563

[B8] Antona A. Varalda M. Roy K. Favero F. Mazzucco E. Zuccalà M. (2022). Dissecting the mechanism of action of spiperone-A candidate for drug repurposing for colorectal cancer. Cancers 14, 776. 10.3390/cancers14030776 35159043 PMC8834219

[B9] Antoniciello F. Roncarati D. Zannoni A. Chiti E. Scarlato V. Chiappori F. (2022). Targeting the essential transcription factor HP1043 of *Helicobacter pylori*: a drug repositioning study. Front. Mol. Biosci. 9, 887564. 10.3389/fmolb.2022.887564 35647033 PMC9135449

[B10] Anwanwan D. Singh S. K. Singh S. Saikam V. Singh R. (2020). Challenges in liver cancer and possible treatment approaches. Biochim. Biophys. Acta Rev. Cancer 1873, 188314. 10.1016/j.bbcan.2019.188314 31682895 PMC6981221

[B11] Apicella M. Corso S. Giordano S. (2017). Targeted therapies for gastric cancer: failures and hopes from clinical trials. Oncotarget 8, 57654–57669. 10.18632/oncotarget.14825 28915702 PMC5593674

[B12] Arber N. Eagle C. J. Spicak J. Rácz I. Dite P. Hajer J. (2006). Celecoxib for the prevention of colorectal adenomatous polyps. N. Engl. J. Med. 355, 885–895. 10.1056/NEJMoa061652 16943401

[B13] Arulanandam C. D. Prathiviraj R. Kaveriyappan G. R. (2021). Repurposing of an antifungal drug against gastrointestinal stromal tumors. bioRxiv. 10.1101/2021.01.15.426618

[B14] Ashburn T. T. Thor K. B. (2004). Drug repositioning: identifying and developing new uses for existing drugs. Nat. Rev. Drug Discov. 3, 673–683. 10.1038/nrd1468 15286734

[B15] Attia Y. M. Tawfiq R. A. Ali A. A. Elmazar M. M. (2017). The FXR agonist, obeticholic acid, suppresses HCC proliferation & metastasis: role of IL-6/STAT3 signalling pathway. Sci. Rep. 7, 12502. 10.1038/s41598-017-12629-4 28970500 PMC5624958

[B16] Au T. H. Wang K. Stenehjem D. Garrido-Laguna I. (2017). Personalized and precision medicine: integrating genomics into treatment decisions in gastrointestinal malignancies. J. Gastrointest. Oncol. 8, 387–404. 10.21037/jgo.2017.01.04 28736627 PMC5506274

[B17] Ayoub B. M. Attia Y. M. Ahmed M. S. (2018). Structural re-positioning, *in silico* molecular modelling, oxidative degradation, and biological screening of linagliptin as adenosine 3 receptor (ADORA3) modulators targeting hepatocellular carcinoma. J. Enzyme Inhib. Med. Chem. 33, 858–866. 10.1080/14756366.2018.1462801 29768061 PMC6010121

[B18] Bago R. Sommer E. Castel P. Crafter C. Bailey F. P. Shpiro N. (2016). The hVps34-SGK3 pathway alleviates sustained PI3K/Akt inhibition by stimulating mTORC1 and tumour growth. EMBO J. 35, 2263. 10.15252/embj.201670010 27798145 PMC5069549

[B19] Bahmad H. F. Demus T. Moubarak M. M. Daher D. Alvarez Moreno J. C. Polit F. (2022). Overcoming drug resistance in advanced prostate cancer by drug repurposing. Med. Sci. (Basel) 10, 15. 10.3390/medsci10010015 35225948 PMC8883996

[B20] Baron J. A. Cole B. F. Sandler R. S. Haile R. W. Ahnen D. Bresalier R. (2003). A randomized trial of aspirin to prevent colorectal adenomas. N. Engl. J. Med. 348, 891–899. 10.1056/NEJMoa021735 12621133

[B21] Batchu R. B. Gruzdyn O. V. Bryant C. S. Qazi A. M. Kumar S. Chamala S. (2014). Ritonavir-mediated induction of apoptosis in pancreatic cancer occurs via the RB/E2F-1 and AKT pathways. Pharmaceuticals 7, 46–57. 10.3390/ph7010046 24451403 PMC3915194

[B22] Baxendale S. van Eeden F. Wilkinson R. (2017). The power of zebrafish in personalised medicine. Adv. Exp. Med. Biol. 1007, 179–197. 10.1007/978-3-319-60733-7_10 28840558

[B23] Bei S. Xu Q. Li F. Wu C. Sun Q. Feng L. (2022). Brefeldin A: a newly identified cell death inducer selectively targets radio-resistant colorectal cancer cells by direct interacting with caspase-3. J. King Saud. Univ. Sci. 34, 101728. 10.1016/j.jksus.2021.101728

[B24] Bejan C. A. Wei W.-Q. Denny J. C. (2015). Assessing the role of a medication-indication resource in the treatment relation extraction from clinical text. J. Am. Med. Inf. Assoc. 22, e162–e176. 10.1136/amiajnl-2014-002954 PMC5901126 25336593

[B25] Benamouzig R. Uzzan B. Deyra J. Martin A. Girard B. Little J. (2012). Prevention by daily soluble aspirin of colorectal adenoma recurrence: 4-year results of the APACC randomised trial. Gut 61, 255–261. 10.1136/gutjnl-2011-300113 21890814

[B26] Bertagnolli M. M. Eagle C. J. Zauber A. G. Redston M. Solomon S. D. Kim K. (2006). Celecoxib for the prevention of sporadic colorectal adenomas. N. Engl. J. Med. 355, 873–884. 10.1056/NEJMoa061355 16943400

[B27] Bertolini F. Sukhatme V. P. Bouche G. (2015). Drug repurposing in oncology--patient and health systems opportunities. Nat. Rev. Clin. Oncol. 12, 732–742. 10.1038/nrclinonc.2015.169 26483297

[B28] Biermann M. Quintero C. Ferguson P. Rajbhandari N. Park D. E. Patel H. (2022). Repurposing HDAC and mTOR inhibitors for pancreatic cancer. J. Clin. Oncol. 40, e16234, 10.1200/jco.2022.40.16_suppl.e16234

[B29] Biller L. H. Schrag D. (2021). Diagnosis and treatment of metastatic colorectal cancer: a review. JAMA 325, 669–685. 10.1001/jama.2021.0106 33591350

[B30] Booth C. M. Karim S. Mackillop W. J. (2019). Real-world data: towards achieving the achievable in cancer care. Nat. Rev. Clin. Oncol. 16, 312–325. 10.1038/s41571-019-0167-7 30700859

[B31] Breckenridge A. Jacob R. (2019). Overcoming the legal and regulatory barriers to drug repurposing. Nat. Rev. Drug Discov. 18, 1–2. 10.1038/nrd.2018.92 29880920

[B32] Brehmer D. Greff Z. Godl K. Blencke S. Kurtenbach A. Weber M. (2005). Cellular targets of gefitinib. Cancer Res. 65, 379–382. 10.1158/0008-5472.379.65.2 15695376

[B33] Brighton T. A. Eikelboom J. W. Mann K. Mister R. Gallus A. Ockelford P. (2012). Low-dose aspirin for preventing recurrent venous thromboembolism. N. Engl. J. Med. 367, 1979–1987. 10.1056/NEJMoa1210384 23121403

[B34] Cao X. Ren K. Song Z. Li D. Quan M. Zheng Y. (2022). [Corrigendum] 7-Difluoromethoxyl-5,4’-di-n-octyl genistein inhibits the stem-like characteristics of gastric cancer stem-like cells and reverses the phenotype of epithelial-mesenchymal transition in gastric cancer cells. Oncol. Rep. 48, 176. 10.3892/or.2022.8391 27279287

[B35] Carter T. A. Wodicka L. M. Shah N. P. Velasco A. M. Fabian M. A. Treiber D. K. (2005). Inhibition of drug-resistant mutants of ABL, KIT, and EGF receptor kinases. Proc. Natl. Acad. Sci. U. S. A. 102, 11011–11016. 10.1073/pnas.0504952102 16046538 PMC1180625

[B36] Carvalho R. F. do Canto L. M. Cury S. S. Frøstrup Hansen T. Jensen L. H. Rogatto S. R. (2021). Drug repositioning based on the reversal of gene expression signatures identifies *TOP2A* as a therapeutic target for rectal cancer. Cancers 13, 5492. 10.3390/cancers13215492 34771654 PMC8583090

[B37] Catalano M. Aprile G. Conca R. Petrioli R. Ramello M. Roviello G. (2022). The impact of age, performance status and comorbidities on nab-paclitaxel plus gemcitabine effectiveness in patients with metastatic pancreatic cancer. Sci. Rep. 12, 8244. 10.1038/s41598-022-12214-4 35581246 PMC9114343

[B38] Cavalla D. Singal C. (2012). Retrospective clinical analysis for drug rescue: for new indications or stratified patient groups. Drug Discov. Today 17, 104–109. 10.1016/j.drudis.2011.09.019 22001144

[B39] Chaiteerakij R. Petersen G. M. Bamlet W. R. Chaffee K. G. Zhen D. B. Burch P. A. (2016). Metformin use and survival of patients with pancreatic cancer: a cautionary lesson. J. Clin. Oncol. 34, 1898–1904. 10.1200/JCO.2015.63.3511 27069086 PMC4966342

[B40] Chakraborty S. Rahman T. (2012). The difficulties in cancer treatment. Ecancermedicalscience 6, ed16. 10.3332/ecancer.2012.ed16 24883085 PMC4024849

[B41] Chantrill L. A. Nagrial A. M. Watson C. Johns A. L. Martyn-Smith M. Simpson S. (2015). Precision medicine for advanced pancreas cancer: the individualized molecular pancreatic cancer therapy (IMPaCT) trial. Clin. Cancer Res. 21, 2029–2037. 10.1158/1078-0432.CCR-15-0426 25896973

[B42] Chao M.-W. Chen T.-H. Huang H.-L. Chang Y.-W. HuangFu W.-C. Lee Y.-C. (2017). Lanatoside C, a cardiac glycoside, acts through protein kinase Cδ to cause apoptosis of human hepatocellular carcinoma cells. Sci. Rep. 7, 46134. 10.1038/srep46134 28387249 PMC5384006

[B43] Chen B. Garmire L. Calvisi D. F. Chua M.-S. Kelley R. K. Chen X. (2020a). Publisher Correction: harnessing big 'omics' data and AI for drug discovery in hepatocellular carcinoma. Nat. Rev. Gastroenterol. Hepatol. 17, 238–251. 10.1038/s41575-020-0288-6 31900465 PMC7401304

[B44] Chen B. Wei W. Ma L. Yang B. Gill R. M. Chua M.-S. (2017a). Computational discovery of niclosamide ethanolamine, a repurposed drug candidate that reduces growth of hepatocellular carcinoma cells *in vitro* and in mice by inhibiting cell division cycle 37 signaling. Gastroenterology 152, 2022–2036. 10.1053/j.gastro.2017.02.039 28284560 PMC5447464

[B45] Chen C.-T. Chen Y.-C. Yamaguchi H. Hung M.-C. (2015a). Carglumic acid promotes apoptosis and suppresses cancer cell proliferation *in vitro* and *in vivo* . Am. J. Cancer Res. 5, 3560–3569. 26885446 PMC4731631

[B46] Chen G. Feng W. Zhang S. Bian K. Yang Y. Fang C. (2015b). Metformin inhibits gastric cancer via the inhibition of HIF1α/PKM2 signaling. Am. J. Cancer Res. 5, 1423–1434. 26101707 PMC4473320

[B47] Chen H. Weng Z. Xu C. (2020b). Albendazole suppresses cell proliferation and migration and induces apoptosis in human pancreatic cancer cells. Anticancer Drugs 31, 431–439. 10.1097/CAD.0000000000000914 32044795

[B48] Chen H.-N. Chen Y. Zhou Z.-G. Wei Y. Huang C. (2019a). A novel role for ketoconazole in hepatocellular carcinoma treatment: linking PTGS2 to mitophagy machinery. Autophagy 15, 733–734. 10.1080/15548627.2019.1569934 30653402 PMC6526862

[B49] Chen K. Cheng L. Qian W. Jiang Z. Sun L. Zhao Y. (2018). Itraconazole inhibits invasion and migration of pancreatic cancer cells by suppressing TGF-β/SMAD2/3 signaling. Oncol. Rep. 39, 1573–1582. 10.3892/or.2018.6281 29484419

[B50] Chen K. Qian W. Jiang Z. Cheng L. Li J. Sun L. (2017b). Metformin suppresses cancer initiation and progression in genetic mouse models of pancreatic cancer. Mol. Cancer 16, 131. 10.1186/s12943-017-0701-0 28738823 PMC5525317

[B51] Chen V. C.-H. Lin C.-F. Hsieh Y.-H. Liang H.-Y. Huang K.-Y. Chiu W.-C. (2017c). Hepatocellular carcinoma and antidepressants: a nationwide population-based study. Oncotarget 8, 30464–30470. 10.18632/oncotarget.12826 27783998 PMC5444756

[B52] Chen W. Chen F. Gong M. Ye L. Weng D. Jin Z. (2023). Fenofibrate suppresses the progression of hepatoma by downregulating osteopontin through inhibiting the PI3K/AKT/Twist pathway. Naunyn. Schmiedeb. Arch. Pharmacol., 1–11. 10.1007/s00210-023-02604-4 PMC10791796 37566308

[B53] Chen Y. Chen H.-N. Wang K. Zhang L. Huang Z. Liu J. (2019b). Ketoconazole exacerbates mitophagy to induce apoptosis by downregulating cyclooxygenase-2 in hepatocellular carcinoma. J. Hepatol. 70, 66–77. 10.1016/j.jhep.2018.09.022 30287340

[B54] Cheng F. Lu W. Liu C. Fang J. Hou Y. Handy D. E. (2019). A genome-wide positioning systems network algorithm for *in silico* drug repurposing. Nat. Commun. 10, 3476. 10.1038/s41467-019-10744-6 31375661 PMC6677722

[B55] Cheng X. Zhao W. Zhu M. Wang B. Wang X. Yang X. (2021). Drug repurposing for cancer treatment through global propagation with a greedy algorithm in a multilayer network. Cancer Biol. Med. 19, 74–89. 10.20892/j.issn.2095-3941.2020.0218 33893730 PMC8762999

[B56] Chong C. R. Sullivan D. J. Jr (2007). New uses for old drugs. Nature 448, 645–646. 10.1038/448645a 17687303

[B57] Chu Y.-D. Chen C.-W. Lai M.-W. Lim S.-N. Lin W.-R. (2023). Bioenergetic alteration in gastrointestinal cancers: the good, the bad and the ugly. World J. Gastroenterol. 29, 4499–4527. 10.3748/wjg.v29.i29.4499 37621758 PMC10445009

[B58] Ciociola A. A. Cohen L. B. Kulkarni P. (2014). How drugs are developed and approved by the FDA: current process and future directions. Am. J. Gastroenterol. 109, 620–623. 10.1038/ajg.2013.407 24796999

[B59] Clements W. K. Traver D. (2012). Fish pharming: zebrafish antileukemia screening. Blood 119, 5614–5615. 10.1182/blood-2012-04-425249 22700692

[B60] Cong F. Cheung A. K. Huang S.-M. A. (2012). Chemical genetics-based target identification in drug discovery. Annu. Rev. Pharmacol. Toxicol. 52, 57–78. 10.1146/annurev-pharmtox-010611-134639 21819237

[B61] Cousin M. A. Ebbert J. O. Wiinamaki A. R. Urban M. D. Argue D. P. Ekker S. C. (2014). Larval zebrafish model for FDA-approved drug repositioning for tobacco dependence treatment. PLoS One 9, e90467. 10.1371/journal.pone.0090467 24658307 PMC3962344

[B62] Crawford S. (2014). Anti-inflammatory/antioxidant use in long-term maintenance cancer therapy: a new therapeutic approach to disease progression and recurrence. Ther. Adv. Med. Oncol. 6, 52–68. 10.1177/1758834014521111 24587831 PMC3932057

[B63] Cunha Júnior A. D. Bragagnoli A. C. Costa F. O. Carvalheira J. B. C. (2021). Repurposing metformin for the treatment of gastrointestinal cancer. World J. Gastroenterol. 27, 1883–1904. 10.3748/wjg.v27.i17.1883 34007128 PMC8108031

[B64] Dakshanamurthy S. Issa N. T. Assefnia S. Seshasayee A. Peters O. J. Madhavan S. (2012). Predicting new indications for approved drugs using a proteochemometric method. J. Med. Chem. 55, 6832–6848. 10.1021/jm300576q 22780961 PMC3419493

[B65] Dalwadi S. M. Hunt A. Bonnen M. D. Ghebre Y. T. (2023). Computational approaches for drug repurposing in oncology: untapped opportunity for high value innovation. Front. Oncol. 13, 1198284. 10.3389/fonc.2023.1198284 37274281 PMC10233043

[B66] Damoiseaux D. Li W. Martínez-Chávez A. Beijnen J. H. Schinkel A. H. Huitema A. D. R. (2022). Predictiveness of the human-CYP3A4-transgenic mouse model (Cyp3aXAV) for human drug exposure of CYP3A4-metabolized drugs. Pharmaceuticals 15, 860. 10.3390/ph15070860 35890158 PMC9322370

[B67] Dehnavi S. Kiani A. Sadeghi M. Biregani A. F. Banach M. Atkin S. L. (2021). Targeting AMPK by statins: a potential therapeutic approach. Drugs 81, 923–933. 10.1007/s40265-021-01510-4 33939118 PMC8144155

[B68] Demaré S. Kothari A. Calcutt N. A. Fernyhough P. (2021). Metformin as a potential therapeutic for neurological disease: mobilizing AMPK to repair the nervous system. Expert Rev. Neurother. 21, 45–63. 10.1080/14737175.2021.1847645 33161784 PMC9482886

[B69] Dhakal B. Li C. M. Y. Li R. Yeo K. Wright J. A. Gieniec K. A. (2022). The antianginal drug perhexiline displays cytotoxicity against colorectal cancer cells *in vitro*: a potential for drug repurposing. Cancers 14, 1043. 10.3390/cancers14041043 35205791 PMC8869789

[B70] Díaz-Carballo D. Acikelli A. H. Klein J. Jastrow H. Dammann P. Wyganowski T. (2015). Therapeutic potential of antiviral drugs targeting chemorefractory colorectal adenocarcinoma cells overexpressing endogenous retroviral elements. J. Exp. Clin. Cancer Res. 34, 81. 10.1186/s13046-015-0199-5 26260344 PMC4542094

[B71] Dimitriadis K. Lazarou E. Tsioufis P. Soulaidopoulos S. Tsioufis K. (2022). Aspirin for primary prevention of cardiovascular diseases: “WALTZ” with the evidence. Curr. Cardiol. Rep. 24, 1139–1147. 10.1007/s11886-022-01740-2 35857202 PMC9297059

[B72] Diop-Frimpong B. Chauhan V. P. Krane S. Boucher Y. Jain R. K. (2011). Losartan inhibits collagen I synthesis and improves the distribution and efficacy of nanotherapeutics in tumors. Proc. Natl. Acad. Sci. U. S. A. 108, 2909–2914. 10.1073/pnas.1018892108 21282607 PMC3041115

[B73] Donohoe C. L. Reynolds J. V. (2017). Neoadjuvant treatment of locally advanced esophageal and junctional cancer: the evidence-base, current key questions and clinical trials. J. Thorac. Dis. 9, S697–S704. 10.21037/jtd.2017.03.159 28815065 PMC5538972

[B74] Dou C. Mo H. Chen T. Liu J. Zeng Y. Li S. (2021). ZMYND8 promotes the growth and metastasis of hepatocellular carcinoma by promoting HK2-mediated glycolysis. Pathol. Res. Pract. 219, 153345. 10.1016/j.prp.2021.153345 33517164

[B75] Du D. Liu C. Qin M. Zhang X. Xi T. Yuan S. (2022). Metabolic dysregulation and emerging therapeutical targets for hepatocellular carcinoma. Acta Pharm. Sin. B 12, 558–580. 10.1016/j.apsb.2021.09.019 35256934 PMC8897153

[B76] Ducreux M. Abou-Alfa G. K. Bekaii-Saab T. Berlin J. Cervantes A. de Baere T. (2023). The management of hepatocellular carcinoma. Current expert opinion and recommendations derived from the 24th ESMO/World Congress on Gastrointestinal Cancer, Barcelona. ESMO Open 8, 101567. 10.1016/j.esmoop.2023.101567 37263081 PMC10245111

[B77] Dudley J. T. Deshpande T. Butte A. J. (2011). Exploiting drug-disease relationships for computational drug repositioning. Brief. Bioinform. 12, 303–311. 10.1093/bib/bbr013 21690101 PMC3137933

[B78] Eichler H.-G. Bloechl-Daum B. Broich K. Kyrle P. A. Oderkirk J. Rasi G. (2019). Data rich, information poor: can we use electronic health records to create a learning healthcare system for pharmaceuticals? Clin. Pharmacol. Ther. 105, 912–922. 10.1002/cpt.1226 30178490 PMC6587701

[B79] El-Hachem N. Gendoo D. M. A. Ghoraie L. S. Safikhani Z. Smirnov P. Chung C. (2017). Integrative cancer pharmacogenomics to infer large-scale drug taxonomy. Cancer Res. 77, 3057–3069. 10.1158/0008-5472.CAN-17-0096 28314784

[B80] El Harane S. Zidi B. El Harane N. Krause K.-H. Matthes T. Preynat-Seauve O. (2023). Cancer spheroids and organoids as novel tools for research and therapy: state of the art and challenges to guide precision medicine. Cells 12. 10.3390/cells12071001 PMC10093533 37048073

[B81] ElHefnawi M. Jo E. Tolba M. M. Fares M. Yang J. Shahbaaz M. (2022). Drug repurposing through virtual screening and *in vitro* validation identifies tigecycline as a novel putative HCV polymerase inhibitor. Virology 570, 9–17. 10.1016/j.virol.2022.02.006 35338891

[B82] Emamzaden F. N. Word B. Hammons G. Lyn-Cook B. D. (2022). Abstract 1631: anti-cancer effects of vorinostat on 3D cultured pancreatic cancer cells. Cancer Res. 82, 1631. 10.1158/1538-7445.am2022-1631

[B83] Falcone A. Lencioni M. Brunetti I. Pfanner E. Allegrini G. Antonuzzo A. (1997). Maximum tolerable doses of intravenous zidovudine in combination with 5-fluorouracil and leucovorin in metastatic colorectal cancer patients. Clinical evidence of significant antitumor activity and enhancement of zidovudine-induced DNA single strand breaks in peripheral nuclear blood cells. Ann. Oncol. 8, 539–545. 10.1023/a:1008249803523 9261522

[B84] Favoulet P. Cercueil J. P. Faure P. Osmak L. Isambert N. Beltramo J. L. (2001). Increased cytotoxicity and stability of Lipiodol-pirarubicin emulsion compared to classical doxorubicin-Lipiodol: potential advantage for chemoembolization of unresectable hepatocellular carcinoma. Anticancer Drugs 12, 801–806. 10.1097/00001813-200111000-00003 11707647

[B85] Ferioli M. Zauli G. Martelli A. M. Vitale M. McCubrey J. A. Ultimo S. (2018). Impact of physical exercise in cancer survivors during and after antineoplastic treatments. Oncotarget 9, 14005–14034. 10.18632/oncotarget.24456 29568412 PMC5862633

[B86] Ferlay J. Colombet M. Soerjomataram I. Parkin D. M. Piñeros M. Znaor A. (2021). Estimating the global cancer incidence and mortality in 2018: GLOBOCAN sources and methods. Int. J. Cancer. 144, 1941–1953. 10.1002/ijc.31937 30350310

[B87] Fernandez-Banet J. Esposito A. Coffin S. Horvath I. B. Estrella H. Schefzick S. (2016). OASIS: web-based platform for exploring cancer multi-omics data. Nat. Methods 13, 9–10. 10.1038/nmeth.3692 26716558

[B88] Fernández-Torras A. Duran-Frigola M. Aloy P. (2019). Encircling the regions of the pharmacogenomic landscape that determine drug response. Genome Med. 11, 17. 10.1186/s13073-019-0626-x 30914058 PMC6436215

[B89] Florio R. Veschi S. di Giacomo V. Pagotto S. Carradori S. Verginelli F. (2019). The benzimidazole-based anthelmintic parbendazole: a repurposed drug candidate that synergizes with gemcitabine in pancreatic cancer. Cancers 11, 2042. 10.3390/cancers11122042 31861153 PMC6966614

[B90] Foglietta F. Pinnelli V. Giuntini F. Barbero N. Panzanelli P. Durando G. (2021). Sonodynamic treatment induces selective killing of cancer cells in an *in vitro* co-culture model. Cancers 13, 3852. 10.3390/cancers13153852 34359753 PMC8345649

[B91] Freires I. A. Sardi J. de C. O. de Castro R. D. Rosalen P. L. (2017). Alternative animal and non-animal models for drug discovery and development: bonus or burden? Pharm. Res. 34, 681–686. 10.1007/s11095-016-2069-z 27858217

[B92] Frouws M. A. Rademaker E. Bastiaannet E. van Herk-Sukel M. P. P. Lemmens V. E. Van de Velde C. J. H. (2017). The difference in association between aspirin use and other thrombocyte aggregation inhibitors and survival in patients with colorectal cancer. Eur. J. Cancer 77, 24–30. 10.1016/j.ejca.2017.02.025 28350995

[B93] Fuchs C. S. Tomasek J. Yong C. J. Dumitru F. Passalacqua R. Goswami C. (2014). Ramucirumab monotherapy for previously treated advanced gastric or gastro-oesophageal junction adenocarcinoma (REGARD): an international, randomised, multicentre, placebo-controlled, phase 3 trial. Lancet 383, 31–39. 10.1016/S0140-6736(13)61719-5 24094768

[B94] Gao F. Wu J. Niu S. Sun T. Li F. Bai Y. (2019). Biodegradable, pH-sensitive hollow mesoporous organosilica nanoparticle (HMON) with controlled release of pirfenidone and ultrasound-target-microbubble-destruction (UTMD) for pancreatic cancer treatment. Theranostics 9, 6002–6018. 10.7150/thno.36135 31534533 PMC6735371

[B95] Gao X. Liu X. Shan W. Liu Q. Wang C. Zheng J. (2018). Anti-malarial atovaquone exhibits anti-tumor effects by inducing DNA damage in hepatocellular carcinoma. Am. J. Cancer Res. 8, 1697–1711. 30323964 PMC6176191

[B96] Gdovinova Z. Kremer C. Lorenzano S. Dawson J. Lal A. Caso V. (2022). Aspirin for primary stroke prevention; evidence for a differential effect in men and women. Front. Neurol. 13, 856239. 10.3389/fneur.2022.856239 35800088 PMC9254866

[B97] Ghasemi K. Ghasemi K. (2022). A Brief look at antitumor effects of doxycycline in the treatment of colorectal cancer and combination therapies. Eur. J. Pharmacol. 916, 174593. 10.1016/j.ejphar.2021.174593 34973952

[B98] Gong R.-H. Yang D.-J. Kwan H.-Y. Lyu A.-P. Chen G.-Q. Bian Z.-X. (2022). Cell death mechanisms induced by synergistic effects of halofuginone and artemisinin in colorectal cancer cells. Int. J. Med. Sci. 19, 175–185. 10.7150/ijms.66737 34975311 PMC8692125

[B99] Gordon S. W. McGuire W. P. 3rd Shafer D. A. Sterling R. K. Lee H. M. Matherly S. C. (2019). Phase I study of sorafenib and vorinostat in advanced hepatocellular carcinoma. Am. J. Clin. Oncol. 42, 649–654. 10.1097/COC.0000000000000567 31305287

[B100] Gou H. Liu S. Liu L. Luo M. Qin S. He K. (2022). Obeticholic acid and 5β-cholanic acid 3 exhibit anti-tumor effects on liver cancer through CXCL16/CXCR6 pathway. Front. Immunol. 13, 1095915. 10.3389/fimmu.2022.1095915 36605219 PMC9807878

[B101] Gray E. Marti J. Wyatt J. C. Brewster D. H. Hall P. S. SATURNE advisory group (2019). Chemotherapy effectiveness in trial-underrepresented groups with early breast cancer: a retrospective cohort study. PLoS Med. 16, e1003006. 10.1371/journal.pmed.1003006 31891574 PMC6938317

[B102] Greene C. S. Krishnan A. Wong A. K. Ricciotti E. Zelaya R. A. Himmelstein D. S. (2015). Understanding multicellular function and disease with human tissue-specific networks. Nat. Genet. 47, 569–576. 10.1038/ng.3259 25915600 PMC4828725

[B103] Greene C. S. Voight B. F. (2016). Pathway and network-based strategies to translate genetic discoveries into effective therapies. Hum. Mol. Genet. 25, R94–R98. 10.1093/hmg/ddw160 27340225 PMC5036870

[B104] Greten T. F. Manns M. P. Korangy F. (2008). Immunotherapy of HCC. Rev. Recent Clin. Trials 3, 31–39. 10.2174/157488708783330549 18474013

[B105] Grösch S. Tegeder I. Niederberger E. Bräutigam L. Geisslinger G. (2001). COX-2 independent induction of cell cycle arrest and apoptosis in colon cancer cells by the selective COX-2 inhibitor celecoxib. FASEB J. 15, 2742–2744. 10.1096/fj.01-0299fje 11606477

[B106] Grover M. P. Ballouz S. Mohanasundaram K. A. George R. A. Goscinski A. Crowley T. M. (2015). Novel therapeutics for coronary artery disease from genome-wide association study data. BMC Med. Genomics 8 (2), S1. 10.1186/1755-8794-8-S2-S1 PMC4460746 26044129

[B107] Guirguis-BlakeJ. M.EvansC. V.PerdueL. A.BeanS. I.SengerC. A. (2022). Aspirin use to prevent cardiovascular disease and colorectal cancer: updated evidence report and systematic review for the US preventive services task force. JAMA 327, 1585–1597. 10.1001/jama.2022.3337 35471507

[B108] Gulhati P. Cai Q. Li J. Liu J. Rychahou P. G. Qiu S. (2009). Targeted inhibition of mammalian target of rapamycin signaling inhibits tumorigenesis of colorectal cancer. Clin. Cancer Res. 15, 7207–7216. 10.1158/1078-0432.CCR-09-1249 19934294 PMC2898570

[B109] Gunderson A. J. Kaneda M. M. Tsujikawa T. Nguyen A. V. Affara N. I. Ruffell B. (2016). Bruton tyrosine kinase-dependent immune cell cross-talk drives pancreas cancer. Cancer Discov. 6, 270–285. 10.1158/2159-8290.CD-15-0827 26715645 PMC4783268

[B110] Guo L. Chen X.-J. Hu Y.-H. Yu Z.-J. Wang D. Liu J.-Z. (2013). Curcumin inhibits proliferation and induces apoptosis of human colorectal cancer cells by activating the mitochondria apoptotic pathway. Phytother. Res. 27, 422–430. 10.1002/ptr.4731 22628241

[B111] Gupta S. C. Sung B. Prasad S. Webb L. J. Aggarwal B. B. (2013). Cancer drug discovery by repurposing: teaching new tricks to old dogs. Trends Pharmacol. Sci. 34, 508–517. 10.1016/j.tips.2013.06.005 23928289

[B112] Gut M. O. Parkkila S. Vernerová Z. Rohde E. Závada J. Höcker M. (2002). Gastric hyperplasia in mice with targeted disruption of the carbonic anhydrase gene Car9. Gastroenterology 123, 1889–1903. 10.1053/gast.2002.37052 12454846

[B113] Hanada Y. Pereira S. P. Pogue B. Maytin E. V. Hasan T. Linn B. (2021). EUS-guided verteporfin photodynamic therapy for pancreatic cancer. Gastrointest. Endosc. 94, 179–186. 10.1016/j.gie.2021.02.027 33647286 PMC10434704

[B114] Happold C. Gorlia T. Chinot O. Gilbert M. R. Nabors L. B. Wick W. (2016). Does valproic acid or levetiracetam improve survival in glioblastoma? A pooled analysis of prospective clinical trials in newly diagnosed glioblastoma. J. Clin. Oncol. 34, 731–739. 10.1200/JCO.2015.63.6563 26786929 PMC5070573

[B115] He S. Xu J. Liu X. Zhen Y. (2021). Advances and challenges in the treatment of esophageal cancer. Acta Pharm. Sin. B 11, 3379–3392. 10.1016/j.apsb.2021.03.008 34900524 PMC8642427

[B116] Hecht M. Harrer T. Büttner M. Schwegler M. Erber S. Fietkau R. (2013). Cytotoxic effect of efavirenz is selective against cancer cells and associated with the cannabinoid system. AIDS 27, 2031–2040. 10.1097/QAD.0b013e3283625444 23612009

[B117] Hecht M. Harrer T. Körber V. Sarpong E. O. Moser F. Fiebig N. (2018). Cytotoxic effect of Efavirenz in BxPC-3 pancreatic cancer cells is based on oxidative stress and is synergistic with ionizing radiation. Oncol. Lett. 15, 1728–1736. 10.3892/ol.2017.7523 29434868 PMC5776903

[B118] Heer E. Ruan Y. Mah B. Nguyen T. Lyons H. Poirier A. (2022). The efficacy of chemopreventive agents on the incidence of colorectal adenomas: a systematic review and network meta-analysis. Prev. Med. 162, 107169. 10.1016/j.ypmed.2022.107169 35878711

[B119] Hegazy S. K. El-Azab G. A. Zakaria F. Mostafa M. F. El-Ghoneimy R. A. (2022). Mebendazole; from an anti-parasitic drug to a promising candidate for drug repurposing in colorectal cancer. Life Sci. 299, 120536. 10.1016/j.lfs.2022.120536 35385794

[B120] Hieronymus H. Lamb J. Ross K. N. Peng X. P. Clement C. Rodina A. (2006). Gene expression signature-based chemical genomic prediction identifies a novel class of HSP90 pathway modulators. Cancer Cell 10, 321–330. 10.1016/j.ccr.2006.09.005 17010675

[B121] Higurashi T. Nakajima A. (2018). Metformin and colorectal cancer. Front. Endocrinol. 9, 622. 10.3389/fendo.2018.00622 PMC6205961 30405532

[B122] Hirt C. K. Booij T. H. Grob L. Simmler P. Toussaint N. C. Keller D. (2022). Drug screening and genome editing in human pancreatic cancer organoids identifies drug-gene interactions and candidates for off-label treatment. Cell Genom 2, 100095. 10.1016/j.xgen.2022.100095 35187519 PMC7612395

[B123] Honarparvar B. Govender T. Maguire G. E. M. Soliman M. E. S. Kruger H. G. (2014). Integrated approach to structure-based enzymatic drug design: molecular modeling, spectroscopy, and experimental bioactivity. Chem. Rev. 114, 493–537. 10.1021/cr300314q 24024775

[B124] Hossain M. T. Reza M. S. Peng Y. Feng S. Wei Y. (2023). Identification of key genes as potential drug targets for gastric cancer. Tsinghua Sci. Technol. 28, 649–664. 10.26599/tst.2022.9010035

[B125] Hu S. Liu L. Chang E. B. Wang J.-Y. Raufman J.-P. (2015). Butyrate inhibits pro-proliferative miR-92a by diminishing c-Myc-induced miR-17-92a cluster transcription in human colon cancer cells. Mol. Cancer 14, 180. 10.1186/s12943-015-0450-x 26463716 PMC4604099

[B126] Huang C. Lan W. Fraunhoffer N. Meilerman A. Iovanna J. Santofimia-Castaño P. (2019). Dissecting the anticancer mechanism of trifluoperazine on pancreatic ductal adenocarcinoma. Cancers 11, 1869. 10.3390/cancers11121869 31769431 PMC6966621

[B127] Huang J. Lok V. Ngai C. H. Zhang L. Yuan J. Lao X. Q. (2021). Worldwide burden of, risk factors for, and trends in pancreatic cancer. Gastroenterology 160, 744–754. 10.1053/j.gastro.2020.10.007 33058868

[B128] Huggett M. T. Jermyn M. Gillams A. Illing R. Mosse S. Novelli M. (2014). Phase I/II study of verteporfin photodynamic therapy in locally advanced pancreatic cancer. Br. J. Cancer 110, 1698–1704. 10.1038/bjc.2014.95 24569464 PMC3974098

[B129] Hui B. Shi Y.-H. Ding Z.-B. Zhou J. Gu C.-Y. Peng Y.-F. (2012). Proteasome inhibitor interacts synergistically with autophagy inhibitor to suppress proliferation and induce apoptosis in hepatocellular carcinoma. Cancer 118, 5560–5571. 10.1002/cncr.27586 22517429

[B130] Hulkower K. I. Herber R. L. (2011). Cell migration and invasion assays as tools for drug discovery. Pharmaceutics 3, 107–124. 10.3390/pharmaceutics3010107 24310428 PMC3857040

[B131] Hurle M. R. Yang L. Xie Q. Rajpal D. K. Sanseau P. Agarwal P. (2013). Computational drug repositioning: from data to therapeutics. Clin. Pharmacol. Ther. 93, 335–341. 10.1038/clpt.2013.1 23443757

[B132] Iida T. Mizutani Y. Esaki N. Ponik S. M. Burkel B. M. Weng L. (2022). Pharmacologic conversion of cancer-associated fibroblasts from a protumor phenotype to an antitumor phenotype improves the sensitivity of pancreatic cancer to chemotherapeutics. Oncogene 41, 2764–2777. 10.1038/s41388-022-02288-9 35414659

[B133] Iljin K. Ketola K. Vainio P. Halonen P. Kohonen P. Fey V. (2009). High-throughput cell-based screening of 4910 known drugs and drug-like small molecules identifies disulfiram as an inhibitor of prostate cancer cell growth. Clin. Cancer Res. 15, 6070–6078. 10.1158/1078-0432.CCR-09-1035 19789329

[B134] Iorio F. Bosotti R. Scacheri E. Belcastro V. Mithbaokar P. Ferriero R. (2010). Discovery of drug mode of action and drug repositioning from transcriptional responses. Proc. Natl. Acad. Sci. U. S. A. 107, 14621–14626. 10.1073/pnas.1000138107 20679242 PMC2930479

[B135] Iorio F. Saez-Rodriguez J. di Bernardo D. (2013). Network based elucidation of drug response: from modulators to targets. BMC Syst. Biol. 7, 139. 10.1186/1752-0509-7-139 24330611 PMC3878740

[B136] Jandaghi P. Najafabadi H. S. Bauer A. S. Papadakis A. I. Fassan M. Hall A. (2016). Expression of DRD2 is increased in human pancreatic ductal adenocarcinoma and inhibitors slow tumor growth in mice. Gastroenterology 151, 1218–1231. 10.1053/j.gastro.2016.08.040 27578530

[B137] Jeon Y. Sym S. J. Yoo B. K. Baek J.-H. (2022). Long-term survival, tolerability, and safety of first-line bevacizumab and FOLFIRI in combination with ginsenoside-modified nanostructured lipid carrier containing curcumin in patients with unresectable metastatic colorectal cancer. Integr. Cancer Ther. 21, 15347354221105498. 10.1177/15347354221105498 35695002 PMC9202259

[B138] Jeong J. B. Choi J. Baek S. J. Lee S.-H. (2013). Reactive oxygen species mediate tolfenamic acid-induced apoptosis in human colorectal cancer cells. Arch. Biochem. Biophys. 537, 168–175. 10.1016/j.abb.2013.07.016 23896517

[B139] Ji L. Qian W. Gui L. Ji Z. Yin P. Lin G. N. (2020). Blockade of β-catenin-induced CCL28 suppresses gastric cancer progression via inhibition of treg cell infiltration. Cancer Res. 80, 2004–2016. 10.1158/0008-5472.CAN-19-3074 32156780

[B140] Ji T. Lang J. Wang J. Cai R. Zhang Y. Qi F. (2017). Designing liposomes to suppress extracellular matrix expression to enhance drug penetration and pancreatic tumor therapy. ACS Nano 11, 8668–8678. 10.1021/acsnano.7b01026 28806504

[B141] Jiang F. Xing H.-S. Chen W.-Y. Du J. Ruan Y.-L. Lin A.-Y. (2019). Itraconazole inhibits proliferation of pancreatic cancer cells through activation of Bak-1. J. Cell. Biochem. 120, 4333–4341. 10.1002/jcb.27719 30260036

[B142] Jian-Hui C. Er-Tao Z. Si-Le C. Hui W. Kai-Ming W. Xin-Hua Z. (2016). CD44, sonic hedgehog, and Gli1 expression are prognostic biomarkers in gastric cancer patients after radical resection. Gastroenterol. Res. Pract. 2016, 1013045. 10.1155/2016/1013045 26839535 PMC4709647

[B143] Jin H. Wang S. Zaal E. A. Wang C. Wu H. Bosma A. (2020). A powerful drug combination strategy targeting glutamine addiction for the treatment of human liver cancer. Elife 9, e56749. 10.7554/eLife.56749 33016874 PMC7535927

[B144] Jin R. U. Mills J. C. (2020). Tumor organoids to study gastroesophageal cancer: a primer. J. Mol. Cell Biol. 12, 593–606. 10.1093/jmcb/mjaa035 32652008 PMC7683018

[B145] Jin X. Pan Y. Wang L. Zhang L. Ravichandran R. Potts P. R. (2017). MAGE-TRIM28 complex promotes the Warburg effect and hepatocellular carcinoma progression by targeting FBP1 for degradation. Oncogenesis 6, e312. 10.1038/oncsis.2017.21 28394358 PMC5520498

[B146] Joshi S. S. Badgwell B. D. (2021). Current treatment and recent progress in gastric cancer. CA Cancer J. Clin. 71, 264–279. 10.3322/caac.21657 33592120 PMC9927927

[B147] Kaji K. Nishimura N. Seki K. Sato S. Saikawa S. Nakanishi K. (2018). Sodium glucose cotransporter 2 inhibitor canagliflozin attenuates liver cancer cell growth and angiogenic activity by inhibiting glucose uptake. Int. J. Cancer 142, 1712–1722. 10.1002/ijc.31193 29205334

[B148] Kan J.-Y. Hsu Y.-L. Chen Y.-H. Chen T.-C. Wang J.-Y. Kuo P.-L. (2013). Gemifloxacin, a fluoroquinolone antimicrobial drug, inhibits migration and invasion of human colon cancer cells. Biomed. Res. Int. 2013, 159786. 10.1155/2013/159786 24386633 PMC3872387

[B149] Kang H. J. Seol H. S. Lee S. E. Suh Y.-A. Kim J. Jang S. J. (2019). Guanabenz acetate induces endoplasmic reticulum stress-related cell death in hepatocellular carcinoma cells. J. Pathol. Transl. Med. 53, 94–103. 10.4132/jptm.2019.01.14 30646673 PMC6435987

[B150] Kannen V. Hintzsche H. Zanette D. L. Silva W. A. Jr Garcia S. B. Waaga-Gasser A. M. (2012). Antiproliferative effects of fluoxetine on colon cancer cells and in a colonic carcinogen mouse model. PLoS One 7, e50043. 10.1371/journal.pone.0050043 23209640 PMC3507893

[B151] Karaman M. W. Herrgard S. Treiber D. K. Gallant P. Atteridge C. E. Campbell B. T. (2008). A quantitative analysis of kinase inhibitor selectivity. Nat. Biotechnol. 26, 127–132. 10.1038/nbt1358 18183025

[B152] Karnevi E. Said K. Andersson R. Rosendahl A. H. (2013). Metformin-mediated growth inhibition involves suppression of the IGF-I receptor signalling pathway in human pancreatic cancer cells. BMC Cancer 13, 235. 10.1186/1471-2407-13-235 23663483 PMC3661399

[B153] Kaur J. Sanyal S. N. (2011). Diclofenac, a selective COX-2 inhibitor, inhibits DMH-induced colon tumorigenesis through suppression of MCP-1, MIP-1α and VEGF. Mol. Carcinog. 50, 707–718. 10.1002/mc.20736 21268133

[B154] Keiser M. J. Setola V. Irwin J. J. Laggner C. Abbas A. I. Hufeisen S. J. (2009). Predicting new molecular targets for known drugs. Nature 462, 175–181. 10.1038/nature08506 19881490 PMC2784146

[B155] Kersten K. de Visser K. E. van Miltenburg M. H. Jonkers J. (2017). Genetically engineered mouse models in oncology research and cancer medicine. EMBO Mol. Med. 9, 137–153. 10.15252/emmm.201606857 28028012 PMC5286388

[B156] Khozin S. Blumenthal G. M. Pazdur R. (2017). Real-world data for clinical evidence generation in oncology. J. Natl. Cancer Inst. 109. 10.1093/jnci/djx187 29059439

[B157] Kim H. J. Oh S. C. (2018). Novel systemic therapies for advanced gastric cancer. J. Gastric Cancer 18, 1–19. 10.5230/jgc.2018.18.e3 29629216 PMC5881006

[B158] Kim J. Yoo M. Shin J. Kim H. Kang J. Tan A. C. (2018). Systems pharmacology-based approach of connecting disease genes in genome-wide association studies with traditional Chinese medicine. Int. J. Genomics Proteomics 2018, 7697356. 10.1155/2018/7697356 PMC5885494 29765977

[B159] Kim M. Mun H. Sung C. O. Cho E. J. Jeon H.-J. Chun S.-M. (2019). Patient-derived lung cancer organoids as *in vitro* cancer models for therapeutic screening. Nat. Commun. 10, 3991. 10.1038/s41467-019-11867-6 31488816 PMC6728380

[B160] Kim S. H. Lee H. Y. Yi H. Ahn Y. M. Kim Y. S. (2012). Haloperidol induces demethylation and expression of the dual specificity phosphatase 6 gene in MIA PaCa-2 human pancreatic cancer cells. Life Sci. 91, 1317–1322. 10.1016/j.lfs.2012.10.002 23063941

[B161] Knapp S. (2018). New opportunities for kinase drug repurposing and target discovery. Br. J. Cancer 118, 936–937. 10.1038/s41416-018-0045-6 29545596 PMC5931101

[B162] Kobayashi H. Enomoto A. Woods S. L. Burt A. D. Takahashi M. Worthley D. L. (2019). Cancer-associated fibroblasts in gastrointestinal cancer. Nat. Rev. Gastroenterol. Hepatol. 16, 282–295. 10.1038/s41575-019-0115-0 30778141

[B163] Kobayashi H. Gieniec K. A. Wright J. A. Wang T. Asai N. Mizutani Y. (2021). The balance of stromal BMP signaling mediated by GREM1 and ISLR drives colorectal carcinogenesis. Gastroenterology 160, 1224–1239. 10.1053/j.gastro.2020.11.011 33197448 PMC7617122

[B164] Kobayashi Y. Kashima H. Rahmanto Y. S. Banno K. Yu Y. Matoba Y. (2017). Drug repositioning of mevalonate pathway inhibitors as antitumor agents for ovarian cancer. Oncotarget 8, 72147–72156. 10.18632/oncotarget.20046 29069775 PMC5641118

[B165] Kong K. Guo M. Liu Y. Zheng J. (2020). Progress in animal models of pancreatic ductal adenocarcinoma. J. Cancer 11, 1555–1567. 10.7150/jca.37529 32047562 PMC6995380

[B166] Kordes S. Pollak M. N. Zwinderman A. H. Mathôt R. A. Weterman M. J. Beeker A. (2015). Metformin in patients with advanced pancreatic cancer: a double-blind, randomised, placebo-controlled phase 2 trial. Lancet Oncol. 16, 839–847. 10.1016/S1470-2045(15)00027-3 26067687

[B167] Kulkarni V. S. Alagarsamy V. Solomon V. R. Jose P. A. Murugesan S. (2023). Drug repurposing: an effective tool in modern drug discovery. Russ. J. Bioorg. Chem. 49, 157–166. 10.1134/S1068162023020139 36852389 PMC9945820

[B168] Kumar V. L. Verma S. Das P. (2019). Artesunate suppresses inflammation and oxidative stress in a rat model of colorectal cancer. Drug Dev. Res. 80, 1089–1097. 10.1002/ddr.21590 31471932

[B169] Lai C.-T. Chi C.-W. Wu S.-H. Shieh H.-R. Yen J.-C. Chen Y.-J. (2022). Midostaurin modulates tumor microenvironment and enhances efficacy of anti-PD-1 against colon cancer. Cancers 14, 4847. 10.3390/cancers14194847 36230769 PMC9563721

[B170] Lally J. S. V. Ghoshal S. DePeralta D. K. Moaven O. Wei L. Masia R. (2019). Inhibition of acetyl-CoA carboxylase by phosphorylation or the inhibitor ND-654 suppresses lipogenesis and hepatocellular carcinoma. Cell Metab. 29, 174–182. 10.1016/j.cmet.2018.08.020 30244972 PMC6643297

[B171] Lan S.-H. Wu S.-Y. Zuchini R. Lin X.-Z. Su I.-J. Tsai T.-F. (2014). Autophagy suppresses tumorigenesis of hepatitis B virus-associated hepatocellular carcinoma through degradation of microRNA-224. Hepatology 59, 505–517. 10.1002/hep.26659 23913306 PMC4298796

[B172] Lee J. Lee I. Han B. Park J. O. Jang J. Park C. (2011). Effect of simvastatin on cetuximab resistance in human colorectal cancer with KRAS mutations. J. Natl. Cancer Inst. 103, 674–688. 10.1093/jnci/djr070 21398618

[B173] Leng X. Yang J. Liu T. Zhao C. Cao Z. Li C. (2022). A bioinformatics framework to identify the biomarkers and potential drugs for the treatment of colorectal cancer. Front. Genet. 13, 1017539. 10.3389/fgene.2022.1017539 36238159 PMC9551025

[B174] Li J. Chen K. Wang F. Dai W. Li S. Feng J. (2017a). Methyl jasmonate leads to necrosis and apoptosis in hepatocellular carcinoma cells via inhibition of glycolysis and represses tumor growth in mice. Oncotarget 8, 45965–45980. 10.18632/oncotarget.17469 28498814 PMC5542241

[B175] Li J. Van Vranken J. G. Pontano Vaites L. Schweppe D. K. Huttlin E. L. Etienne C. (2020a). TMTpro reagents: a set of isobaric labeling mass tags enables simultaneous proteome-wide measurements across 16 samples. Nat. Methods 17, 399–404. 10.1038/s41592-020-0781-4 32203386 PMC7302421

[B176] Li J. Xu R. Qin S. Liu T. Pan H. Xu J. (2018a). Aflibercept plus FOLFIRI in Asian patients with pretreated metastatic colorectal cancer: a randomized Phase III study. Future Oncol. 14, 2031–2044. 10.2217/fon-2017-0669 30117334

[B177] Li M. Fisher W. E. Kim H. J. Wang X. Brunicardi C. F. Chen C. (2005). Somatostatin, somatostatin receptors, and pancreatic cancer. World J. Surg. 29, 293–296. 10.1007/s00268-004-7814-5 15706439

[B178] Li M. Xu H. (2019). Fear stress enhanced xenograft pancreatic tumor growth through activating epithelial-mesenchymal transition. Pancreatology 19, 377–382. 10.1016/j.pan.2019.01.002 30733163

[B179] Li N. Wu P. Shen Y. Yang C. Zhang L. Chen Y. (2021a). Predictions of mortality related to four major cancers in China, 2020 to 2030. Cancer Commun. 41, 404–413. 10.1002/cac2.12143 PMC8118592 33660417

[B180] Li S. Li J. Dai W. Zhang Q. Feng J. Wu L. (2017b). Genistein suppresses aerobic glycolysis and induces hepatocellular carcinoma cell death. Br. J. Cancer 117, 1518–1528. 10.1038/bjc.2017.323 28926527 PMC5680469

[B181] Li X. Francies H. E. Secrier M. Perner J. Miremadi A. Galeano-Dalmau N. (2018). Organoid cultures recapitulate esophageal adenocarcinoma heterogeneity providing a model for clonality studies and precision therapeutics. Nat. Commun. 9, 2983. 10.1038/s41467-018-05190-9 30061675 PMC6065407

[B182] Li Y. Li Y. Li D. Li K. Quan Z. Wang Z. (2020b). Repositioning of hypoglycemic drug linagliptin for cancer treatment. Front. Pharmacol. 11, 187. 10.3389/fphar.2020.00187 32194417 PMC7062795

[B183] Li Y. Y. Jones S. J. (2012). Drug repositioning for personalized medicine. Genome Med. 4, 27. 10.1186/gm326 22494857 PMC3446277

[B184] Li Z. Zou L. Xiao Z.-X. Yang J. (2022). Transcriptome-based drug repositioning identifies TPCA-1 as a potential selective inhibitor of esophagus squamous carcinoma cell viability. Int. J. Mol. Med. 49, 75. 10.3892/ijmm.2022.5131 35417037 PMC9015666

[B185] Liao P. Song K. Zhu Z. Liu Z. Zhang W. Li W. (2020). Propranolol suppresses the growth of colorectal cancer through simultaneously activating autologous CD8+ T cells and inhibiting tumor AKT/MAPK pathway. Clin. Pharmacol. Ther. 108, 606–615. 10.1002/cpt.1894 32418204

[B186] Lim H. He D. Qiu Y. Krawczuk P. Sun X. Xie L. (2019). Rational discovery of dual-indication multi-target PDE/Kinase inhibitor for precision anti-cancer therapy using structural systems pharmacology. PLoS Comput. Biol. 15, e1006619. 10.1371/journal.pcbi.1006619 31206508 PMC6576746

[B187] Lin C.-C. Suen K. M. Stainthorp A. Wieteska L. Biggs G. S. Leitão A. (2019). Targeting the Shc-EGFR interaction with indomethacin inhibits MAP kinase pathway signalling. Cancer Lett. 457, 86–97. 10.1016/j.canlet.2019.05.008 31100409 PMC6584941

[B188] Liñares-Blanco J. Munteanu C. R. Pazos A. Fernandez-Lozano C. (2020). Molecular docking and machine learning analysis of Abemaciclib in colon cancer. BMC Mol. Cell Biol. 21, 52. 10.1186/s12860-020-00295-w 32640984 PMC7346626

[B189] Liu H. Tao H. Wang H. Yang Y. Yang R. Dai X. (2020a). Doxycycline inhibits cancer stem cell-like properties *via* PAR1/FAK/PI3K/AKT pathway in pancreatic cancer. Front. Oncol. 10, 619317. 10.3389/fonc.2020.619317 33643917 PMC7905084

[B190] Liu P.-F. Tsai K.-L. Hsu C.-J. Tsai W.-L. Cheng J.-S. Chang H.-W. (2018). Drug repurposing screening identifies tioconazole as an ATG4 inhibitor that suppresses autophagy and sensitizes cancer cells to chemotherapy. Theranostics 8, 830–845. 10.7150/thno.22012 29344310 PMC5771097

[B191] Liu S.-H. Yu J. Creeden J. F. Sutton J. M. Markowiak S. Sanchez R. (2020b). Repurposing metformin, simvastatin and digoxin as a combination for targeted therapy for pancreatic ductal adenocarcinoma. Cancer Lett. 491, 97–107. 10.1016/j.canlet.2020.08.002 32829010 PMC8766172

[B192] Liu T. Li S. Wu L. Yu Q. Li J. Feng J. (2020c). Experimental study of hepatocellular carcinoma treatment by shikonin through regulating PKM2. J. Hepatocell. Carcinoma 7, 19–31. 10.2147/JHC.S237614 32110554 PMC7035901

[B193] Liu X. He M. Li L. Wang X. Han S. Zhao J. (2021). EMT and cancer cell stemness associated with chemotherapeutic resistance in esophageal cancer. Front. Oncol. 11, 672222. 10.3389/fonc.2021.672222 34150636 PMC8209423

[B194] Long Q. Ao L. Li K. Li Y. (2020). The efficacy and safety of sulindac for colorectal polyps: a protocol for systematic review and meta-analysis. Medicine 99, e22402. 10.1097/MD.0000000000022402 33031275 PMC7544282

[B195] Low Y. S. Daugherty A. C. Schroeder E. A. Chen W. Seto T. Weber S. (2017). Synergistic drug combinations from electronic health records and gene expression. J. Am. Med. Inf. Assoc. 24, 565–576. 10.1093/jamia/ocw161 PMC6080645 27940607

[B196] Lübbert M. Grishina O. Schmoor C. Schlenk R. F. Jost E. Crysandt M. (2020). Valproate and retinoic acid in combination with decitabine in elderly nonfit patients with acute myeloid leukemia: results of a multicenter, randomized, 2 × 2, phase II trial. J. Clin. Oncol. 38, 257–270. 10.1200/JCO.19.01053 31794324

[B197] Luhn P. Kuk D. Carrigan G. Nussbaum N. Sorg R. Rohrer R. (2019). Validation of diagnosis codes to identify side of colon in an electronic health record registry. BMC Med. Res. Methodol. 19, 177. 10.1186/s12874-019-0824-7 31426736 PMC6700780

[B198] Luo Y. Zhao X. Zhou J. Yang J. Zhang Y. Kuang W. (2017). A network integration approach for drug-target interaction prediction and computational drug repositioning from heterogeneous information. Nat. Commun. 8, 573. 10.1038/s41467-017-00680-8 28924171 PMC5603535

[B199] Ma M. K. F. Lau E. Y. T. Leung D. H. W. Lo J. Ho N. P. Y. Cheng L. K. W. (2017). Stearoyl-CoA desaturase regulates sorafenib resistance via modulation of ER stress-induced differentiation. J. Hepatol. 67, 979–990. 10.1016/j.jhep.2017.06.015 28647567

[B200] Mao Y. Wang W. Yang J. Zhou X. Lu Y. Gao J. (2023). Drug repurposing screening and mechanism analysis based on human colorectal cancer organoids. Protein Cell, pwad038. 10.1093/procel/pwad038 37345888 PMC10984622

[B201] Martinez Molina D. Jafari R. Ignatushchenko M. Seki T. Larsson E. A. Dan C. (2013). Monitoring drug target engagement in cells and tissues using the cellular thermal shift assay. Science 341, 84–87. 10.1126/science.1233606 23828940

[B202] Martinez-Pacheco S. O’Driscoll L. (2021). Pre-clinical *in vitro* models used in cancer research: results of a worldwide survey. Cancers 13, 6033. 10.3390/cancers13236033 34885142 PMC8656628

[B203] Massó-Vallés D. Jauset T. Serrano E. Sodir N. M. Pedersen K. Affara N. I. (2015). Ibrutinib exerts potent antifibrotic and antitumor activities in mouse models of pancreatic adenocarcinoma. Cancer Res. 75, 1675–1681. 10.1158/0008-5472.CAN-14-2852 25878147 PMC6773609

[B204] Mateus A. Kurzawa N. Becher I. Sridharan S. Helm D. Stein F. (2020). Thermal proteome profiling for interrogating protein interactions. Mol. Syst. Biol. 16, e9232. 10.15252/msb.20199232 32133759 PMC7057112

[B205] Mazumder S. De R. Debsharma S. Bindu S. Maity P. Sarkar S. (2019). Indomethacin impairs mitochondrial dynamics by activating the PKCζ-p38-DRP1 pathway and inducing apoptosis in gastric cancer and normal mucosal cells. J. Biol. Chem. 294, 8238–8258. 10.1074/jbc.RA118.004415 30940726 PMC6527165

[B206] McGuigan A. Kelly P. Turkington R. C. Jones C. Coleman H. G. McCain R. S. (2018). Pancreatic cancer: a review of clinical diagnosis, epidemiology, treatment and outcomes. World J. Gastroenterol. 24, 4846–4861. 10.3748/wjg.v24.i43.4846 30487695 PMC6250924

[B207] Meyn R. E. Krishnan S. Skinner H. D. (2016). Everything old is new again: using nelfinavir to radiosensitize rectal cancer. Clin. Cancer Res. 22, 1834–1836. 10.1158/1078-0432.CCR-16-0024 26920893 PMC5061034

[B208] Midgley R. S. McConkey C. C. Johnstone E. C. Dunn J. A. Smith J. L. Grumett S. A. (2010). Phase III randomized trial assessing rofecoxib in the adjuvant setting of colorectal cancer: final results of the VICTOR trial. J. Clin. Oncol. 28, 4575–4580. 10.1200/JCO.2010.29.6244 20837956

[B209] Miller K. D. Siegel R. L. Lin C. C. Mariotto A. B. Kramer J. L. Rowland J. H. (2016). Cancer treatment and survivorship statistics, 2016. CA Cancer J. Clin. 66, 271–289. 10.3322/caac.21349 27253694

[B210] Miriovsky B. J. Shulman L. N. Abernethy A. P. (2012). Importance of health information technology, electronic health records, and continuously aggregating data to comparative effectiveness research and learning health care. J. Clin. Oncol. 30, 4243–4248. 10.1200/JCO.2012.42.8011 23071233

[B211] Mitrović A. Kos J. (2019). Nitroxoline: repurposing its antimicrobial to antitumor application. Acta Biochim. Pol. 66, 521–531. 10.18388/abp.2019_2904 31834689

[B212] Mizutani Y. Iida T. Ohno E. Ishikawa T. Kinoshita F. Kuwatsuka Y. (2022). Safety and efficacy of MIKE-1 in patients with advanced pancreatic cancer: a study protocol for an open-label phase I/II investigator-initiated clinical trial based on a drug repositioning approach that reprograms the tumour stroma. BMC Cancer 22, 205. 10.1186/s12885-022-09272-2 35209871 PMC8867831

[B213] Mizutani Y. Kobayashi H. Iida T. Asai N. Masamune A. Hara A. (2019). Meflin-positive cancer-associated fibroblasts inhibit pancreatic carcinogenesis. Cancer Res. 79, 5367–5381. 10.1158/0008-5472.CAN-19-0454 31439548

[B214] Moffat J. G. Rudolph J. Bailey D. (2014). Phenotypic screening in cancer drug discovery - past, present and future. Nat. Rev. Drug Discov. 13, 588–602. 10.1038/nrd4366 25033736

[B215] Moffat J. G. Vincent F. Lee J. A. Eder J. Prunotto M. (2017). Opportunities and challenges in phenotypic drug discovery: an industry perspective. Nat. Rev. Drug Discov. 16, 531–543. 10.1038/nrd.2017.111 28685762

[B216] Monticelli M. Liguori L. Allocca M. Bosso A. Andreotti G. Lukas J. (2022). Drug repositioning for Fabry disease: acetylsalicylic acid potentiates the stabilization of lysosomal alpha-galactosidase by pharmacological chaperones. Int. J. Mol. Sci. 23, 5105. 10.3390/ijms23095105 35563496 PMC9105905

[B217] Moore M. J. Goldstein D. Hamm J. Figer A. Hecht J. R. Gallinger S. (2007). Erlotinib plus gemcitabine compared with gemcitabine alone in patients with advanced pancreatic cancer: a phase III trial of the National Cancer Institute of Canada Clinical Trials Group. J. Clin. Oncol. 25, 1960–1966. 10.1200/JCO.2006.07.9525 17452677

[B218] Morgan S. Grootendorst P. Lexchin J. Cunningham C. Greyson D. (2011). The cost of drug development: a systematic review. Health Policy 100, 4–17. 10.1016/j.healthpol.2010.12.002 21256615

[B219] Morton C. L. Houghton P. J. (2007). Establishment of human tumor xenografts in immunodeficient mice. Nat. Protoc. 2, 247–250. 10.1038/nprot.2007.25 17406581

[B220] Mottini C. Napolitano F. Li Z. Gao X. Cardone L. (2021). Computer-aided drug repurposing for cancer therapy: approaches and opportunities to challenge anticancer targets. Semin. Cancer Biol. 68, 59–74. 10.1016/j.semcancer.2019.09.023 31562957

[B221] Mugiyanto E. Adikusuma W. Irham L. M. Huang W.-C. Chang W.-C. Kuo C.-N. (2022). Integrated genomic analysis to identify druggable targets for pancreatic cancer. Front. Oncol. 12, 989077. 10.3389/fonc.2022.989077 36531045 PMC9752886

[B222] Mühl H. Paulukat J. Höfler S. Hellmuth M. Franzen R. Pfeilschifter J. (2004). The HIV protease inhibitor ritonavir synergizes with butyrate for induction of apoptotic cell death and mediates expression of heme oxygenase-1 in DLD-1 colon carcinoma cells. Br. J. Pharmacol. 143, 890–898. 10.1038/sj.bjp.0706023 15504750 PMC1575947

[B223] Munoz L. (2017). Non-kinase targets of protein kinase inhibitors. Nat. Rev. Drug Discov. 16, 424–440. 10.1038/nrd.2016.266 28280261

[B224] Murphy J. E. Wo J. Y. Ryan D. P. Clark J. W. Jiang W. Yeap B. Y. (2019). Total neoadjuvant therapy with FOLFIRINOX in combination with losartan followed by chemoradiotherapy for locally advanced pancreatic cancer: a phase 2 clinical trial. JAMA Oncol. 5, 1020–1027. 10.1001/jamaoncol.2019.0892 31145418 PMC6547247

[B225] Mussin N. Oh S. C. Lee K. W. Park M. Y. Seo S. Yi N. J. (2017). Sirolimus and metformin synergistically inhibits colon cancer *in vitro* and *in vivo* . J. Korean Med. Sci. 32, 1385–1395. 10.3346/jkms.2017.32.9.1385 28776332 PMC5546956

[B226] Nagaraj A. B. Wang Q. Q. Joseph P. Zheng C. Chen Y. Kovalenko O. (2018). Using a novel computational drug-repositioning approach (DrugPredict) to rapidly identify potent drug candidates for cancer treatment. Oncogene 37, 403–414. 10.1038/onc.2017.328 28967908 PMC5799769

[B227] Nair V. Sreevalsan S. Basha R. Abdelrahim M. Abudayyeh A. Rodrigues Hoffman A. (2014). Mechanism of metformin-dependent inhibition of mammalian target of rapamycin (mTOR) and Ras activity in pancreatic cancer: role of specificity protein (Sp) transcription factors. J. Biol. Chem. 289, 27692–27701. 10.1074/jbc.M114.592576 25143389 PMC4183806

[B228] Nakayama G. Tanaka C. Kodera Y. (2013). Current options for the diagnosis, staging and therapeutic management of colorectal cancer. Gastrointest. Tumors 1, 25–32. 10.1159/000354995 26674429 PMC4645570

[B229] Nambara S. Masuda T. Nishio M. Kuramitsu S. Tobo T. Ogawa Y. (2017). Antitumor effects of the antiparasitic agent ivermectin via inhibition of Yes-associated protein 1 expression in gastric cancer. Oncotarget 8, 107666–107677. 10.18632/oncotarget.22587 29296196 PMC5746098

[B230] Neo J. H. Malcontenti-Wilson C. Muralidharan V. Christophi C. (2007). Effect of ACE inhibitors and angiotensin II receptor antagonists in a mouse model of colorectal cancer liver metastases. J. Gastroenterol. Hepatol. 22, 577–584. 10.1111/j.1440-1746.2006.04797.x 17376054

[B231] Nero T. L. Morton C. J. Holien J. K. Wielens J. Parker M. W. (2014). Oncogenic protein interfaces: small molecules, big challenges. Nat. Rev. Cancer 14, 248–262. 10.1038/nrc3690 24622521

[B232] Nuevo-Tapioles C. Santacatterina F. Stamatakis K. Núñez de Arenas C. Gómez de Cedrón M. Formentini L. (2020). Coordinate β-adrenergic inhibition of mitochondrial activity and angiogenesis arrest tumor growth. Nat. Commun. 11, 3606. 10.1038/s41467-020-17384-1 32681016 PMC7368041

[B233] Nygren P. Fryknäs M. Agerup B. Larsson R. (2013). Repositioning of the anthelmintic drug mebendazole for the treatment for colon cancer. J. Cancer Res. Clin. Oncol. 139, 2133–2140. 10.1007/s00432-013-1539-5 24135855 PMC3825534

[B234] Nygren P. Larsson R. (2014). Drug repositioning from bench to bedside: tumour remission by the antihelmintic drug mebendazole in refractory metastatic colon cancer. Acta Oncol. 53, 427–428. 10.3109/0284186X.2013.844359 24160353

[B235] Ogura S. Yoshida Y. Kurahashi T. Egawa M. Furuta K. Kiso S. (2018). Targeting the mevalonate pathway is a novel therapeutic approach to inhibit oncogenic FoxM1 transcription factor in human hepatocellular carcinoma. Oncotarget 9, 21022–21035. 10.18632/oncotarget.24781 29765517 PMC5940385

[B236] Okada J. Yamada E. Saito T. Yokoo H. Osaki A. Shimoda Y. (2020). Dapagliflozin inhibits cell adhesion to collagen I and IV and increases ectodomain proteolytic cleavage of DDR1 by increasing ADAM10 activity. Molecules 25, 495. 10.3390/molecules25030495 31979355 PMC7038111

[B237] Oliveira L. F. S. Predes D. Borges H. L. Abreu J. G. (2022). Therapeutic potential of naturally occurring small molecules to target the wnt/β-catenin signaling pathway in colorectal cancer. Cancers 14, 403. 10.3390/cancers14020403 35053565 PMC8774030

[B238] Onaciu A. Munteanu R. Munteanu V. C. Gulei D. Raduly L. Feder R.-I. (2020). Spontaneous and induced animal models for cancer research. Diagn. (Basel) 10, 660. 10.3390/diagnostics10090660 PMC7555044 32878340

[B239] Onoda T. Ono T. Dhar D. K. Yamanoi A. Fujii T. Nagasue N. (2004). Doxycycline inhibits cell proliferation and invasive potential: combination therapy with cyclooxygenase-2 inhibitor in human colorectal cancer cells. J. Lab. Clin. Med. 143, 207–216. 10.1016/j.lab.2003.12.012 15085079

[B240] Osada T. Chen M. Yang X. Y. Spasojevic I. Vandeusen J. B. Hsu D. (2011). Antihelminth compound niclosamide downregulates Wnt signaling and elicits antitumor responses in tumors with activating APC mutations. Cancer Res. 71, 4172–4182. 10.1158/0008-5472.CAN-10-3978 21531761 PMC3117125

[B241] Overman M. Javle M. Davis R. E. Vats P. Kumar-Sinha C. Xiao L. (2020). Randomized phase II study of the Bruton tyrosine kinase inhibitor acalabrutinib, alone or with pembrolizumab in patients with advanced pancreatic cancer. J. Immunother. Cancer 8, e000587. 10.1136/jitc-2020-000587 32114502 PMC7057435

[B242] Palve V. Liao Y. Remsing Rix L. L. Rix U. (2021). Turning liabilities into opportunities: off-target based drug repurposing in cancer. Semin. Cancer Biol. 68, 209–229. 10.1016/j.semcancer.2020.02.003 32044472 PMC7415607

[B243] Pantziarka P. Verbaanderd C. Sukhatme V. Rica Capistrano I. Crispino S. Gyawali B. (2018). ReDO_DB: the repurposing drugs in oncology database. Ecancermedicalscience 12, 886. 10.3332/ecancer.2018.886 30679953 PMC6345075

[B244] Parikh A. R. Leshchiner I. Elagina L. Goyal L. Levovitz C. Siravegna G. (2019). Liquid versus tissue biopsy for detecting acquired resistance and tumor heterogeneity in gastrointestinal cancers. Nat. Med. 25, 1415–1421. 10.1038/s41591-019-0561-9 31501609 PMC6741444

[B245] Park W. Amin A. R. M. R. Chen Z. G. Shin D. M. (2013). New perspectives of curcumin in cancer prevention. Cancer Prev. Res. 6, 387–400. 10.1158/1940-6207.CAPR-12-0410 PMC3693758 23466484

[B246] Patel M. M. Patel B. M. (2018). Repurposing of sodium valproate in colon cancer associated with diabetes mellitus: role of HDAC inhibition. Eur. J. Pharm. Sci. 121, 188–199. 10.1016/j.ejps.2018.05.026 29852291

[B247] Perrin J. Werner T. Kurzawa N. Rutkowska A. Childs D. D. Kalxdorf M. (2020). Identifying drug targets in tissues and whole blood with thermal-shift profiling. Nat. Biotechnol. 38, 303–308. 10.1038/s41587-019-0388-4 31959954

[B248] Perrone F. Zilbauer M. (2021). Biobanking of human gut organoids for translational research. Exp. Mol. Med. 53, 1451–1458. 10.1038/s12276-021-00606-x 34663935 PMC8569164

[B249] Petroni G. Bagni G. Iorio J. Duranti C. Lottini T. Stefanini M. (2020). Clarithromycin inhibits autophagy in colorectal cancer by regulating the hERG1 potassium channel interaction with PI3K. Cell Death Dis. 11, 161. 10.1038/s41419-020-2349-8 32123164 PMC7052256

[B250] Pham T.-H. Qiu Y. Liu J. Zimmer S. O’Neill E. Xie L. (2022). Chemical-induced gene expression ranking and its application to pancreatic cancer drug repurposing. Patterns (N Y) 3, 100441. 10.1016/j.patter.2022.100441 35465231 PMC9023899

[B251] Pintova S. Dharmupari S. Moshier E. Zubizarreta N. Ang C. Holcombe R. F. (2019). Genistein combined with FOLFOX or FOLFOX-Bevacizumab for the treatment of metastatic colorectal cancer: phase I/II pilot study. Cancer Chemother. Pharmacol. 84, 591–598. 10.1007/s00280-019-03886-3 31203390

[B252] Ponzini F. M. Schultz C. W. Leiby B. E. Cannaday S. Yeo T. Posey J. (2023). Repurposing the FDA-approved anthelmintic pyrvinium pamoate for pancreatic cancer treatment: study protocol for a phase I clinical trial in early-stage pancreatic ductal adenocarcinoma. BMJ Open 13, e073839. 10.1136/bmjopen-2023-073839 PMC10582846 37848297

[B253] Prasad K. Kumar V. (2021). Artificial intelligence-driven drug repurposing and structural biology for SARS-CoV-2. Curr. Res. Pharmacol. Drug Discov. 2, 100042. 10.1016/j.crphar.2021.100042 34870150 PMC8317454

[B254] Pritchard J.-L. E. O’Mara T. A. Glubb D. M. (2017). Enhancing the promise of drug repositioning through genetics. Front. Pharmacol. 8, 896. 10.3389/fphar.2017.00896 29270124 PMC5724196

[B255] Pushpakom S. Iorio F. Eyers P. A. Escott K. J. Hopper S. Wells A. (2019). Drug repurposing: progress, challenges and recommendations. Nat. Rev. Drug Discov. 18, 41–58. 10.1038/nrd.2018.168 30310233

[B256] Qiao X. Wang X. Shang Y. Li Y. Chen S.-Z. (2018). Azithromycin enhances anticancer activity of TRAIL by inhibiting autophagy and up-regulating the protein levels of DR4/5 in colon cancer cells *in vitro* and *in vivo* . Cancer Commun. 38, 43. 10.1186/s40880-018-0309-9 PMC6029027 29970185

[B257] Qorri B. Mokhtari R. B. Harless W. W. Szewczuk M. R. (2022). Next generation of cancer drug repurposing: therapeutic combination of aspirin and oseltamivir phosphate potentiates gemcitabine to disable key survival pathways critical for pancreatic cancer progression. Cancers 14, 1374. 10.3390/cancers14061374 35326525 PMC8946854

[B258] Rabben H.-L. Andersen G. T. Ianevski A. Olsen M. K. Kainov D. Grønbech J. E. (2021a). Computational drug repositioning and experimental validation of ivermectin in treatment of gastric cancer. Front. Pharmacol. 12, 625991. 10.3389/fphar.2021.625991 33867984 PMC8044519

[B259] Rabben H.-L. Kodama Y. Nakamura M. Bones A. M. Wang T. C. Chen D. (2021b). Chemopreventive effects of dietary isothiocyanates in animal models of gastric cancer and synergistic anticancer effects with cisplatin in human gastric cancer cells. Front. Pharmacol. 12, 613458. 10.3389/fphar.2021.613458 33897415 PMC8060630

[B260] Ramachandran S. Srivastava S. K. (2020). Repurposing pimavanserin, an anti-Parkinson drug for pancreatic cancer therapy. Mol. Ther. Oncolytics 19, 19–32. 10.1016/j.omto.2020.08.019 33024816 PMC7527685

[B261] Ranjan A. German N. Mikelis C. Srivenugopal K. Srivastava S. K. (2017). Penfluridol induces endoplasmic reticulum stress leading to autophagy in pancreatic cancer. Tumour Biol. 39, 1010428317705517. 10.1177/1010428317705517 28618969

[B262] Raziq K. Cai M. Dong K. Wang P. Afrifa J. Fu S. (2020). Competitive endogenous network of lncRNA, miRNA, and mRNA in the chemoresistance of gastrointestinal tract adenocarcinomas. Biomed. Pharmacother. 130, 110570. 10.1016/j.biopha.2020.110570 32763816

[B263] Rebelo R. Polónia B. Santos L. L. Vasconcelos M. H. Xavier C. P. R. (2021). Drug repurposing opportunities in pancreatic ductal adenocarcinoma. Pharmaceuticals 14, 280. 10.3390/ph14030280 33804613 PMC8003696

[B264] Regan-Fendt K. Li D. Reyes R. Yu L. Wani N. A. Hu P. (2020). Transcriptomics-based drug repurposing approach identifies novel drugs against sorafenib-resistant hepatocellular carcinoma. Cancers 12, 2730. 10.3390/cancers12102730 32977582 PMC7598246

[B265] Ribeiro E. Araújo D. Pereira M. Lopes B. Sousa P. Sousa A. C. (2023). Repurposing benztropine, natamycin, and nitazoxanide using drug combination and characterization of gastric cancer cell lines. Biomedicines 11, 799. 10.3390/biomedicines11030799 36979779 PMC10044866

[B266] Richmond A. Su Y. (2008). Mouse xenograft models vs GEM models for human cancer therapeutics. Dis. Model. Mech. 1, 78–82. 10.1242/dmm.000976 19048064 PMC2562196

[B267] Ridges S. Heaton W. L. Joshi D. Choi H. Eiring A. Batchelor L. (2012). Zebrafish screen identifies novel compound with selective toxicity against leukemia. Blood 119, 5621–5631. 10.1182/blood-2011-12-398818 22490804 PMC3382926

[B268] Rios Perez M. V. Roife D. Dai B. Pratt M. Dobrowolski R. Kang Y. (2019). Antineoplastic effects of auranofin in human pancreatic adenocarcinoma preclinical models. Surg. Open Sci. 1, 56–63. 10.1016/j.sopen.2019.05.004 33981979 PMC8083010

[B269] Ripani P. Delp J. Bode K. Delgado M. E. Dietrich L. Betzler V. M. (2020). Thiazolides promote G1 cell cycle arrest in colorectal cancer cells by targeting the mitochondrial respiratory chain. Oncogene 39, 2345–2357. 10.1038/s41388-019-1142-6 31844249

[B270] Rithanya P. Ezhilarasan D. (2021). Sodium valproate, a histone deacetylase inhibitor, provokes reactive oxygen species-mediated cytotoxicity in human hepatocellular carcinoma cells. J. Gastrointest. Cancer 52, 138–144. 10.1007/s12029-020-00370-7 32006341

[B271] Rose J. S. Bekaii-Saab T. S. (2011). New developments in the treatment of metastatic gastric cancer: focus on trastuzumab. Onco. Targets. Ther. 4, 21–26. 10.2147/OTT.S10188 21552412 PMC3084304

[B272] Rosenberg D. W. Giardina C. Tanaka T. (2009). Mouse models for the study of colon carcinogenesis. Carcinogenesis 30, 183–196. 10.1093/carcin/bgn267 19037092 PMC2639048

[B273] Ruggeri B. A. Camp F. Miknyoczki S. (2014). Animal models of disease: pre-clinical animal models of cancer and their applications and utility in drug discovery. Biochem. Pharmacol. 87, 150–161. 10.1016/j.bcp.2013.06.020 23817077

[B274] Sadeghi N. Abbruzzese J. L. Yeung S.-C. J. Hassan M. Li D. (2012). Metformin use is associated with better survival of diabetic patients with pancreatic cancer. Clin. Cancer Res. 18, 2905–2912. 10.1158/1078-0432.CCR-11-2994 22465831 PMC3381457

[B275] Sakamoto K. Maeda S. (2010). Targeting NF-kappaB for colorectal cancer. Expert Opin. Ther. Targets 14, 593–601. 10.1517/14728221003769903 20367537

[B276] Sala C. Dhar N. Hartkoorn R. C. Zhang M. Ha Y. H. Schneider P. (2010). Simple model for testing drugs against nonreplicating *Mycobacterium tuberculosis* . Mycobacterium Tuberc. Antimicrob. Agents Chemother. 54, 4150–4158. 10.1128/AAC.00821-10 PMC2944619 20679505

[B277] Samaras P. Tusup M. Nguyen-Kim T. D. L. Seifert B. Bachmann H. von Moos R. (2017). Phase I study of a chloroquine-gemcitabine combination in patients with metastatic or unresectable pancreatic cancer. Cancer Chemother. Pharmacol. 80, 1005–1012. 10.1007/s00280-017-3446-y 28980060

[B278] Sanomachi T. Suzuki S. Kuramoto K. Takeda H. Sakaki H. Togashi K. (2017). Olanzapine, an atypical antipsychotic, inhibits survivin expression and sensitizes cancer cells to chemotherapeutic agents. Anticancer Res. 37, 6177–6188. 10.21873/anticanres.12067 29061799

[B279] Sanseau P. Agarwal P. Barnes M. R. Pastinen T. Richards J. B. Cardon L. R. (2012). Use of genome-wide association studies for drug repositioning. Nat. Biotechnol. 30, 317–320. 10.1038/nbt.2151 22491277

[B280] Sarfstein R. Friedman Y. Attias-Geva Z. Fishman A. Bruchim I. Werner H. (2013). Metformin downregulates the insulin/IGF-I signaling pathway and inhibits different uterine serous carcinoma (USC) cells proliferation and migration in p53-dependent or -independent manners. PLoS One 8, e61537. 10.1371/journal.pone.0061537 23620761 PMC3631250

[B281] Savitski M. M. Zinn N. Faelth-Savitski M. Poeckel D. Gade S. Becher I. (2018). Multiplexed proteome dynamics profiling reveals mechanisms controlling protein homeostasis. Cell 173, 260–274. 10.1016/j.cell.2018.02.030 29551266 PMC5871718

[B282] Schultz C. W. McCarthy G. A. Nerwal T. Nevler A. DuHadaway J. B. McCoy M. D. (2021). The FDA-approved anthelmintic pyrvinium pamoate inhibits pancreatic cancer cells in nutrient-depleted conditions by targeting the mitochondria. Mol. Cancer Ther. 20, 2166–2176. 10.1158/1535-7163.MCT-20-0652 34413127 PMC8859979

[B283] Senkowski W. Zhang X. Olofsson M. H. Isacson R. Höglund U. Gustafsson M. (2015). Three-dimensional cell culture-based screening identifies the anthelmintic drug nitazoxanide as a candidate for treatment of colorectal cancer. Mol. Cancer Ther. 14, 1504–1516. 10.1158/1535-7163.MCT-14-0792 25911689

[B284] Shafiee G. Saidijam M. Tavilani H. Ghasemkhani N. Khodadadi I. (2016). Genistein induces apoptosis and inhibits proliferation of HT29 colon cancer cells. Int. J. Mol. Cell Med. 5, 178–191. 27942504 PMC5125370

[B285] Shameer K. Readhead B. Dudley J. T. (2015). Computational and experimental advances in drug repositioning for accelerated therapeutic stratification. Curr. Top. Med. Chem. 15, 5–20. 10.2174/1568026615666150112103510 25579574

[B286] Shantikumar S. Satheeshkumar N. Srinivas R. (2015). Pharmacokinetic and protein binding profile of peptidomimetic DPP-4 inhibitor - teneligliptin in rats using liquid chromatography-tandem mass spectrometry. J. Chromatogr. B Anal. Technol. Biomed. Life Sci. 1002, 194–200. 10.1016/j.jchromb.2015.08.023 26340762

[B287] Shen P.-W. Chou Y.-M. Li C.-L. Liao E.-C. Huang H.-S. Yin C.-H. (2021). Itraconazole improves survival outcomes in patients with colon cancer by inducing autophagic cell death and inhibiting transketolase expression. Oncol. Lett. 22, 768. 10.3892/ol.2021.13029 34589147 PMC8442143

[B288] Sherif D. A. Makled M. N. Suddek G. M. (2021). The HIV reverse transcriptase Inhibitor Tenofovir suppressed DMH/HFD-induced colorectal cancer in Wistar rats. Fundam. Clin. Pharmacol. 35, 940–954. 10.1111/fcp.12679 33829539

[B289] Shi J. Zhou L. Huang H.-S. Peng L. Xie N. Nice E. (2022a). Repurposing oxiconazole against colorectal cancer via PRDX2-mediated autophagy arrest. Int. J. Biol. Sci. 18, 3747–3761. 10.7150/ijbs.70679 35813474 PMC9254464

[B290] Shi W. Li C. Wartmann T. Kahlert C. Du R. Perrakis A. (2022b). Sensory ion channel candidates inform on the clinical course of pancreatic cancer and present potential targets for repurposing of FDA-approved agents. J. Pers. Med. 12, 478. 10.3390/jpm12030478 35330477 PMC8950951

[B291] Shi X.-N. Li H. Yao H. Liu X. Li L. Leung K.-S. (2015). Adapalene inhibits the activity of cyclin-dependent kinase 2 in colorectal carcinoma. Mol. Med. Rep. 12, 6501–6508. 10.3892/mmr.2015.4310 26398439 PMC4626183

[B292] Shitara K. Doi T. Nagano O. Imamura C. K. Ozeki T. Ishii Y. (2017). Dose-escalation study for the targeting of CD44v+ cancer stem cells by sulfasalazine in patients with advanced gastric cancer (EPOC1205). Gastric Cancer 20, 341–349. 10.1007/s10120-016-0610-8 27055559

[B293] Simian M. Bissell M. J. (2017). Organoids: a historical perspective of thinking in three dimensions. J. Cell Biol. 216, 31–40. 10.1083/jcb.201610056 28031422 PMC5223613

[B294] Sinn D. H. Choi G.-S. Park H. C. Kim J. M. Kim H. Song K. D. (2019). Multidisciplinary approach is associated with improved survival of hepatocellular carcinoma patients. PLoS One 14, e0210730. 10.1371/journal.pone.0210730 30640924 PMC6331107

[B295] Sivaraman A. Leach J. K. Townsend S. Iida T. Hogan B. J. Stolz D. B. (2005). A microscale *in vitro* physiological model of the liver: predictive screens for drug metabolism and enzyme induction. Curr. Drug Metab. 6, 569–591. 10.2174/138920005774832632 16379670

[B296] Sloane D. A. Trikic M. Z. Chu M. L. H. Lamers M. B. A. C. Mason C. S. Mueller I. (2010). Drug-resistant aurora A mutants for cellular target validation of the small molecule kinase inhibitors MLN8054 and MLN8237. ACS Chem. Biol. 5, 563–576. 10.1021/cb100053q 20426425

[B297] Smith S. B. Dampier W. Tozeren A. Brown J. R. Magid-Slav M. (2012). Identification of common biological pathways and drug targets across multiple respiratory viruses based on human host gene expression analysis. PLoS One 7, e33174. 10.1371/journal.pone.0033174 22432004 PMC3303816

[B298] Sobko T. Reinders C. I. Jansson E. Norin E. Midtvedt T. Lundberg J. O. (2005). Gastrointestinal bacteria generate nitric oxide from nitrate and nitrite. Nitric Oxide 13, 272–278. 10.1016/j.niox.2005.08.002 16183308

[B299] Son D.-S. Lee E.-S. Adunyah S. E. (2020). The antitumor potentials of benzimidazole anthelmintics as repurposing drugs. Immune Netw. 20, e29. 10.4110/in.2020.20.e29 32895616 PMC7458798

[B300] Son K. Fujioka S. Iida T. Furukawa K. Fujita T. Yamada H. (2009). Doxycycline induces apoptosis in PANC-1 pancreatic cancer cells. Anticancer Res. 29, 3995–4003. 19846942

[B301] Stastna M. Janeckova L. Hrckulak D. Kriz V. Korinek V. (2019). Human colorectal cancer from the perspective of mouse models. Genes 10, 788. 10.3390/genes10100788 31614493 PMC6826908

[B302] Stewart A. K. Jacobus S. Fonseca R. Weiss M. Callander N. S. Chanan-Khan A. A. (2015). Melphalan, prednisone, and thalidomide vs melphalan, prednisone, and lenalidomide (ECOG E1A06) in untreated multiple myeloma. Blood 126, 1294–1301. 10.1182/blood-2014-12-613927 26157076 PMC4566809

[B303] Streicher S. A. Yu H. Lu L. Kidd M. S. Risch H. A. (2014). Case-control study of aspirin use and risk of pancreatic cancer. Cancer Epidemiol. Biomarkers Prev. 23, 1254–1263. 10.1158/1055-9965.EPI-13-1284 24969230 PMC4091763

[B304] Sud A. Kinnersley B. Houlston R. S. (2017). Genome-wide association studies of cancer: current insights and future perspectives. Nat. Rev. Cancer 17, 692–704. 10.1038/nrc.2017.82 29026206

[B305] Sugarbaker P. H. (2005). Strategies for the prevention and treatment of peritoneal carcinomatosis from gastrointestinal cancer. Cancer Invest. 23, 155–172. 10.1081/cnv-50478 15813509

[B306] Sun W. Weingarten R. A. Xu M. Southall N. Dai S. Shinn P. (2016). Rapid antimicrobial susceptibility test for identification of new therapeutics and drug combinations against multidrug-resistant bacteria. Emerg. Microbes Infect. 5, e116. 10.1038/emi.2016.123 27826141 PMC5148025

[B307] Sun X. Sun G. Huang Y. Hao Y. Tang X. Zhang N. (2020). 3-Bromopyruvate regulates the status of glycolysis and BCNU sensitivity in human hepatocellular carcinoma cells. Biochem. Pharmacol. 177, 113988. 10.1016/j.bcp.2020.113988 32330495

[B308] Sung H. Ferlay J. Siegel R. L. Laversanne M. Soerjomataram I. Jemal A. (2021). Global cancer statistics 2020: GLOBOCAN estimates of incidence and mortality worldwide for 36 cancers in 185 countries. CA Cancer J. Clin. 71, 209–249. 10.3322/caac.21660 33538338

[B309] Tabatabai E. Khazaei M. Asgharzadeh F. Nazari S. E. Shakour N. Fiuji H. (2021). Inhibition of angiotensin II type 1 receptor by candesartan reduces tumor growth and ameliorates fibrosis in colorectal cancer. EXCLI J. 20, 863–878. 10.17179/excli2021-3421 34121975 PMC8192880

[B310] Takahashi M. Kobayashi H. Mizutani Y. Hara A. Iida T. Miyai Y. (2021). Roles of the mesenchymal stromal/stem cell marker meflin/islr in cancer fibrosis. Front. Cell Dev. Biol. 9, 749924. 10.3389/fcell.2021.749924 34676218 PMC8523999

[B311] Tan X.-P. He Y. Yang J. Wei X. Fan Y.-L. Zhang G.-G. (2023). Blockade of NMT1 enzymatic activity inhibits N-myristoylation of VILIP3 protein and suppresses liver cancer progression. Signal Transduct. Target Ther. 8, 14. 10.1038/s41392-022-01248-9 36617552 PMC9826789

[B312] Teixeira Farinha H. Digklia A. Schizas D. Demartines N. Schäfer M. Mantziari S. (2022). Immunotherapy for esophageal cancer: state-of-the art in 2021. Cancers 14, 554. 10.3390/cancers14030554 35158822 PMC8833794

[B313] Tentler J. J. Tan A. C. Weekes C. D. Jimeno A. Leong S. Pitts T. M. (2012). Patient-derived tumour xenografts as models for oncology drug development. Nat. Rev. Clin. Oncol. 9, 338–350. 10.1038/nrclinonc.2012.61 22508028 PMC3928688

[B314] Tomi-Andrino C. Pandele A. Winzer K. King J. Rahman R. Kim D.-H. (2022). Metabolic modeling-based drug repurposing in Glioblastoma. Sci. Rep. 12, 11189. 10.1038/s41598-022-14721-w 35778411 PMC9249780

[B315] Tomita H. Takaishi S. Menheniott T. R. Yang X. Shibata W. Jin G. (2011). Inhibition of gastric carcinogenesis by the hormone gastrin is mediated by suppression of TFF1 epigenetic silencing. Gastroenterology 140, 879–891. 10.1053/j.gastro.2010.11.037 21111741 PMC3049860

[B316] Toschi E. Sgadari C. Malavasi L. Bacigalupo I. Chiozzini C. Carlei D. (2011). Human immunodeficiency virus protease inhibitors reduce the growth of human tumors via a proteasome-independent block of angiogenesis and matrix metalloproteinases. Int. J. Cancer 128, 82–93. 10.1002/ijc.25550 20617515

[B317] Tseng C.-N. Huang C.-F. Cho C.-L. Chang H.-W. Huang C.-W. Chiu C.-C. (2013). Brefeldin a effectively inhibits cancer stem cell-like properties and MMP-9 activity in human colorectal cancer Colo 205 cells. Molecules 18, 10242–10253. 10.3390/molecules180910242 23973996 PMC6270264

[B318] Vacante F. Senesi P. Montesano A. Paini S. Luzi L. Terruzzi I. (2019). Metformin counteracts HCC progression and metastasis enhancing KLF6/p21 expression and downregulating the IGF Axis. Int. J. Endocrinol. 2019, 7570146. 10.1155/2019/7570146 30774659 PMC6350585

[B319] Valastyan S. Weinberg R. A. (2011). Tumor metastasis: molecular insights and evolving paradigms. Cell 147, 275–292. 10.1016/j.cell.2011.09.024 22000009 PMC3261217

[B320] van de Wetering M. Francies H. E. Francis J. M. Bounova G. Iorio F. Pronk A. (2015). Prospective derivation of a living organoid biobank of colorectal cancer patients. Cell 161, 933–945. 10.1016/j.cell.2015.03.053 25957691 PMC6428276

[B321] Verduin M. Hoeben A. De Ruysscher D. Vooijs M. (2021). Patient-derived cancer organoids as predictors of treatment response. Front. Oncol. 11, 641980. 10.3389/fonc.2021.641980 33816288 PMC8012903

[B322] Veschi S. De Lellis L. Florio R. Lanuti P. Massucci A. Tinari N. (2018). Effects of repurposed drug candidates nitroxoline and nelfinavir as single agents or in combination with erlotinib in pancreatic cancer cells. J. Exp. Clin. Cancer Res. 37, 236. 10.1186/s13046-018-0904-2 30241558 PMC6151049

[B323] Veschi S. Ronci M. Lanuti P. De Lellis L. Florio R. Bologna G. (2020). Integrative proteomic and functional analyses provide novel insights into the action of the repurposed drug candidate nitroxoline in AsPC-1 cells. Sci. Rep. 10, 2574. 10.1038/s41598-020-59492-4 32054977 PMC7018951

[B324] Vickers N. J. (2017). Animal communication: when I’m calling you, will you answer too? Curr. Biol. 27, R713–R715. 10.1016/j.cub.2017.05.064 28743020

[B325] Villarruel-Melquiades F. Hernandez-Gallegos E. Solano-Agama C. Mendoza-Garrido M. E. Camacho J. (2023). The combination sorafenib-raloxifene-loratadine as a novel potential therapeutic approach against human liver cancer. Vivo 37, 1156–1163. 10.21873/invivo.13190 PMC10188024 37103074

[B326] Walrath J. C. Hawes J. J. Van Dyke T. Reilly K. M. (2010). Genetically engineered mouse models in cancer research. Adv. Cancer Res. 106, 113–164. 10.1016/S0065-230X(10)06004-5 20399958 PMC3533445

[B327] Wang J. Ren X.-R. Piao H. Zhao S. Osada T. Premont R. T. (2019). Niclosamide-induced Wnt signaling inhibition in colorectal cancer is mediated by autophagy. Biochem. J. 476, 535–546. 10.1042/BCJ20180385 30635359 PMC6643999

[B328] Wang S.-T. Ho H. J. Lin J.-T. Shieh J.-J. Wu C.-Y. (2017). Simvastatin-induced cell cycle arrest through inhibition of STAT3/SKP2 axis and activation of AMPK to promote p27 and p21 accumulation in hepatocellular carcinoma cells. Cell Death Dis. 8, e2626. 10.1038/cddis.2016.472 28230855 PMC5386458

[B329] Wang W. McLeod H. L. Cassidy J. (2003). Disulfiram-mediated inhibition of NF-kappaB activity enhances cytotoxicity of 5-fluorouracil in human colorectal cancer cell lines. Int. J. Cancer 104, 504–511. 10.1002/ijc.10972 12584750

[B330] Wang X. Wu X. Zhang Z. Ma C. Wu T. Tang S. (2018a). Monensin inhibits cell proliferation and tumor growth of chemo-resistant pancreatic cancer cells by targeting the EGFR signaling pathway. Sci. Rep. 8, 17914. 10.1038/s41598-018-36214-5 30559409 PMC6297164

[B331] Wang Y. Xu W. Yan Z. Zhao W. Mi J. Li J. (2018b). Metformin induces autophagy and G0/G1 phase cell cycle arrest in myeloma by targeting the AMPK/mTORC1 and mTORC2 pathways. J. Exp. Clin. Cancer Res. 37, 63. 10.1186/s13046-018-0731-5 29554968 PMC5859411

[B332] Wang Y.-S. Huang N.-K. Lin Y.-C. Chang W.-C. Huang W.-C. (2022). Aspirin and Sulindac act via different mechanisms to inhibit store-operated calcium channel: implications for colorectal cancer metastasis. Biomed. Pharmacother. 145, 112476. 10.1016/j.biopha.2021.112476 34864310

[B333] Wang Z.-Y. Zhang H.-Y. (2013). Rational drug repositioning by medical genetics. Nat. Biotechnol. 31, 1080–1082. 10.1038/nbt.2758 24316641

[B334] Wei L. Sun J. Zhang N. Shen Y. Wang T. Li Z. (2021). Novel implications of MicroRNAs, long non-coding RNAs and circular RNAs in drug resistance of esophageal cancer. Front. Cell Dev. Biol. 9, 764313. 10.3389/fcell.2021.764313 34881242 PMC8645845

[B335] Wei W.-Q. Mosley J. D. Bastarache L. Denny J. C. (2013). Validation and enhancement of a computable medication indication resource (MEDI) using a large practice-based dataset. AMIA Annu. Symp. Proc. 2013, 1448–1456. 24551419 PMC3900157

[B336] Westphalen C. B. Olive K. P. (2012). Genetically engineered mouse models of pancreatic cancer. Cancer J. 18, 502–510. 10.1097/PPO.0b013e31827ab4c4 23187836 PMC3594661

[B337] Wilkinson G. F. Pritchard K. (2015). *In vitro* screening for drug repositioning. J. Biomol. Screen. 20, 167–179. 10.1177/1087057114563024 25527136

[B338] Willyard C. (2018). New human gene tally reignites debate. Nature 558, 354–355. 10.1038/d41586-018-05462-w 29921859

[B339] Wong L. H. Sinha S. Bergeron J. R. Mellor J. C. Giaever G. Flaherty P. (2016). Reverse chemical genetics: comprehensive fitness profiling reveals the spectrum of drug target interactions. PLoS Genet. 12, e1006275. 10.1371/journal.pgen.1006275 27588687 PMC5010250

[B340] Wu M. Swartz M. A. (2014). Modeling tumor microenvironments *in vitro* . J. Biomech. Eng. 136, 021011. 10.1115/1.4026447 24402507 PMC4023667

[B341] Wu X. Cao Y. Xiao H. Li C. Lin J. (2016). Bazedoxifene as a novel GP130 inhibitor for pancreatic cancer therapy. Mol. Cancer Ther. 15, 2609–2619. 10.1158/1535-7163.MCT-15-0921 27535971 PMC5310670

[B342] Würth R. Thellung S. Bajetto A. Mazzanti M. Florio T. Barbieri F. (2016). Drug-repositioning opportunities for cancer therapy: novel molecular targets for known compounds. Drug Discov. Today 21, 190–199. 10.1016/j.drudis.2015.09.017 26456577

[B343] Xavier C. P. R. Castro I. Caires H. R. Ferreira D. Cavadas B. Pereira L. (2021). Chitinase 3-like-1 and fibronectin in the cargo of extracellular vesicles shed by human macrophages influence pancreatic cancer cellular response to gemcitabine. Cancer Lett. 501, 210–223. 10.1016/j.canlet.2020.11.013 33212158

[B344] Xiao Y. Liu Q. Peng N. Li Y. Qiu D. Yang T. (2022). Lovastatin inhibits RhoA to suppress canonical wnt/β-catenin signaling and alternative wnt-YAP/TAZ signaling in colon cancer. Cell Transpl. 31, 9636897221075749. 10.1177/09636897221075749 PMC8855423 35168393

[B345] Xie H. Qiang P. Wang Y. Xia F. Liu P. Li M. (2022). Discovery and mechanism studies of a novel ATG4B inhibitor Ebselen by drug repurposing and its anti-colorectal cancer effects in mice. Cell Biosci. 12, 206. 10.1186/s13578-022-00944-x 36539845 PMC9767854

[B346] Xie J. Xia L. Xiang W. He W. Yin H. Wang F. (2020). Metformin selectively inhibits metastatic colorectal cancer with the KRAS mutation by intracellular accumulation through silencing MATE1. Proc. Natl. Acad. Sci. U. S. A. 117, 13012–13022. 10.1073/pnas.1918845117 32444490 PMC7293710

[B347] Xing Y.-X. Li M.-H. Tao L. Ruan L.-Y. Hong W. Chen C. (2018). Anti-cancer effects of emodin on HepG2 cells as revealed by 1H NMR based metabolic profiling. J. Proteome Res. 17, 1943–1952. 10.1021/acs.jproteome.8b00029 29676152

[B348] Xiong L. Lou Y. Wang L. (2021). Effect of bevacizumab combined with first-line chemotherapy on metastatic colorectal cancer. Am. J. Transl. Res. 13, 3609–3617. 34017542 PMC8129318

[B349] Xiong T. Li Z. Huang X. Lu K. Xie W. Zhou Z. (2019). TO901317 inhibits the development of hepatocellular carcinoma by LXRα/Glut1 decreasing glycometabolism. Am. J. Physiol. Gastrointest. Liver Physiol. 316, G598–G607. 10.1152/ajpgi.00061.2018 30817182

[B350] Xiong W. Li W.-H. Jiang Y.-X. Liu S. Ai Y.-Q. Liu R. (2015). Parecoxib: an enhancer of radiation therapy for colorectal cancer. Asian pac. J. Cancer Prev. 16, 627–633. 10.7314/apjcp.2015.16.2.627 25684498

[B351] Xu D. Jin J. Yu H. Zhao Z. Ma D. Zhang C. (2017). Chrysin inhibited tumor glycolysis and induced apoptosis in hepatocellular carcinoma by targeting hexokinase-2. J. Exp. Clin. Cancer Res. 36, 44. 10.1186/s13046-017-0514-4 28320429 PMC5359903

[B352] Xu H. Aldrich M. C. Chen Q. Liu H. Peterson N. B. Dai Q. (2015). Validating drug repurposing signals using electronic health records: a case study of metformin associated with reduced cancer mortality. J. Am. Med. Inf. Assoc. 22, 179–191. 10.1136/amiajnl-2014-002649 PMC4433365 25053577

[B353] Xu M. Lee E. M. Wen Z. Cheng Y. Huang W.-K. Qian X. (2016). Identification of small-molecule inhibitors of Zika virus infection and induced neural cell death via a drug repurposing screen. Nat. Med. 22, 1101–1107. 10.1038/nm.4184 27571349 PMC5386783

[B354] Xu X. Wang J. Han K. Li S. Xu F. Yang Y. (2018). Antimalarial drug mefloquine inhibits nuclear factor kappa B signaling and induces apoptosis in colorectal cancer cells. Cancer Sci. 109, 1220–1229. 10.1111/cas.13540 29453896 PMC5891192

[B355] Yadav A. K. Srikrishna S. Gupta S. C. (2016). Cancer drug development using Drosophila as an *in vivo* tool: from bedside to bench and back. Trends Pharmacol. Sci. 37, 789–806. 10.1016/j.tips.2016.05.010 27298020

[B356] Yamasaki D. Kawabe N. Nakamura H. Tachibana K. Ishimoto K. Tanaka T. (2011). Fenofibrate suppresses growth of the human hepatocellular carcinoma cell via PPARα-independent mechanisms. Eur. J. Cell Biol. 90, 657–664. 10.1016/j.ejcb.2011.02.005 21514001

[B357] Yang C. Zhang H. Chen M. Wang S. Qian R. Zhang L. (2022). A survey of optimal strategy for signature-based drug repositioning and an application to liver cancer. Elife 11, e71880. 10.7554/eLife.71880 35191375 PMC8893721

[B358] Yang J. Jin X. Yan Y. Shao Y. Pan Y. Roberts L. R. (2017). Inhibiting histone deacetylases suppresses glucose metabolism and hepatocellular carcinoma growth by restoring FBP1 expression. Sci. Rep. 7, 43864. 10.1038/srep43864 28262837 PMC5338333

[B359] Yu M. Tong X. Qi B. Qu H. Dong S. Yu B. (2014). Berberine enhances chemosensitivity to irinotecan in colon cancer via inhibition of NF-κB. Mol. Med. Rep. 9, 249–254. 10.3892/mmr.2013.1762 24173769

[B360] Yu Q.-S. Xin H.-R. Qiu R.-L. Deng Z.-L. Deng F. Yan Z.-J. (2020). Niclosamide: drug repurposing for human chondrosarcoma treatment via the caspase-dependent mitochondrial apoptotic pathway. Am. J. Transl. Res. 12, 3688–3701. 32774727 PMC7407720

[B361] Yuan M. Shong K. Li X. Ashraf S. Shi M. Kim W. (2022). A gene Co-expression network-based drug repositioning approach identifies candidates for treatment of hepatocellular carcinoma. Cancers 14, 1573. 10.3390/cancers14061573 35326724 PMC8946504

[B362] Yumimoto K. Sugiyama S. Mimori K. Nakayama K. I. (2019). Potentials of C-C motif chemokine 2-C-C chemokine receptor type 2 blockers including propagermanium as anticancer agents. Cancer Sci. 110, 2090–2099. 10.1111/cas.14075 31111571 PMC6609805

[B363] Zamami Y. Imanishi M. Takechi K. Ishizawa K. (2017). Pharmacological approach for drug repositioning against cardiorenal diseases. J. Med. Invest. 64, 197–201. 10.2152/jmi.64.197 28954981

[B364] Zhang D. Ma Q. Shen S. Hu H. (2009). Inhibition of pancreatic cancer cell proliferation by propranolol occurs through apoptosis induction: the study of beta-adrenoceptor antagonist’s anticancer effect in pancreatic cancer cell. Pancreas 38, 94–100. 10.1097/MPA.0b013e318184f50c 19106745

[B365] Zhang D. Ma Q.-Y. Hu H.-T. Zhang M. (2010). β2-adrenergic antagonists suppress pancreatic cancer cell invasion by inhibiting CREB, NFκB and AP-1. Cancer Biol. Ther. 10, 19–29. 10.4161/cbt.10.1.11944 20424515

[B366] Zhang J. Gao M. Niu Y. Sun J. (2022a). From DNMT1 degrader to ferroptosis promoter: drug repositioning of 6-Thioguanine as a ferroptosis inducer in gastric cancer. Biochem. Biophys. Res. Commun. 603, 75–81. 10.1016/j.bbrc.2022.03.026 35278883

[B367] Zhang J. Gao M. Niu Y. Sun J. (2022b). Identification of a novel ferroptosis inducer for gastric cancer treatment using drug repurposing strategy. Front. Mol. Biosci. 9, 860525. 10.3389/fmolb.2022.860525 35860356 PMC9289365

[B368] Zhang J. Jiang K. Lv L. Wang H. Shen Z. Gao Z. (2015). Use of genome-wide association studies for cancer research and drug repositioning. PLoS One 10, e0116477. 10.1371/journal.pone.0116477 25803826 PMC4372357

[B369] Zhang X. Hu P. Ding S.-Y. Sun T. Liu L. Han S. (2019). Induction of autophagy-dependent apoptosis in cancer cells through activation of ER stress: an uncovered anti-cancer mechanism by anti-alcoholism drug disulfiram. Am. J. Cancer Res. 9, 1266–1281. 31285958 PMC6610050

[B370] Zhang X. Luo H. (2018). Effects of thalidomide on growth and VEGF-A expression in SW480 colon cancer cells. Oncol. Lett. 15, 3313–3320. 10.3892/ol.2017.7645 29435073 PMC5778822

[B371] Zhang X. Wu T. Cai X. Dong J. Xia C. Zhou Y. (2022c). Neoadjuvant immunotherapy for MSI-H/dMMR locally advanced colorectal cancer: new strategies and unveiled opportunities. Front. Immunol. 13, 795972. 10.3389/fimmu.2022.795972 35371084 PMC8968082

[B372] Zhao P. Shen Y. Li M. Dan H. Zhao Z. Zhang J. (2022). Integration of transcriptomics and metabolomics reveals the antitumor mechanism underlying tadalafil in colorectal cancer. Front. Pharmacol. 13, 793499. 10.3389/fphar.2022.793499 35694253 PMC9184725

[B373] Zhao Y. He M. Liang R. Li Q. Shi M. (2021). Evaluation of antiemetic therapy for hepatic arterial infusion chemotherapy with oxaliplatin, fluorouracil, and leucovorin. Ther. Clin. Risk Manag. 17, 73–77. 10.2147/TCRM.S283192 33519205 PMC7837558

[B374] Zheng H.-C. (2017). The molecular mechanisms of chemoresistance in cancers. Oncotarget 8, 59950–59964. 10.18632/oncotarget.19048 28938696 PMC5601792

[B375] Zheng S. Wu Y. X. Wang J. Y. Li Y. Liu Z. J. Liu X. G. (2020). Identifying the characteristics of patients with cervical degenerative disease for surgical treatment from 17-year real-world data: retrospective study. JMIR Med. Inf. 8, e16076. 10.2196/16076 PMC7165306 32242824

[B376] Zhou L. Gao W. Wang K. Huang Z. Zhang L. Zhang Z. (2019a). Brefeldin A inhibits colorectal cancer growth by triggering Bip/Akt-regulated autophagy. FASEB J. 33, 5520–5534. 10.1096/fj.201801983R 30668917

[B377] Zhou S. Wu H. Ning W. Wu X. Xu X. Ma Y. (2021). Ivermectin has new application in inhibiting colorectal cancer cell growth. Front. Pharmacol. 12, 717529. 10.3389/fphar.2021.717529 34483925 PMC8415024

[B378] Zhou Y. Zhou Y. Yang M. Wang K. Liu Y. Zhang M. (2019b). Digoxin sensitizes gemcitabine-resistant pancreatic cancer cells to gemcitabine via inhibiting Nrf2 signaling pathway. Redox Biol. 22, 101131. 10.1016/j.redox.2019.101131 30735911 PMC6365940

[B379] Zhuang S. Jian Y.-M. Sun Y.-N. (2017). Inhibition of N-methyl-N-nitrosourea-induced gastric tumorigenesis by Liuwei Dihuang Pill in db/db mice. World J. Gastroenterol. 23, 4233–4242. 10.3748/wjg.v23.i23.4233 28694663 PMC5483497

[B380] Zweegman S. van der Holt B. Mellqvist U.-H. Salomo M. Bos G. M. J. Levin M.-D. (2016). Melphalan, prednisone, and lenalidomide versus melphalan, prednisone, and thalidomide in untreated multiple myeloma. Blood 127, 1109–1116. 10.1182/blood-2015-11-679415 26802176

