# Supplementary material for: Current trends and future prospects of drug repositioning in gastrointestinal oncology
Source: Front Pharmacol. 2024 Jan 4;14:1329244. doi: 10.3389/fphar.2023.1329244 (PMC10794567; doi:10.3389/fphar.2023.1329244)
Supplement: Supplementary file 1 [file Table1.DOCX]

**Table S1:** GI cancers therapies using medication

| **Cancer type** | **Chemotherapy** | **Targeted therapy** | **Immunotherapy** | **Combined therapy** |
| --- | --- | --- | --- | --- |
| **Pancreatic Cancer** | - Capecitabine - Fluorouracil - Gemcitabine - Irinotecan - Leucovorin - Nab-paclitaxel - Nanoliposomal irinotecan - Oxaliplatin | Cancer with an NTRK fusion:   - Larotrectinib - Entrectinib   BRCA1 or BRCA2 mutation:   - Olaparib   Anti-EGFR:   - Erlotinib | Anti-PD-1 antibodies for treating (MSI-H) or (dMMR) PC:   - Pembrolizumab - Dostarlimab | Metastatic PC:   - Combination of fluorouracil, leucovorin, irinotecan, and oxaliplatin, called FOLFIRINOX - Gemcitabine plus nab-paclitaxel - gemcitabine plus cisplatin, gemcitabine plus capecitabine, or FOLFOX. - Fluorouracil, nanoliposomal irinotecan, irinotecan - Gemcitabine, nab-paclitaxel |
| **Colorectal Cancer** | - Capecitabine - Fluorouracil - Irinotecan - Oxaliplatin - Trifluridine/tipiracil | Anti-angiogenesis therapy:   - Bevacizumab - Regorafenib - Ziv-aflibercept - Ramucirumab   Anti-EGFR:   - Cetuximab - Panitumumab   BRAF inhibitor:   - Encorafenib   Cancer with an NTRK fusion:   - Larotrectinib - Entrectinib | Anti-PD-1 antibodies:   - Dostarlimab (Recurrent or metastatic colorectal cancers that have dMMR) - Pembrolizumab (fro (MSI-H) or (dMMR) CRC) - Nivolumab (Metastatic (MSI-H) or (dMMR) CRC)   Combination of immune checkpoint inhibitors:   - Nivolumab and ipilimumab (Metastatic (MSI-H) or (dMMR) CRC) | Combined targeted therapy for BRAF V600E:   - Encorafenib and Cetuximab   Common combined therapies:   - 5-FU with leucovorin - FOLFOX: 5-FU with leucovorin and oxaliplatin - 5-FU with leucovorin and irinotecan - Capecitabine with irinotecan   Capecitabine with oxaliplatin |
| **Liver Cancer** |  | Anti-angiogenesis therapy:   - Regorafenib - Ramucirumab - Bevacizumab - Ramucirumab   Tyrosine kinase inhibitors   - Lenvatinib - Sorafenib - Cabozantinib | Immune checkpoint inhibitors:   - Pembrolizumab - Nivolumab - Ipilimumab | First-line treatment:   - Bevacizumab with atezolizumab (advanced HCC) - Sorafenib or lenvatinib with bevacizumab   Second-line treatment for advanced HCC):   - Atezolizumab and bevacizumab |
| **Esophageal Cancer** | - Paclitaxel | HER2-targeted therapy:   - Trastuzumab - Trastuzumab deruxtecan   Anti-angiogenesis therapy:   - Ramucirumab | Anti-PD-1 antibodies:   - Pembrolizumab - Nivolumab |  |
| **Gastric cancer**  **Gastric cancer** | Cisplatin (available as a generic drug)  Oxaliplatin  Fluorouracil  Capecitabine  Docetaxel  Epirubicin  Irinotecan  Paclitaxel | HER2-targeted therapy:   - Trastuzumab   Anti-angiogenesis therapy:   - Ramucirumab | Anti-PD-1 antibodies:   - Pembrolizumab |  |
