# Supplementary material for: Current trends and future prospects of drug repositioning in gastrointestinal oncology
Source: Front Pharmacol. 2024 Jan 4;14:1329244. doi: 10.3389/fphar.2023.1329244 (PMC10794567; doi:10.3389/fphar.2023.1329244)
Supplement: Supplementary file 2 [file Table2.docx]

**Table S2:** List of repurposed unapproved or withdrawn drugs/natural components targeting GI-related cancers

| **Drug name** | **Mechanism of action** | **Original indication** | **Proposed indication** | **Reported targets/pathways** | **Status** | **Ref** |
| --- | --- | --- | --- | --- | --- | --- |
| A939572 | Inhibition of proliferation, migration and invasion | A bioavailable SCD1 inhibitor | Hepatocellular Carcinoma | SCD1, ER stress | *In vivo* (patient-derived tumor xenograft model) | [(Ma et al., 2017)](https://paperpile.com/c/VjiOuK/3Xco) |
| Chrysin | Induction of apoptosis | A flavone found in honey and inhibits HK2 | Hepatocellular Carcinoma | Decreases glucose uptake and lactate production in HCC cells, reduces HK2 | *In vitro* (HCC cell lines), and *in vivo* (mouse xenograft model) | [(Xu et al., 2017)](https://paperpile.com/c/VjiOuK/q5c5) |
| CPD23 | Partial inhibition of tumor growth | A synthetic antibiotic and selenium analogue of CB839 that is designed to improve *in vivo* anticancer activity | Hepatocellular Carcinoma | Causes tumor tissue damage and prolongs survival, GLS1 | *In vitro* (CRC and LC cell lines), and *in vivo* (xenograft mouse model) | [(Du et al., 2022)](https://paperpile.com/c/VjiOuK/rcD4) |
| Emodin | Inhibition of Glycolysis and cell proliferation | Decreases glycolytic enzymes | Hepatocellular Carcinoma | HK2, PKM2, and LDHA | *In vitro* (HepG2 cell line) | [(Xing et al., 2018)](https://paperpile.com/c/VjiOuK/YqXJ) |
| Lanatoside C | Induction of apoptosis | Antiarrhythmic | Hepatocellular Carcinoma | MMP, PKCδ, AIF | *In vitro* (HCC cell lines), and *in vivo* (mouse xenograft model) | [(Chao et al., 2017; Sinn et al., 2019)](https://paperpile.com/c/VjiOuK/BjJi+wcwq) |
| Methyl jasmonate | Induction of apoptosis | A plant stress hormone to detach HK2 from VDAC | Hepatocellular Carcinoma | Reduction in mitochondrial transmembrane potential | *In vitro* (HCC cell lines), and *in vivo* (mouse xenograft model) | [(Li et al., 2017a)](https://paperpile.com/c/VjiOuK/6LKi) |
| ND-654 | Inhibition of proliferation | A liver-specific ACC inhibitor that allosteric inhibits ACC1 and ACC2 | Hepatocellular Carcinoma | Intensifies sorafenib effect | *In vivo (HCC mouse and rat models)* | [(Lally et al., 2019)](https://paperpile.com/c/VjiOuK/fnqC) |
| Shikonin | Inhibition of cell proliferation and glycolysis and induction of apoptosis | A naphthoquinone compound found in the roots of Lithospermum erythrorhizon that inhibits PKM2 | Hepatocellular Carcinoma | Enhances the drug sensitivity of HCC cells to sorafenib | *In vitro* (HCC cell lines), and *in vivo* (mouse xenograft model) | [(Liu et al., 2020c)](https://paperpile.com/c/VjiOuK/bgUA) |
| T0901317 | Inhibition of proliferation | A selective FXR and LXR agonist | Hepatocellular Carcinoma | LXR*α,* GLUT1 and MMP9 | *In vitro* (HepG2 cell line), and *in vivo* (mouse xenograft model) | [(Xiong et al., 2019)](https://paperpile.com/c/VjiOuK/B5M9) |
| V-9302 | Inhibition of proliferation, induction of cell death, and increase in oxidative stress | A competitive small molecule antagonist of transmembrane glutamine flux, that targets the amino acid transporter ASCT2 and affects glutamine metabolism | Hepatocellular Carcinoma | ASCT2 | *In vitro* (HCC cell lines), *in vivo* (mouse xenograft model) | [(Jin et al. 2020)](https://paperpile.com/c/VjiOuK/APet) |
| YM-53601 | Induction of cell arrest and cell death | A squalene synthase (SS) inhibitor, reduces plasma cholesterol and triglyceride levels in several animal species | Hepatocellular Carcinoma | Fatty acid metabolism | *In vitro* (HCC cell lines) | [(Ogura et al., 2018)](https://paperpile.com/c/VjiOuK/ergq) |
| 2-DG | Inhibition of proliferation and mobility | An analog of glucose to inhibit HK2 activity | Hepatocellular Carcinoma | Block the glycolysis, proliferation, migration, and invasion of HCC cells induced by ZMYND8 | *In vitro* (HCC cell lines) | [(Dou et al., 2021)](https://paperpile.com/c/VjiOuK/e4ji) |
| 3-Bromopyruvate | Increase chemosensitivity | A halogenated pyruvate derivative that inhibits HK2 | Hepatocellular Carcinoma | Glycolytic inhibitor | *In vitro* (HCC cell lines) | [(Sun et al., 2020)](https://paperpile.com/c/VjiOuK/T5ta) |
| AM404 | Mediating chemo-resistance and inhibition of metastasis | An active metabolite of paracetamol (acetaminophen) | Colorectal cancer | FBXL5 | *In vitro* (CRC cell lines) | [(Ahmed et al., 2019)](https://paperpile.com/c/VjiOuK/AAcS) |
| Artemisinin and related-derivatives | Induction of cell death | Malaria | Breast, colorectal, lung  cancer etc. | Ferroptosis, autophagy, oncosis,  anoikis | *In vitro* (CRC cell lines), and *in vivo* (mouse xenograft model) | [(Gong et al., 2022)](https://paperpile.com/c/VjiOuK/mhDp) |
| Brefeldin A | Inhibition of Cancer Stem Cell-Like properties, induction of autophagy | Brefeldin A | Colorectal cancer | MMP-9, Bip/Akt | *In vitro* (Colo 205 cell line) | [(Tseng et al., 2013)](https://paperpile.com/c/VjiOuK/vvMl) |
| Butyrate | Inhibition of proliferation and induction of apoptosis | Probiotic | Colorectal cancer | Inhibition of miR-92a | *In vitro* (CRC cell lines) | [(Hu et al., 2015)](https://paperpile.com/c/VjiOuK/TmcL) |
| Candesartan | Inhibition of proliferation | Hypertension | Colorectal cancer | Angiotensin II type 1 receptor | *In vitro* (CRC cell lines), and *in vivo* (mouse xenograft model) | [(Tabatabai et al., 2021)](https://paperpile.com/c/VjiOuK/hfoV) |
| Curcumin | Inhibition of cancer stem cell growth, regulation of self-renewing, regulation of growth factor, epigenetic modification, cell cycle arrest, induction of apoptosis, and regulation of structural integrity | It is an active ingredient (polyphenol) in turmeric. It induces apoptosis through a mitochondria-mediated pathway. | Colorectal cancer, Pancreatic Cancer | It induces apoptosis through a mitochondria-mediated pathway.  It also inhibits telomerase activity, hTERT, Wnt/β-catenin, Hippo/YAP. | Clinical trial: Phase 2 (NCT02439385; [NCT00094445](https://clinicaltrials.gov/ct2/show/NCT00094445)) | [(Park et al. 2013; Guo et al. 2013; Jeon et al. 2022)](https://paperpile.com/c/VjiOuK/qxfw+xrHh+eGcJ) |
| Genistein | Enabling replicative  immortality | Menopause, osteoporosis,  obesity | Colorectal, bladder, breast  cancer etc. | hTERT, Wnt/β-catenin | Clinical trial: Phase 1/2 (NCT01985763) | [(Pintova et al., 2019)](https://paperpile.com/c/VjiOuK/C4nG) |
| Pleconaril | Mediating chemo-resistance | Antiviral drug | Colorectal cancer | Capsid inhibitor | *In vitro* (HCT8 line) | [(Díaz-Carballo et al., 2015)](https://paperpile.com/c/VjiOuK/5HNb) |
| Tenofovir | Inhibition of proliferation, oxidative stress, and inflammation | Anti-retroviral (anti-HIV drug) | Colorectal cancer | Decreased Bcl-2 and cyclin D1 expression | *In vivo* (Wistar rat model) | [(Sherif et al., 2021)](https://paperpile.com/c/VjiOuK/0Tp3) |
| Auranofin | Induction of apoptosis, inhibition of distant organ metastasis and tumor growth | Rheumatoid  arthritis | Pancreatic Cancer | Inhibition of Trxnr1and HIF1α, caspase 3/7 activation, PARP cleavage | *In vitro* (PC cell lines), and *in vivo* (patient-derived xenograft and mouse models) | [(Rios Perez et al. 2019)](https://paperpile.com/c/VjiOuK/moR2) |
| Monensin | Inhibition of cell proliferation and migration, Induction of apoptosis and cell cycle arrest | Antibiotic | Pancreatic ductal adenocarcinoma | Inhibition of the E2F/DP1, STAT1/2, NF-kB, AP-1 and Elk-1/SRF pathways and suppression of EGFR expression | *In vitro* (pancreatic cancer cell lines), and *in vivo* (xenograft mouse model) | [(Wang et al., 2018a)](https://paperpile.com/c/VjiOuK/G6kQ) |
| Nitroxoline | Induction of cell cycle arrest and f apoptosis | Antiviral (Nelfinavir), Antibiotic (Nitroxoline) | Pancreatic ductal adenocarcinoma | Nitroxoline: ROS production, DNA damage response, mitochondrial depolarization and deregulation of cytosolic iron homeostasis | *In vitro* (AsPC-1 cell line) | [(Veschi et al., 2020)](https://paperpile.com/c/VjiOuK/dcAa) |
| Penfluridol | Induction of apoptosis, and cell cycle arrest | Psychosis | Pancreatic ductal adenocarcinoma | Apoptosis activation and cell cycle arrest, by targeting of protein phosphatase 2A (PP2A), SRC, AKT and p70S6k, Inhibition of JAK2–STAT3 and ERK/AKT signaling, ER stress | *In vitro* (pancreatic cancer cell lines), and *in vivo* (mouse model) | [(Ranjan et al., 2017)](https://paperpile.com/c/VjiOuK/jAyw) |
| Protoporphyrin IX | Induction of apoptosis | Antineovascularization agent (Verteporfin), Sensitizers in photodynamic therapy (protoporphyrin IX) | Pancreatic ductal adenocarcinoma | Activation of apoptosis via TAp73 activation, Inhibition of thioredoxin reductase Inhibition of Hippo/YAP signaling pathway | *In vitro* (pancreatic cancer cell lines) | [(Acedo et al. 2019)](https://paperpile.com/c/VjiOuK/i14Z) |
| Warfarin | Induction of apoptosis and inhibition of EMT | Anticoagulant | Pancreatic ductal adenocarcinoma | Inhibition of Gas6-induced Axl signaling activated apoptosis and suppressed EMT, Immunomodulatory response by inhibition of MerTK signaling | Withdrawn ([NCT03536208](https://clinicaltrials.gov/ct2/show/NCT03536208)) | [(Rebelo et al., 2021)](https://paperpile.com/c/VjiOuK/P3ml) |
| Propagermanium | Inhibition of metastasis | Chronic hepatitis type B | Gastric cancer | Glycosylphosphatidylinositol-anchored proteins, CCR, CCL2/CCR2 signaling pathway | Clinical trial: Phase 2 (UMIN000017123) | [(Yumimoto et al. 2019)](https://paperpile.com/c/VjiOuK/CJmJ) |
| TPCA‑1 | Induction of apoptosis | Antibiotic | Esophageal cancer | Inhibitor of IKK-2, NF-κB pathway | *In vitro* (esophageal squamous cell carcinoma cell lines) | [(Li et al. 2022)](https://paperpile.com/c/VjiOuK/DihU) |
